# Quality of reporting of integrative Chinese and Western medicine intervention in randomized controlled trials of ulcerative colitis: a review

**DOI:** 10.1186/s13643-023-02402-2

**Published:** 2023-12-08

**Authors:** Jialing Zhang, Jiashuai Deng, Nana Wang, Ping Wang, Ji Li, Yunhai Wang, Wanting Cui, Feng Liang, Peijin Chen, Juan Wang, Fei Han, Chun Pong Chan, Aiping Lyu, Zhaoxiang Bian, Xuan Zhang

**Affiliations:** 1 Hong Kong Chinese Medicine Clinical Study Centre, Chinese EQUATOR Centre, School of Chinese Medicine, Chinese Clinical Trial Registry (Hong Kong), Hong Kong Baptist University, HKSAR, China; 2 Centre for Chinese Herbal Medicine Drug Development, Hong Kong Baptist University, HKSAR, China; 3 School of Traditional Chinese Medicine, Beijing University of Chinese Medicine, Beijing, China; 4 China Academy of Chinese Medical Sciences, Xiyuan Hospital, Beijing, China; 5 Department of Pediatrics, China Academy of Chinese Medical Sciences, Guang’anmen Hospital, Beijing, China; 6 College of Traditional Chinese Medicine, Tianjin University of Traditional Chinese Medicine, Tianjin, China; 7 Department of Computer Science, Faculty of Science, Hong Kong Baptist University, HKSAR, China

**Keywords:** Ulcerative colitis (UC), CONSORT guideline, Randomized controlled trial (RCT), Reporting quality, Integrative Chinese and Western medicine (ICWM)

## Abstract

**Background:**

Integrative Chinese and Western medicine (ICWM) is commonly used for the treatment of ulcerative colitis (UC) in clinical practice. However, it is unclear whether the details of ICWM interventions, such as selection rationale, implementation design, and potential interactions, were adequately reported. Therefore, this study aimed to assess the quality of reporting in the ICWM interventional randomized controlled trials (RCTs) of UC and to identify the common problems if any.

**Methods:**

Through a search of 10 international electronic databases, we identified RCTs of UC with ICWM interventions published in English or Chinese from the inception date of each database up to 16 June 2023. Literature screening was strictly conducted based on the inclusion and exclusion criteria of the Population, Concept, and Context (PCC) framework. The general characteristics of the included studies were described. The quality of reporting was assessed according to three checklists, including the CONSORT (Consolidated Standards of Reporting Trials) with 36 items (except for one item 1b about abstract), the CONSORT for Abstracts (17 items), and a self-designed ICWM-related checklist (27 items covering design rationale, intervention details, outcome assessments, and analysis). The reporting scores of RCTs published before and after 2010 were compared.

**Results:**

A total of 1458 eligible RCTs were included. For the reporting compliance, the median score (interquartile ranges) of the CONSORT (72 score in total), the CONSORT for Abstract (34 score), and ICWM-related (54 score) items was 21 (18–25), 13 (12–15), and 18 (15–21), respectively. Although the time period comparisons showed that reporting quality of included publications improved significantly after the CONSORT 2010 issued (*P* < 0.01), more than 50% of items were evaluated as poor quality (reporting rate < 65%) among each checklist, especially in the CONSORT for Abstract and ICWM-specific items.

**Conclusion:**

Although CONSORT appears to have enhanced the reporting of RCTs in UC, the quality of ICWM specifics is variable and in need of improvement. Reporting guidelines of the ICWM recommendations should be developed to improve their quality.

**Supplementary Information:**

The online version contains supplementary material available at 10.1186/s13643-023-02402-2.

## Introduction

Ulcerative colitis (UC) is a global disease, the incidence and prevalence of which have increased in several regions of the world [1, 2]. As an immune-mediated chronic inflammatory bowel disease, UC is characterized by continuous and superficial inflammation of the colon. However, its etiology and pathogenesis have not yet been determined and are generally considered to be influenced by genetic, environmental, and microbial factors [3]. The treatment goal of UC is to achieve symptomatic relief, endoscopic healing, normalization of serum and fecal markers, absence of disability, restoration of quality of life, and normal growth in children [4].

Conventional treatments control symptoms through pharmacotherapy, including aminosalicylates, corticosteroids, immunomodulators, and biologics, with other general measures or surgical resection if necessary [5]. Aminosalicylates are known to be the first-line treatment option for mild to moderate UC [6], but they may cause gastrointestinal discomfort such as nausea, abdominal pain, and diarrhea [7, 8]. Corticosteroids are a kind of treatment selection for UC patients who have inadequate response to mesalazine, however, their long-term treatment is not recommended due to significant adverse effects such as an increased risk of mortality, infection, diabetes mellitus, hypertension, and osteoporosis [7, 9]. Immunomodulators and biologics are beneficial to patients with moderate to severe disease activity, corticosteroid dependence, or those at high risk of colectomy, but up to 74% of UC patients may lose response over time [10]. Limitations such as inadequate response, side effects, dependence, and drug resistance to Western medicine (WM) remain to be solved [11, 12].

Chinese medicine (CM), a medical system based on distinctive Chinese cultural theories and practices, has been increasingly introduced into the treatment of UC, especially in Asia [13]. CM treatment mainly includes Chinese herbal formula (oral or rectal), acupuncture, and moxibustion. Due to its multi-targeted mode of action, CM has unique advantages for treating inflammatory bowel diseases, including maintaining intestinal integrity, reducing inflammation, and decreasing oxidative stress, with minor side effects [14]. With the extensive studies on UC in recent years, it is confirmed that both CM and WM have unique advantages in UC management.

In China, most patients of UC preferred Integrated Chinese and Western medicine (ICWM) therapy over CM or WM alone. Several reviews supported the promising effect and few side effects of ICWM for UC [15, 16], but most of the included trials had a high risk of bias and major methodological deficiencies, including an insufficient description of the randomization process, lacking appropriate therapeutic endpoints, and missing power calculations. Although the quality of reporting in RCTs in medical sciences has been discussed, the quality of reporting in RCTs on the treatment of UC with ICWM has not yet been assessed after the publication of the CONSORT (Consolidated Standards of Reporting Trials) 2010 Statement. Furthermore, it is unclear whether the details of ICWM interventions, such as selection rationale, indications and timing of interventions, implement design and potential interactions, were adequately reported in current randomized clinical trials (RCTs). Therefore, this review aimed to assess the quality of reporting in RCTs of ICWM for UC, based on the checklists of CONSORT 2010 [17] and a self-designed ICWM-specific checklist.

## Materials and methods

### Eligibility criteria

This study included ICWM interventional RCTs of UC published in English or Chinese from the date of inception for each database up to 16 June 2023. We included studies on subjects given the diagnosis of UC defined by clear diagnostic criteria or references, regardless of age, gender, course of disease, and severity. The ICWM intervention is defined as the combination of CM therapies and WM treatments. Specifically, we included a wide range of CM interventions, such as herbal medicines, acupuncture, and moxibustion, while only pharmacological therapy of WM was in the intervention group. There were no limitations in the types of control groups and the assessed outcomes. Repeat publications, non-randomized or non-controlled trials, quasi-randomized controlled trials, non-ICWM interventional trials, study protocols, reviews, observational studies, case reports, abstracts, full-text reports not found, and non-human studies were excluded. Table 1 provides details on the inclusion and exclusion criteria of PCC used in this review. The conduction of this review is referred to the Chapter 11-Scoping Reviews of Joanna Briggs Institute Reviewer's Manual for Evidence Synthesis [17, 18], also with some modifications in terms of format as this review focused on the quality assessment.

**Table 1:** Inclusion and exclusion criteria based on the Population, Concept, and Context (PCC) framework

	Inclusion criteria	Exclusion criteria
Population	Studies were included if they: • Included subjects given the diagnosis of UC defined by clear diagnostic criteria or references, regardless of age, gender, course of disease, and severity	Focus exclusively on: • Non-human studies
Concept	Studies were included if they: • Used ICWM intervention in the management of UC. The ICWM intervention is defined as the combination of CM therapies and WM treatments. Specifically, we included a wide range of CM interventions, such as herbal medicines, acupuncture, and moxibustion, while only pharmacological therapy of WM was in the intervention group. *AND* • No limitations in the types of control groups and the assessed outcomes	Focus exclusively on: • Non-ICWM interventional trials. *OR* • Non-randomized or quasi-randomized controlled trials. *OR* • Non-controlled trials.* OR* • Observational studies. *OR* • Case reports. *OR* • Study protocols. *OR* • Reviews
Context	Studies were included if they: • Published in English or Chinese.* AND* • Published on/before 16 June 2023	Focus exclusively on: • Abstracts or full-text reports not found. *OR* • Repeat publications

### Search strategy

A systematic search was conducted on 17 June 2023 for the following databases: MEDLINE < 1946 to 16 June 2023 > , Embase < 1974 to 16 June 2023 > , CENTRAL (Cochrane Central Register of Controlled Trials) < May 2023 > , Web of Science < 1900 to 16 June 2023 > , CINAHL < 1937 to 16 June 2023 > , AMED (Allied and Complementary Medicine Database) < 1985 to May 2023 > , CNKI (China National Knowledge Infrastructure) < 1979 to 16 June 2023 > , Wanfang < 1979 to 16 June 2023 > , VIP (Chinese Science and Technology Periodical Database) < 1989 to 16 June 2023 > , CBM (Chinese Biomedical Literature Database) < 1978 to 16 June 2023 > . The search terms were “ulcerative colitis”, “inflammatory bowel disease”, “randomized controlled trial”, “random”, “Chinese medicine”, “herbal”, and “drug”. The detailed search strategy is presented in Additional file 1: Appendix 1.

### Study selection

Endnote 20 (Clarivate, Philadelphia, PA 19130) was used for de-duplication, title and abstract screening. After de-duplication, four review authors (JLZ, NNW, FL, and PJC) independently screened titles and abstracts of the retrieved records based on the inclusion and exclusion criteria, and another two review authors (JSD and JW) conducted a second check. Full text of potentially relevant papers were reviewed (JLZ, NNW, FL, and PJC) and double-checked (JSD and JW) for further assessment of eligibility. Differences of opinion were settled by consensus.

### Data extraction

We developed a data extraction Microsoft Excel form to extract data on general characteristics of included records, including study title, publication year and language, information of corresponding author(s), types of journals, types of study design (e.g., assignment, randomization, blinding, sample size and participating centers), features of interventions, types of participant(s) and control(s), period of treatment and follow-up (if any), as well as the categories of diagnosis criteria, outcome(s) and study conclusions. We piloted the extraction form on a random sample of ten included articles and achieved consistency in data item interpretations. Then, four trained authors (JLZ, FL, NNW, and JSD) independently extracted the data, and another two review authors (XZ and JW) conducted a second check. Disagreements were resolved by discussion.

### Reporting quality assessment

The reporting quality of included studies was evaluated according to a standard checklist of the CONSORT 2010 statement, of which the checklist of the CONSORT for Abstract was extracted for independent evaluation. For rating the CONSORT items, the assessment rules were referred to the CONSORT 2010 statement (including the CONSORT for Abstract) with its explanation and elaboration document which provides the definitions and rationale for each checklist item and examples of good reporting (e.g., scored as 2 points) [19, 20]. The total score of the CONSORT checklist and CONSORT for the abstract checklist was 72 and 34, respectively.

A specially designed checklist comprised of 27 items related to specific characteristics of ICWM trials (Table 2) was developed by five researchers (XZ, JL, PW, FH, and ZXB) based on an internal discussion. This list focused on the identification of critical issues in the procedure of ICWM design, implementation, and assessment, particularly in the selection rationale, details of therapy combination, and the efficacy assessment. Each item/question was scored in terms of three possibilities: “2” for “fully reported”, “1” for “partially reported”, and “0” for “not reported” or “not applicable”. The total score of the ICWM-specific checklist was 54. For rating the ICWM items, the details of scoring rules are presented in Additional file 1: Appendix 2 which includes the explanations for each question and examples of eligible reporting. The quality assessment was independently conducted by one review author and verified by another review author. A total of four authors (YHW, JSD, NNW, and WTC) participated in the quality assessment. Possible disagreements were resolved with the consultation of the third senior review authors (XZ or ZXB).

**Table 2:** Questions for assessing the reporting of ICWM-specific items

Item no	Specifics
Q1	Whether the feature of ICWM was presented in the section of “Title” (e.g., generalized term of ICWM, or specific CM and WM interventions provided in the title)?
Q2	Whether the eligibility criteria of participants include both Chinese and Western medical diagnosis in Methods of Abstract?
Q3	Whether the study objectives or hypotheses were focused on the ICWM interventions in the Abstract?
Q4	Whether the outcome measures included both CM and WM-related endpoints in the Abstract?
Q5	Whether the effect of studied ICWM interventions was reported in the Conclusion of the Abstract?
Q6	Whether the features or design of the ICWM study were reflected in Keywords?
Q7	Whether the reason/rationale about ICWM intervention for the study design was reported in Background?
Q8	Whether any necessity/advantage about ICWM intervention was reported in the Background?
Q9	Whether the objectives or hypotheses were focused on the ICWM interventions in the Background (e.g., improve the efficacy/safety, or reduce the side effects)?
Q10	Whether the eligibility criteria of participants include both Chinese and Western medical diagnosis in Methods?
Q11	Whether the specific information of disease (e.g., classification of disease, treatment points, stages of diseases) of the ICWM was reported in Methods?
Q12	Whether any specific criteria related ICWM in the selection of study centers?
Q13	Whether the specific type/way of integration of CM and WM interventions (such as overlying, one-after-another, or add-on design) was reported in Methods?
Q14	In the ICWM group, whether CM intervention(s) was reported with sufficient details to allow replication, including how and when they were administered?
Q15	In the ICWM group, whether WM intervention(s) was reported with sufficient details to allow replication, including how and when they were administered?
Q16	Whether the rationale for the choice of the control group(s) was provided?
Q17	In the control group, whether sufficient details were reported to allow replication?
Q18	Whether any description of treatment providers’ background (e.g., qualification and/or experiences in ICWM, or whether the providers conducted CM and WM separately)?
Q19	Whether any measures were adopted to evaluate or improve the compliance of participants?
Q20	Whether the outcome measures included both CM and WM-related endpoints in Methods?
Q21	For the studies with open label, whether any reasons or explanations for such design was reported?
Q22	In the control group(s), did the placebo of WM invention(s) was included? If so, whether sufficient details were provided?
Q23	In the control group(s), did the placebo of CM invention(s) was included? If so, whether sufficient details were provided?
Q24	In the section of Results, whether any information about the participants exposed to ICWM treatment prior to recruitment was mentioned in the baseline data?
Q25	Whether any discussion about external validity of ICWM results reported, particularly in different environments?
Q26	Whether interpretation and significance of studied ICWM interventions for the disease was reported in the Discussion?
Q27	Whether any potential conflicts of interests were clearly reported?

*ICWM* Integrative Chinese and Western medicine*, CM* Chinese medicine*, WM Western medicine*

### Data analysis

As this review is focused on reporting characteristics and quality evaluation, we thereby applied frequency and percentage to present categorical variables, and mean (standard deviation) or median (interquartile range, IQR) to present continuous variables in the section of “Results”. For individual item of reporting quality, the compliance rate was calculated with the number of items acquired “2” based on the total number of included reports, which was further categorized as three levels: excellent compliance (> 90%), good compliance (between 65 and 90%), and poor compliance (< 65%). The total scores of the CONSORT, CONSORT for Abstract, and ICWM-specific checklists of RCTs published before and after 2010 were compared with Student’s *t* test or Mann–Whitney *U* test. Statistics analyses were performed using SPSS software (version 28.0). Statistical significance was defined as two-sided *P* value < 0.05.

## Results

### Literature search

**Fig. 1:**
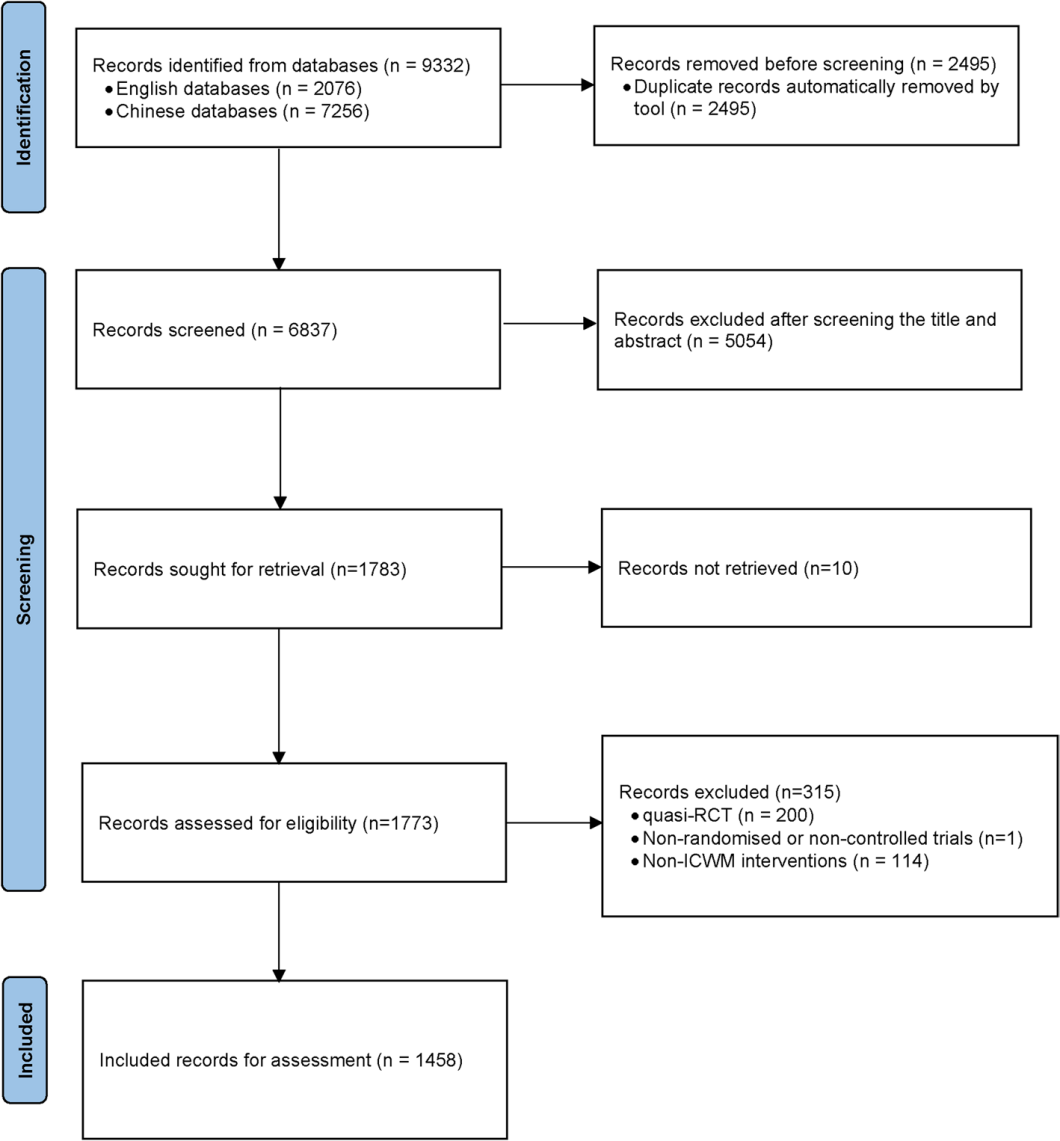
Flow chart of the literature screening and selection

The flowchart of the selection and screening process is shown in Fig. 1. Briefly, the electronic search yielded 9332 records, after removing duplicates and screening titles and abstracts, a total of 1773 reports were identified for the full-text assessment. Finally, we included 1458 eligible RCTs for analysis, of which 1385 articles with abstracts and 73 without abstracts.

### Characteristics of included trials

**Fig. 2:**
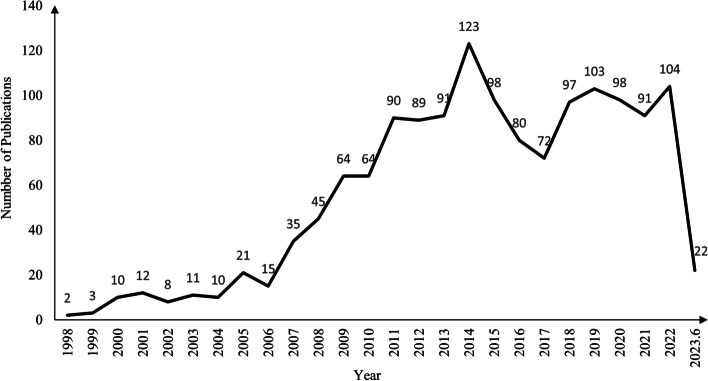
The number of ICWM interventional RCTs of UC publications between Jan 1998 to June 2023

A total of 1458 RCTs of ICWM for UC were included between January 1998 to June 2023. The number of these studies increased gradually during the first 10 years and then presented a rapid increase starting from 2009 (Fig. 2). The most common design of included studies was a single-center (97.94%), two parallel arms (94.10%), a sample size of 51–100 (70.30%), Chinese herbal formula (90.53%) as CM treatment, and intervention period within 30 days (48.49%). There were 575 (39.44%) studies that applied CM diagnosis when recruited UC participants, while only 349 (23.94%) adopted CM-related outcomes to assess the efficacy of treatments. Around 98.63% (1438) trials concluded a confirmed efficacy of ICWM for UC. However, the missing reporting is common for several critical aspects, such as 97.94% of trials did not whether to adopt blinding or not, and 71.88% of trials did not specify the studied phase(s) of UC (e.g., active, remission, or both). Details are shown in Table 3.

**Table 3:** Characteristics of included articles (*n* = 1458)

Characteristics	*N* (%)
Study center	
Single center	1428 (97.94)
Multicenter	22 (1.51)
Not reported	8 (0.55)
Journal type	
English journal, with impact factor 5–10	1 (0.07)
English journal, with impact factor 3–5	2 (0.14)
English journal, with impact factor < 3	3 (0.21)
Chinese core journal	66 (4.53)
Chinese non-core journal	1386 (95.06)
Sample size	
> 300	6 (0.41)
101–300	295 (20.23)
51–100	1025 (70.30)
≤ 50	132 (9.05)
UC stages of trial participants	
Active	368 (25.24)
Remission	19 (1.30)
Both	23 (1.58)
Not specified	1048 (71.88)
Number of groups	
2	1372 (94.10)
3	79 (5.42)
4 or above	7 (0.48)
Blinding	
Single-blinded	9 (0.62)
Double-blinded	18 (1.23)
Open-label	3 (0.21)
Not reported	1428 (97.94)
Intervention type of CM	
Chinese herbal formulas	1320 (90.53)
Single herbs	28 (1.92)
Moxibustion	20 (1.37)
Acupuncture	12 (0.82)
Catgut-embedding therapy	11 (0.75)
Massage	2 (0.14)
Autohemotherapy at acupoint	1 (0.07)
Complex intervention^a^	64 (4.39)
Intervention period	
≤ 30 days	707 (48.49)
31–60 days	489 (33.54)
61–90 days	167 (11.45)
> 90 days	27 (1.85)
Not reported	70 (4.80)
Follow-up period	
≤ 90 days	66 (4.53)
91–180 days	109 (7.48)
181–360 days	70 (4.80)
> 360 days	12 (0.82)
Not reported	1201 (82.37)
Type of controls	
Integrated CM and WM	78 (5.35)
Including placebo	17 (1.17)
Solely WM as control	1359 (93.21)
Solely CM as control	4 (0.27)
Diagnostic criteria of UC	
Both CM and WM	575 (39.44)
CM	2 (0.14)
WM	881 (60.43)
CM-related outcomes	
CM pattern score	303 (20.78)
CM symptoms^b^	46 (3.16)
Not reported	1109 (76.06)
Conclusions on the efficacy of ICWM	
Confirmed efficacy	1438 (98.63)
Beneficial	20 (1.37)

^a^Details are shown in Additional file 1: Appendix 3

^b^CM symptoms included the tongue and pulse manifestations

### Reporting completeness and features

The results of adherence to the CONSORT, the CONSORT for Abstract, and ICWM-specific checklist items are presented in Tables 4, 5, and 6. For the completeness of the CONSORT checklist, the median (IQR) reporting score was 21 (18–25). Specifically, the reporting quality was excellent (> 90%) in 4 items (2a, 6b 15, and 22); good (65–90%) in 5 items (4b, 5, 11b, 13a, and 16); and poor (< 65%) in 27 items (1a, 2b, 3a, 3b, 4a, 6a, 7a, 7b, 8a, 8b, 9, 10, 11a, 12a, 12b,13b, 14a, 14b,17a, 17b, 18,19, 20, 21, 23, 24, and 25). For the CONSORT for Abstract, the reporting score was 13 (12–15). The quality of reporting was excellent (> 90%) in 3 items (3, 6, and 15); good (65–90%) in 1 item (10); and poor (< 65%) in 13 items (1, 2, 4, 5, 7, 8, 9, 11, 12, 13, 14, 16, and 17).

**Table 4:** Reporting assessment of included studies based on the CONSORT items (*n* = 1458)

Section/topic	Item number and description	Fully reported	Partially reported	Not reported
Title and abstract	1a. Identification as a randomized trial in the title	13 (0.89)	–	1445 (99.11)
	1b. Structured summary of trial design, methods, results, and conclusions	See Table 5		
Introduction				
Background	2a. Scientific background and explanation of rationale	1356 (93.00)	–	102 (7.00)
Objectives	2b. Specific objectives or hypotheses	535 (36.69)	–	923 (63.31)
Methods				
Trial design	3a. Description of trial design (such as parallel, factorial) including allocation ratio	4 (0.27)	63 (4.32)	1391 (95.40)
	3b. Important changes to methods after trial commencement (such as eligibility criteria), with reasons	13 (0.89)	–	1445 (99.11)
Participants	4a. Eligibility criteria for participants	861 (59.05)	458 (31.41)	139 (9.53)
	4b. Settings and locations where the data were collected	1046 (71.74)	91 (6.24)	321 (22.02)
Interventions	5. The interventions for each group with sufficient details to allow replication, including how and when they were actually administered	1094 (75.03)	358 (24.55)	6 (0.41)
Outcomes	6a. Completely defined pre-specified primary and secondary outcome measures, including how and when they were assessed	3 (0.21)	596 (40.88)	859 (58.92)
	6b. Any changes to trial outcomes after the trial commenced, with reasons	1458 (100)	–	–
Sample size	7a. How sample size was determined	5 (0.34)	3 (0.21)	1450 (99.45)
	7b. When applicable, explanation of any interim analyses and stopping guidelines	41 (2.81)	–	1417 (97.19)
Sequence generation	8a. Method used to generate the random allocation sequence	522 (35.80)	835 (57.27)	101 (6.93)
	8b. Type of randomization; details of any restriction (such as blocking and block size)	11 (0.75)	511 (35.05)	936 (64.20)
Allocation concealment mechanism	9. Mechanism used to implement the random allocation sequence (such as sequentially numbered containers), describing any steps taken to conceal the sequence until interventions were assigned	16 (1.10)	507 (34.77)	935 (64.13)
Implementation	10. Who generated the random allocation sequence, who enrolled participants, and who assigned participants to interventions	6 (0.41)	–	1452 (99.59)
Blinding	11a. If done, who was blinded after assignment to interventions (for example, participants, care providers, those assessing outcomes) and how ^a^	1 (0.07)	9 (0.62)	1445 (99.31)
	11b. If relevant, description of the similarity of interventions ^b^	13 (76.47)	4 (23.53)	–
Statistical methods	12a. Statistical methods used to compare groups for primary and secondary outcomes	16 (1.10)	1185 (81.28)	257 (17.63)
	12b. Methods for additional analyses, such as subgroup analyses and adjusted analyses	0 (0)	–	1458 (100)
Results				
Participant flow	13a. For each group, the numbers of participants who were randomly assigned, received intended treatment, and were analyzed for the primary outcome	1278 (87.65)	173 (11.87)	7 (0.48)
	13b. For each group, losses and exclusions after randomization, together with reasons	53 (3.64)	31 (2.13)	1374 (94.24)
Recruitment	14a. Dates defining the periods of recruitment and follow-up	238 (16.32)	1034 (70.92)	186 (12.76)
	14b. Why the trial ended or was stopped	0 (0)	–	1458 (100)
Baseline data	15. A table showing baseline demographic and clinical characteristics for each group	1456 (99.86)	–	2 (0.14)
Numbers analyzed	16. For each group, the number of participants (denominator) included in each analysis and whether the analysis was by originally assigned groups	1279 (87.72)	–	179 (12.28)
Outcomes and estimation	17a. For each primary and secondary outcome, results for each group, and the estimated effect size and its precision (such as 95% confidence interval)	0 (0)	1450 (99.45)	8 (0.55)
	17b. For binary outcomes, the presentation of both absolute and relative effect sizes is recommended	0 (0)	–	1458 (100)
Ancillary analyses	18. Results of any other analyses performed, including subgroup analyses and adjusted analyses, distinguishing pre-specified from exploratory	0 (0)	–	1458 (100)
Harms	19. All-important harms or unintended effects in each group	550 (37.72)	23 (1.58)	885 (60.70)
Discussion				
Limitations	20. Trial limitations, addressing sources of potential bias, imprecision, and, if relevant, multiplicity of analyses	193 (13.24)	–	1265 (86.76)
Generalizability	21. Generalizability (external validity, applicability) of the trial findings	9 (0.62)	–	1449 (99.38)
Interpretation	22. Interpretation consistent with results, balancing benefits and harms, and considering other relevant evidence	1458 (100)	–	–
Other information				
Registration	23. Registration number and name of trial registry	3 (0.21)	–	1455 (99.79)
Protocol	24. Where the full trial protocol can be accessed, if available	1 (0.07)	1 (0.07)	1456 (99.86)
Funding	25. Sources of funding and other support (such as the supply of drugs), role of funders	3 (0.21)	282 (19.34)	1173 (80.45)

^a^3 studies were not calculated for open-label

^b^1441 studies were not calculated as it is not placebo design

**Table 5:** Reporting assessment of included studies based on the CONSORT for Abstract checklist (*n* = 1385)

Section/topic	Item number and description	Fully reported	Partially reported	Not reported
Title	1. Identification of the study as randomized	14 (1.01)	–	1371 (98.99)
Authors	2. Contact details for the corresponding author	234 (16.90)	1151 (83.10)	–
Trial design	3. Description of the trial design (e.g., parallel, cluster, non-inferiority)	1301 (93.34)	–	84 (6.66)
Participants	4. Eligibility criteria for participants and the settings where the data were collected	314 (22.67)	1071 (77.33)	
Interventions	5.Interventions intended for each group	893 (64.48)	391 (28.23)	101 (7.29)
Objective	6.Specific objective or hypothesis	1374 (99.21)	–	11 (0.79)
Outcome	7.Clearly defined primary outcome for this report	0 (0)	761 (54.95)	624 (45.05)
Randomization	8.How participants were allocated to interventions	312 (22.53)	957 (69.10)	116 (8.38)
Blinding	9.Whether or not participants, caregivers, and those assessing the outcomes were blinded to group assignment	1 (0.07)	12 (0.87)	1372 (99.06)
Numbers randomized	10.Number of participants randomized to each group	1097 (79.21)	12 (0.87)	276 (19.93)
Recruiting objects	11.Clinical Trial Status	0 (0)	714 (51.55)	671 (48.45)
Numbers analyzed	12.Number of participants analyzed in each group	44 (3.18)	6 (0.43)	1335 (96.39)
Outcome	13.For the primary outcome, a result for each group and the estimated effect size and its precision	0 (0)	63 (4.55)	1322 (95.45)
Harms	14.Important adverse events or side effects	150 (10.83)	188 (13.57)	1047 (75.60)
Results	15.Generalized interpretation of outcome	1375 (99.28)	–	10 (0.72)
Trial registration	16.Registration number and name of trial register	0 (0)	1 (0.07)	1384 (99.93)
Funding	17.Source of funding	2 (0.14)	284 (20.51)	1100 (79.42)

**Table 6:** Reporting assessment of included studies based on ICWM-specific items (*n* = 1458)

Item number and description	Fully reported	Partially reported	Not reported
Q1. Whether the feature of ICWM was presented in the section of “Title” (e.g., generalized term of ICWM, or specific CM and WM interventions provided in the title)?	1027 (70.44)	–	431 (29.56)
Q2. Whether the eligibility criteria of participants included both Chinese and Western medical diagnosis in Methods of Abstract?^a^	250 (18.05)	1135 (81.95)	-
Q3. Whether the study objectives or hypotheses were focused on the ICWM interventions in Abstrac?^a^	851 (61.44)	–	534 (38.56)
Q4. Whether the outcome measures included both CM and WM-related endpoints in the Abstract?^a^	232 (16.75)	528 (38.12)	625 (45.13)
Q5. Whether the effect of studied ICWM interventions was reported in Conclusion of Abstract?^a^	929 (67.08)	–	456 (32.92)
Q6. Whether the features or designs of the ICWM study reflected in Keywords?	1040 (71.33)	366 (25.10)	52 (3.57)
Q7. Whether the reason/rationale for ICWM intervention for the study design was reported in Background?	243 (16.67)	457 (31.34)	758 (51.99)
Q8. Whether any necessity/advantage of ICWM intervention was reported in the Background?	114 (7.82)	–	1344 (92.18)
Q9. Whether the objectives or hypotheses focused on the ICWM interventions in the Background (e.g., improve the efficacy/safety, or reduce the side effects)?	305 (20.92)		1153 (79.08)
Q10. Whether the eligibility criteria of participants include both Chinese and Western medical diagnosis in Methods?	339 (23.25)	236 (16.19)	883 (60.56)
Q11. Whether the specific information of disease (e.g., classification of disease, treatment points, stages of diseases) of the ICWM was reported in Methods?	410 (28.12)	–	1048 (71.88)
Q12. Whether any specific criteria related to ICWM in the selection of study centers?	39 (2.67)	–	1419 (97.33)
Q13. Whether the specific type/way of integration of CM and WM interventions (such as overlying, one-after-another, or add-on design) was reported in Methods?	1336 (91.63)	–	122 (8.37)
Q14. In the ICWM group, whether CM intervention(s) reported with sufficient details to allow replication, including how and when they were administered?	1136 (77.91)	–	322 (22.09)
Q15. In the ICWM group, whether WM intervention(s) was reported with sufficient details to allow replication, including how and when they were administered?	1343 (92.11)	–	115 (7.89)
Q16. Whether the rationale for the choice of the control group(s) was provided?	1458 (100)	–	-
Q17. In the control group, whether sufficient details were reported to allow replication?	1325 (90.88)	–	133 (9.12)
Q18. Whether any description of treatment providers’ background (e.g., qualification and/or experiences in ICWM, or whether the providers conducted CM and WM separately)?	12 (0.82)	–	1446 (99.18)
Q19. Whether any measures were adopted to evaluate or improve the compliance of participants?	4 (0.27)	–	1454 (99.73)
Q20. Whether the outcome measures included both CM and WM related endpoints in Methods?	349 (23.94)	–	1109 (76.06)
Q21. For the studies with open label, whether any reasons or explanations for such design was reported?^b^	0 (0)	–	3 (100)-
Q22. In the control group(s), did the placebo of WM invention(s) was included? If so, whether sufficient details were provided?^c^	8 (47.06)	–	9 (52.94)
Q23. In the control group(s), did the placebo of CM invention(s) was included? If so, whether sufficient details were provided?^c^	16 (94.12)	–	1 (5.88)
Q24. In the section of Results, whether any information about the participants exposed to ICWM treatment prior to recruitment was mentioned in the baseline data?	0 (0)	–	-
Q25. Whether any discussion about external validity of ICWM results was reported, particular in different environments?	0 (0)	–	-
Q26. Whether interpretation and significance of studied ICWM interventions for the disease was reported in the Discussion?	306 (20.99)	567 (38.89)	585 (40.12)
Q27. Whether any potential conflicts of interest were clearly reported?	15 (1.03)	36 (2.47)	1407 (96.50)

^a^73 studies were not calculated as they did not have an Abstract

^b^3 studies were not calculated for open-label

^c^1441 studies were not calculated as it is not a placebo design

The reporting score of the ICWM-specific items was 18 (15–21). Four items were “excellent” (Q13, Q15, Q16, Q17, Q23), and “good” reporting in 5 items (Q1, Q5, Q6, Q14). The remaining 18 items were reported poorly (Q2, Q3, Q4, Q7, Q8, Q9, Q10, Q11, Q12, Q18, Q19, Q20, Q21, Q22, Q24, Q25, Q26, Q27), of which 16 items showed extremely low (< 33%), particular in the rationale for ICWM design with definite objectives or hypotheses, specific timepoint/stage of integrative therapy for UC, CM-related diagnosis criteria and outcome(s), discussion about the internal and external validity of ICWM results and value of ICWM design were insufficient or not articulated in most studies. The total scores of the CONSORT, the CONSORT for Abstract, and ICWM-specific checklist items were significantly improved after 2010 (all *P* < 0.01; Table 7).

**Table 7:** Overall reporting quality scores for included studies, by subgroup

Year of publication(n)	For CONSORT items^1^	For CONSORT for Abstract items^2^	For ICWM-specific items^3^
1998–2010 (*n* = 300)	17 (15–20)	11 (9–12)	16 (13–19)
2011–2022 (*n* = 1158)	23 (20–26)^*,a^	14 (12–15)^*,b^	19 (15–23)^*,c^
Total reports (*n* = 1458)	21 (18–25)	13 (12–15)	18 (15–21)

^1^A perfect score is 72 for the CONSORT checklist

^2^A perfect score is 34 for the CONSORT for Abstract checklist

^3^A perfect score is 54 for the ICWM-specific checklist

^*^Compared with 1998–2010, *P* < 0.01

^a^Compared with the group before 2010, the enhancement of CONSORT was 4.92 (4.4–5.43)

^b^ Compared with the group before 2010, the enhancement of CONSORT for Abstract was 3.02 (2.69–3.35)

^c^ Compared with the group before 2010, the enhancement of ICWM-specific items was 3.03 (2.48–3.59)

## Discussion

ICWM has been widely applied for UC in China [16]. There have been a large number of ICWM RCTs of UC published in Chinese and English literature. This study provides a scoping review of the reporting quality of the RCTs of ICWM for UC publications between January 1998 to June 2023. Unfortunately, the reporting quality we reviewed was suboptimal and substantial improvement could be required to meet the recommendations of reporting guidelines. Based on the CONSORT checklist, the items with good reporting in the included articles cover scientific background and rationale; settings and locations where the data were collected, intervention details; baseline demographic and clinical characteristics; and interpretation consistent with results. Although subgroup analysis showed better reporting quality after the CONSORT checklist was updated in 2010, this review demonstrated that there is much room for improvement with respect to ICWM RCTs reporting.

The inadequate reporting domains of reviewed articles include title and abstract, introduction, methods, results, discussion, and other information. With respect to the title and abstract, the reporting percentage for “identification as a randomized trial in the title” of the trials was only 0.89%. Most Chinese articles did not report this item. Indexers may not classify a report as an RCT if the authors do not explicitly report this information, therefore limiting the accessibility [21]. Contact details for the corresponding author were reported in 16.9% of trials. Inadequate contact information would restrict readers from contacting trialists for additional information or clarifications of reported data [22]. Among 1385 (95%) RCTs with abstract, the reporting quality was good in the description of the trial design, objective, number of participants randomized, and results in the section of “Abstract”. However, the overall quality of abstract reporting has been unsatisfactory, presenting the inadequate reporting of participants, interventions, outcomes, randomization, blinding, recruiting objects, outcomes, harms, trial registration, and funding. Regarding introduction, there was only 1/5 studies reported specific objectives or hypotheses. The majority of included trials had poor reporting in methods, including trial design, outcomes, sample size estimation, allocation concealment, implementation, blinding, and statistical methods. In the “Results” section, losses and exclusions after randomization, periods of recruitment and follow-up, precise outcomes, and adverse events were also poorly reported. Limitations, generalizability, registration, protocol, and funding reporting had much room for improvement. There are the major reasons why the total score of the CONSORT checklist was suboptimal. Similar problems were found in the previous study examining systematic reviews of ICWM [23].

Due to the specific characteristics of ICWM interventions, the ICWM-specific checklist was designed to assess the reporting features of ICWM trials, which mainly focused on the rationale, implementation and assessment, details of therapy combination, and efficacy assessment. According to the subgroup analysis, we found that the reporting quality in ICWM-specific items was enhanced after 2010. However, there were several problems in the ICWM reporting. First of all, the rationale or reason for choosing ICWM interventions was reported in less than 20% of studies. Secondly, the diagnostic criteria and outcome assessments of both CM and WM were rarely adopted simultaneously. Additionally, limited trials had reported specific situations (e.g., treatment points, stages of diseases, types of conditions) for the applicable scope of ICWM, settings, and locations where the trials were conducted, the background of treatment providers, measures to improve compliance, reasons for study design, interpretation of ICTM interventions, and conflicts of interests. There was also no study that provided information about participants exposed to ICWM treatment prior to recruitment and external validity of ICWM. Though 98.63% of studies concluded promising efficacy of ICWM for UC, the above problems not only limit the repeatability and reproducibility of the findings, hamper other researchers from identifying gaps that need to be addressed in the design and reporting of future studies, but also mislead healthcare providers in suggesting treatment decisions for patients. Given the deficiencies of reporting identified during this review, strengthening the reporting of the CONSORT are urgently needed. Besides, an extension for ICWM specifics of the CONSORT would be worth considering to improve the current situation.

In this review, we found several problems in the characteristics of included RCTs. Firstly, only 4.95% of articles were published in relatively high-quality journals, such as the Chinese Medical Core Journals and English journals with impact factors. These journals usually provide a peer review process, to assess the validity, quality, and often the originality of articles for publication [24]. Secondly, the most common design was a single center (97.94%) and sample size of 51–100 (70.3%), indicating high-quality ICWM interventional RCTs with multicenter design and larger sample size are needed. Thirdly, the intervention period of no more than 30 days was applied in half of the studies. However, whether such a short intervention period for UC was enough to obtain maximum effectiveness is unknown. Moreover, 82.37% of studies did not report the follow-up period, therefore the long-term effectiveness of ICWM is questionable [25]. Finally, more than two-thirds (71.88%) of the trials did not report the specific stages or phases of UC. TCM treatment strategies for inflammatory bowel diseases are based on disease gradation, stage, and segmentation from multi-dimensional treatment, including etiology, symptoms, syndromes, and internal and external treatment [26]. Chinese medical syndromes of active and remission stages of UC are significantly different [27]. Omit syndrome differentiation in Chinese medicine intervention may lead to unsuccessful treatment [28].

Our review has some limitations. First, this review identified full-text RCTs of UC with ICWM interventions published up to 16 June 2023 in the targeted databases. Any records which had not been included in these databases by that cut-off period, or without available full-text, as well as the grey literature have not been included. In addition, we included only articles in English and Chinese because of language limitations. As such, we may not have captured otherwise eligible trials published in other languages. Second, we rated the items with “not applicable” reporting as “2”, which might overoptimize the total scores. Furthermore, the CONSORT checklist is not intended to construct a “quality score”. Thus, using scores based on the reporting checklists might limit the accuracy of our evaluations and simplify the actual situation. Third, methodology quality (e.g., risk of bias) assessment was not conducted because this review only focused on the reporting quality.

## Conclusion

The reporting quality of ICWM RCTs for UC is suboptimal and substantial improvement is required to meet CONSORT guidelines. Consistent with previous studies [29–31], this review found improved reporting quality in reviewed RCTs after the CONSORT checklist was updated in 2010. We suggest that authors should adhere to the CONSORT 2010 statement in designing, conducting, and reporting RCTs and that researchers should consider developing a series of standard reporting items specifically relevant to ITCWM design, as an extension to the CONSORT. This might be an effective strategy for achieving the improvement needed [32].

### Supplementary Information

**Additional file 1: Appendix 1.** Search strategy. **Appendix 2.** Scoring rules for ICWM-specific items. **Appendix 3.** Types of CM complex intervention (based on Chinese herbal formulas). **Appendix 4.** Included studies (*n*=1,458) of this review.

## Data Availability

The data analyzed for this study are included in the manuscript and supplementary files.

## Abbreviations

CM: Chinese medicine
CONSORT: Consolidated Standards of Reporting Trials
ICWM: Integrated Chinese and Western medicine
IQR: Interquartile range
RCTs: Randomized clinical trials
UC: Ulcerative colitis
WM: Western medicine
