# Supplementary material for: Quality of reporting of integrative Chinese and Western medicine intervention in randomized controlled trials of ulcerative colitis: a review
Source: Syst Rev. 2023 Dec 8;12:228. doi: 10.1186/s13643-023-02402-2 (PMC10704853; doi:10.1186/s13643-023-02402-2)
Supplement: Supplementary file 1 — Additional file 1: Appendix 1. Search strategy. Appendix 2. Scoring rules for ICWM-specific items. Appendix 3. Types of CM complex intervention (based on Chinese herbal formulas). Appendix 4. Included studies (n=1,458) of this review. [file 13643_2023_2402_MOESM1_ESM.docx]

**Content**

[Appendix 1. Search strategy 2](#_Toc139556946)

[1. Databases: 2](#_Toc139556947)

[2. Search Strategy 2](#_Toc139556948)

[2.1 Search strategy for MEDLINE (via Ovid) 2](#_Toc139556949)

[2.2 Search strategy for Embase (via Ovid) 3](#_Toc139556950)

[2.3 Search strategy for Cochrane Central Register of Controlled Trials 5](#_Toc139556951)

[2.4 Search strategy for Web of science 5](#_Toc139556952)

[2.5 Search strategy for CINAHL 6](#_Toc139556953)

[2.6 Search strategy for Allied and Complementary Medicine (via Ovid) 7](#_Toc139556954)

[2.7 Search strategy for China National Knowledge Infrastructure 7](#_Toc139556955)

[2.8 Search strategy for Wanfang Data 8](#_Toc139556956)

[2.9 Search strategy for VIP Chinese Medical Journal Database 8](#_Toc139556957)

[2.10 Search strategy for Chinese Biomedical Literature Service System 9](#_Toc139556958)

[Appendix 2. Scoring rules for ICWM-specific items 10](#_Toc139556959)

[Appendix 3. Types of CM complex intervention (n=64) 21](#_Toc139556960)

[Appendix 4. Included studies (n=1,458) 22](#_Toc139556961)

# Appendix 1. Search strategy

## 1. Databases:

**MEDLINE** <1946 to 16 June 2023>, **Embase** <1974 to 16 June 2023>, **CENTRAL** (Cochrane Central Register of Controlled Trials) <May 2023>, **Web of science** <1900 to 16 June 2023>, **CINAHL** <1937 to 16 June 2023>, **AMED** (Allied and Complementary Medicine Database) <1985 to May 2023>, **CNKI** (China National Knowledge Infrastructure) <1979 to 16 June 2023>, **Wanfang** <1979 to 16 June 2023>, **VIP** (Chinese Science and Technology Periodical Database) <1989 to 16 June 2023>, **CBM** (Chinese Biomedical Literature Database) <1978 to 16 June 2023>.

## 2. Search Strategy

### 2.1 Search strategy for MEDLINE (via Ovid)

| 1 | exp ulcerative colitis/ or colitis.mp. |
| --- | --- |
| 2 | inflammatory bowel disease*.mp. |
| 3 | IBD.mp. |
| 4 | UC.mp. |
| 5 | (proctocolitis or proctitis or proctosigmoiditis or colorectitis or mucosal colitis or rectocolitis or rectosigmoiditis).mp. |
| 6 | or 1-5 |
| 7 | randomized controlled trial/ |
| 8 | controlled clinical trial/ |
| 9 | random$.tw. |
| 10 | factorial$.tw. |
| 11 | (crossover$ or cross over$ or cross-over$).tw. |
| 12 | placebo$.tw. |
| 13 | single blind.mp. |
| 14 | double blind.mp. |
| 15 | triple blind.mp. |
| 16 | (singl$ adj blind$).tw. |
| 17 | (double$ adj blind$).tw. |
| 18 | (tripl$ adj blind$).tw. |
| 19 | assign$.tw. |
| 20 | allocat$.tw. |
| 21 | or 7-20 |
| 22 | exp Drugs, Chinese Herbal/ or Chinese Herbal Drugs.mp. |
| 23 | exp Plants, Medicinal/ or Medicinal Plant$.mp. |
| 24 | exp Plant Extracts/ or Plant Extracts.mp. |
| 25 | exp Herbal Medicine/ or Herbal Medicine.mp. |
| 26 | exp Medicine, Chinese Traditional/ or Chinese Traditional Medicine.mp. |
| 27 | exp Medicine, Traditional/ or Traditional Medicine.mp. |
| 28 | exp Ethnobotany/ or Ethnobotany.mp. |
| 29 | exp Phytotherapy/ or Phytotherapy.mp. |
| 30 | exp Materia Medica/ or materia medica.mp. |
| 31 | chinese medicine$ or herb$.mp. |
| 32 | medicinal herb or pharmaceutical plant.mp. |
| 33 | Chinese medicine$ or traditional medicine$.mp. |
| 34 | herbal drug$ or herbal medicine$.mp. |
| 35 | medicinal plant$ or medicinal herb$.mp. |
| 36 | herb$ or herb$ formula$ or decoction$.mp. |
| 37 | herb$ granule$ or herb$ capsule$ or herb$ pellet$.mp. |
| 38 | (single#entity or single) adj3 (component or drug$ or herb$).mp. |
| 39 | compound prescription$ or herbal mixture or Fufang.mp. |
| 40 | Chinese Medicine Patent Prescription or proprietary Chinese medicines.mp. |
| 41 | Chinese patent adj3 (medicine or drug$).mp. |
| 42 | Chinese adj3 (patent or proprietary) adj3 (medicine or drug$).mp. |
| 43 | Chinese adj2 (patent or proprietary) adj2 (medicine or drug$ or prescription$).mp. |
| 44 | or 22-43 |
| 45 | 6 and 21 and 44 |
|  | N=315 |

### 2.2 Search strategy for Embase (via Ovid)

| 1 | exp ulcerative colitis/ or colitis.mp. |
| --- | --- |
| 2 | inflammatory bowel disease*.mp. |
| 3 | IBD.mp. |
| 4 | UC.mp. |
| 5 | (proctocolitis or proctitis or proctosigmoiditis or colorectitis or mucosal colitis or rectocolitis or rectosigmoiditis).mp. |
| 6 | or 1-5 |
| 7 | randomized controlled trial/ |
| 8 | controlled clinical trial/ |
| 9 | random$.tw. |
| 10 | factorial$.tw. |
| 11 | (crossover$ or cross over$ or cross-over$).tw. |
| 12 | placebo$.tw. |
| 13 | single blind.mp. |
| 14 | double blind.mp. |
| 15 | triple blind.mp. |
| 16 | (singl$ adj blind$).tw. |
| 17 | (double$ adj blind$).tw. |
| 18 | (tripl$ adj blind$).tw. |
| 19 | assign$.tw. |
| 20 | allocat$.tw. |
| 21 | or 7-20 |
| 22 | exp Drugs, Chinese Herbal/ or Chinese Herbal Drugs.mp. |
| 23 | exp Plants, Medicinal/ or Medicinal Plant$.mp. |
| 24 | exp Plant Extracts/ or Plant Extracts.mp. |
| 25 | exp Herbal Medicine/ or Herbal Medicine.mp. |
| 26 | exp Medicine, Chinese Traditional/ or Chinese Traditional Medicine.mp. |
| 27 | exp Medicine, Traditional/ or Traditional Medicine.mp. |
| 28 | exp Ethnobotany/ or Ethnobotany.mp. |
| 29 | exp Phytotherapy/ or Phytotherapy.mp. |
| 30 | exp Materia Medica/ or materia medica.mp. |
| 31 | chinese medicine$ or herb$.mp. |
| 32 | medicinal herb or pharmaceutical plant.mp. |
| 33 | Chinese medicine$ or traditional medicine$.mp. |
| 34 | herbal drug$ or herbal medicine$.mp. |
| 35 | medicinal plant$ or medicinal herb$.mp. |
| 36 | herb$ or herb$ formula$ or decoction$.mp. |
| 37 | herb$ granule$ or herb$ capsule$ or herb$ pellet$.mp. |
| 38 | (single#entity or single) adj3 (component or drug$ or herb$).mp. |
| 39 | compound prescription$ or herbal mixture or Fufang.mp. |
| 40 | Chinese Medicine Patent Prescription or proprietary Chinese medicines.mp. |
| 41 | Chinese patent adj3 (medicine or drug$).mp. |
| 42 | Chinese adj3 (patent or proprietary) adj3 (medicine or drug$).mp. |
| 43 | Chinese adj2 (patent or proprietary) adj2 (medicine or drug$ or prescription$).mp. |
| 44 | or 22-43 |
| 45 | 6 and 21 and 44 |
|  | N=1085 |

### 2.3 Search strategy for Cochrane Central Register of Controlled Trials

| 1 | MeSH descriptor: [Colitis, Ulcerative] explode all trees |
| --- | --- |
| 2 | Inflammatory bowel disease |
| 3 | IBD |
| 4 | UC |
| 5 | 1 or 2 or 3 or 4 |
| 6 | randomized controlled trial |
| 7 | controlled clinical trial |
| 8 | random* or factorial* or crossover* or placebo* or singl* blind* or double* blind* or trial* blind* or assign* or allocat* |
| 9 | #6 or #7 or #8 |
| 10 | MeSH descriptor: [Chinese Medicine, Traditional] explode all trees |
| 11 | MeSH descriptor: [Drugs, Chinese Herbal] explode all trees |
| 12 | MeSH descriptor: [Herbal Medicine] explode all trees |
| 13 | MeSH descriptor: [Traditional Medicine] explode all trees |
| 14 | MeSH descriptor: [Plant Extracts] explode all trees |
| 15 | MeSH descriptor: [Ethnobotany] explode all trees |
| 16 | MeSH descriptor: [Phytotherapy] explode all trees |
| 17 | MeSH descriptor: [Materia Medica] explode all trees |
| 18 | Chinese medicine* or Chinese herb* or medicinal herb* or medicinal Plant* or pharmaceutical plant* or Chinese medicine* or traditional medicine* or herbal drug* or herbal medicine* or medicinal plant* or medicinal herb* or herb* |
| 19 | single entity or compound prescription* or herbal mixture or Fufang or Chinese Medicine Patent Prescription or proprietary Chinese medicines or Chinese patent medicine* or Chinese patent drug* or Chinese proprietary medicine* or Chinese proprietary drug* or Chinese proprietary prescription* |
| 20 | #10 or #11 or #12 or #13 or #14 or #15 or #16 or #17 or #18 or #19 |
| 21 | #5 and #9 and #20 (Limit in Trials) |
|  | N=325 |

### 2.4 Search strategy for Web of science

| 1 | AB=(ulcerative colitis or colitis or inflammatory bowel disease or IBD or UC or proctocolitis or proctitis or proctosigmoiditis or colorectitis or mucosal colitis or rectocolitis or rectosigmoiditis) |
| --- | --- |
| 2 | TI=(ulcerative colitis or colitis or inflammatory bowel disease or IBD or UC or proctocolitis or proctitis or proctosigmoiditis or colorectitis or mucosal colitis or rectocolitis or rectosigmoiditis) |
| 3 | AB=(traditional Chinese medicine or TCM or Chinese Herbal Drugs or Medicinal Plant* or Plant Extracts or Herbal Medicine or Traditional Medicine or Chinese Medicine or Ethnobotany or Phytotherapy or Materia Medica or Chinese herb* or medicinal herb or pharmaceutical plant or herbal drug* or herbal medicine* or medicinal plant* or medicinal herb* or herb* or herb* formula* or decoction* or herb* granule* or herb* capsule* or herb* pellet* or single entity or compound prescription* or herbal mixture or Fufang or Chinese Medicine Patent Prescription or proprietary Chinese medicines or Chinese patent medicine or Chinese patent drug* or Chinese proprietary medicine* or Chinese proprietary drug* or Chinese proprietary prescription*) |
| 4 | TI=(traditional Chinese medicine or TCM or Chinese Herbal Drugs or Medicinal Plant* or Plant Extracts or Herbal Medicine or Traditional Medicine or Chinese Medicine or Ethnobotany or Phytotherapy or Materia Medica or Chinese herb* or medicinal herb or pharmaceutical plant or herbal drug* or herbal medicine* or medicinal plant* or medicinal herb* or herb* or herb* formula* or decoction* or herb* granule* or herb* capsule* or herb* pellet* or single entity or compound prescription* or herbal mixture or Fufang or Chinese Medicine Patent Prescription or proprietary Chinese medicines or Chinese patent medicine or Chinese patent drug* or Chinese proprietary medicine* or Chinese proprietary drug* or Chinese proprietary prescription*) |
| 5 | AB=(randomized controlled trial or controlled clinical trial or random* or factorial* or crossover* or placebo* or singl* blind* or double* blind* or tripl* blind* or assign* or allocat*) |
| 6 | TI=(randomized controlled trial or controlled clinical trial or random* or factorial* or crossover* or placebo* or singl* blind* or double* blind* or tripl* blind* or assign* or allocat*) |
| 7 | (1 OR 2) AND (3 OR 4) AND (5 OR 6) |
|  | N=235 |

### 2.5 Search strategy for CINAHL

| 1 | ulcerative colitis or colitis or inflammatory bowel disease or IBD or UC or proctocolitis or proctitis or proctosigmoiditis or colorectitis or mucosal colitis or rectocolitis or rectosigmoiditis |
| --- | --- |
| 2 | traditional Chinese medicine or TCM or Chinese Herbal Drugs or Medicinal Plant* or Plant Extracts or Herbal Medicine or Traditional Medicine or Chinese Medicine or Ethnobotany or Phytotherapy or Materia Medica or Chinese herb* or medicinal herb or pharmaceutical plant or herbal drug* or herbal medicine* or medicinal plant* or medicinal herb* or herb* or herb* formula* or decoction* or herb* granule* or herb* capsule* or herb* pellet* or single entity or compound prescription* or herbal mixture or Fufang or Chinese Medicine Patent Prescription or proprietary Chinese medicines or Chinese patent medicine or Chinese patent drug* or Chinese proprietary medicine* or Chinese proprietary drug* or Chinese proprietary prescription* |
| 3 | randomized controlled trial or controlled clinical trial or random* or factorial* or crossover* or placebo* or singl* blind* or double* blind* or tripl* blind* or assign* or allocat* |
| 4 | 1 AND 2 AND 3 |
|  | N=71 |

### 2.6 Search strategy for Allied and Complementary Medicine (via Ovid)

| 1 | (ulcerative colitis or colitis or inflammatory bowel disease or IBD or UC or proctocolitis or proctitis or proctosigmoiditis or colorectitis or mucosal colitis or rectocolitis or rectosigmoiditis).mp. |
| --- | --- |
| 2 | (traditional Chinese medicine or TCM or Chinese Herbal Drugs or Medicinal Plant* or Plant Extracts or Herbal Medicine or Traditional Medicine or Chinese Medicine or Ethnobotany or Phytotherapy or Materia Medica or Chinese herb* or medicinal herb or pharmaceutical plant or herbal drug* or herbal medicine* or medicinal plant* or medicinal herb* or herb* or herb* formula* or decoction* or herb* granule* or herb* capsule* or herb* pellet* or single entity or compound prescription* or herbal mixture or Fufang or Chinese Medicine Patent Prescription or proprietary Chinese medicines or Chinese patent medicine or Chinese patent drug* or Chinese proprietary medicine* or Chinese proprietary drug* or Chinese proprietary prescription*).mp. |
| 3 | (randomized controlled trial or controlled clinical trial or random* or factorial* or crossover* or placebo* or singl* blind* or double* blind* or tripl* blind* or assign* or allocat*).mp. |
| 4 | 1 AND 2 AND 3 |
|  | N=45 |

### 2.7 Search strategy for China National Knowledge Infrastructure

| 1 | SU=('溃疡性结肠炎'+'炎症性肠病'+'炎性肠疾病'+'非特异性结肠炎') AND SU=('中医'+'中医药'+'中华医药'+'草药'+'中草药'+'中药'+'中药材'+'复方'+'汤剂'+'方剂'+'中成药'+'成方制剂'+'注射剂 '+'加减方'+'外用中药'+'针灸'+'针刺'+'穴位'+'腧穴'+'经络'+'经脉'+'灸'+'推拿') AND SU=('中西医结合'+'中西结合'+'中西医联合'+'中西联合'+'中西医结合疗法'+'中西医结合试验'+'中西医结合治疗'+'中西医'+'中西'+'结合西药'+'联合西药'+'美沙拉嗪'+'柳氮磺吡啶'+'柳氮磺胺吡啶'+'激素'+'氨基水杨酸') AND SU=('临床试验'+'随机'+'对照'+'随机试验'+'随机对照试验'+'临床研究'+'半随机'+'临床观察 '+'疗效评价') |
| --- | --- |
|  | N=770 |

### 2.8 Search strategy for Wanfang Data

| 1 | (主题="溃疡性结肠炎" OR 主题="炎症性肠病" OR 主题="炎性肠疾病" OR 主题="非特异性结肠炎") AND (主题="中医药" OR 主题="草药" OR 主题="中药" OR 主题="复方" OR 主题="汤剂" OR 主题="方剂" OR 主题="中成药" OR 主题="注射剂" OR 主题="加减方" OR 主题="外用中药" OR 主题="针灸" OR 主题="针刺" OR 主题="穴位" OR 主题="腧穴" OR 主题="经络" OR 主题="经脉" OR 主题="灸" OR 主题="推拿") AND (主题="中西医结合" OR 主题="中西结合" OR 主题="中西医联合" OR 主题="中西联合" OR 主题="中西医结合疗法" OR 主题="中西医结合试验" OR 主题="中西医结合治疗" OR 主题="中西医" OR 主题="中西" OR 主题="结合西药" OR 主题="联合西药" OR 主题="美沙拉嗪" OR 主题="柳氮磺吡啶" OR 主题="柳氮磺胺吡啶" OR 主题="激素" OR 主题="氨基水杨酸") AND (主题="临床试验" OR 主题="随机" OR 主题="对照" OR 主题="随机试验" OR 主题="随机对照试验" OR 主题="临床研究" OR 主题="半随机" OR 主题="临床观察" OR 主题="疗效评价") |
| --- | --- |
|  | N=2188 |

### 2.9 Search strategy for VIP Chinese Medical Journal Database

| 1 | ((M="溃疡性结肠炎" OR M="炎症性肠病" OR M="炎性肠疾病" OR M="非特异性结肠炎") AND (M="中医药" OR M="草药" OR M="中药" OR M="复方" OR M="汤剂" OR M="方剂" OR M="中成药" OR M="注射剂" OR M="加减方" OR M="外用中药" OR M="针灸" OR M="针刺" OR M="穴位" OR M="腧穴" OR M="经络" OR M="经脉" OR M="灸" OR M="推拿") AND (M="中西医结合" OR M="中西结合" OR M="中西医联合" OR M="中西联合" OR M="中西医结合疗法" OR M="中西医结合试验" OR M="中西医结合治疗" OR M="中西医" OR M="中西" OR M="结合西药" OR M="联合西药" OR M="美沙拉嗪" OR M="柳氮磺吡啶" OR M="柳氮磺胺吡啶" OR M="激素" OR M="氨基水杨酸") AND (M="临床试验" OR M="随机" OR M="对照" OR M="随机试验" OR M="随机对照试验" OR M="临床研究" OR M="半随机" OR M="临床观察" OR M="疗效评价")) |
| --- | --- |
|  | N=175 |

### 2.10 Search strategy for Chinese Biomedical Literature Service System

| 1 | (溃疡性结肠炎 or 炎症性肠病 or 炎性肠疾病 or 非特异性结肠炎) AND (中医药 or 草药 or 中药 or 复方 or 汤剂 or 方剂 or 中成药 or 注射剂 or 加减方 or 外用中药 or 针灸 or 针刺 or 穴位 or 腧穴 or 经络 or 经脉 or 灸 or 推拿) AND (中西医结合 or 中西结合 or 中西医联合 or 中西联合 or 中西医结合疗法 or 中西医结合试验 or 中西医结合治疗 or 中西医 or 中西 or 结合西药 or 联合西药 or 美沙拉嗪 or 柳氮磺吡啶 or 柳氮磺胺吡啶 or 激素 or 氨基水杨酸) AND (临床试验 or 随机 or 对照 or 随机试验 or 随机对照试验 or 临床研究 or 半随机 or 临床观察 or 疗效评价) |
| --- | --- |
|  | N=4123 |

# Appendix 2. Scoring rules for ICWM-specific items

| **Section** | **Q. No** | **Specifics** | **Definition of Full report (scored as 2), Partial report (scored as 1),**  **Not report (scored as 0) and**  **Not Applicable (did not be calculated)** | **Examples** |
| --- | --- | --- | --- | --- |
| Title | Q1 | Whether the feature of ICWM was presented in the section of "Title" (e.g., generalized term of ICWM, or specific CM and WM interventions provided in the title)? | “Full report” was considered if the reader can determine from the title that the study was ICWM design. Generally, it was presented as follows: 1) the word “ICWM” was used; 2) it referred to CM interventions combined with WM interventions in the title. “Not report” was considered if title could not be identified as ICWM design. | 1. Full report: “Observation on therapeutic effect of sulfasalazine combined with traditional Chinese medicine retention enema on ulcerative colitis.” 2. Full report: “56 Cases of Ulcerative Colitis of Rectum and Sigmoid Colon Treated with Kuisukang Decoction and Mesalazine.” |
| Abstract | Q2 | Whether the eligibility criteria of participants included both Chinese and western medical diagnosis in Methods of Abstract? | “Full report” was considered if both traditional Chinese medical names (or pattern name) and Western of UC were reported in Method of Abstract.  “Partial report” was considered if there was only Western medical name or Chinese medical name (or pattern name) of UC. | 1. Full report: “Abstract: 320 active UC patients with TCM pattern of damp-heat accumulating in the interior were assigned to two groups.” 2. Partial report: “Abstract: To explore the effect of Jianpi Huashi Decoction combined with Qingchang Suppository on the quality of life of patients with ulcerative colitis of spleen deficiency and damp heat type.” |
|  | Q3 | Whether the study objectives or hypotheses were focused on the ICWM interventions in Abstract? | “Full report” was considered if the reader can identify the study objectives or hypotheses were focused on the “ICWM” interventions.  “Not report” was considered if title could not be identified as ICWM design. | 1. Full report: “Abstract: To observe the clinical efficacy and safety of modified Shaoyao decoction combined with rectal ozone perfusion in the treatment of ulcerative colitis with colon damp heat syndrome.” 2. Full report: “Abstract: To study the clinical efficacy of modified Baitouweng decoction and mesalazine in the treatment of damp heat ulcerative colitis.” |
|  | Q4 | Whether the outcome measures included both CM and WM related endpoints in Abstract? | “Full report” was considered if the assessed outcomes included both CM and WM related indicators were reported in the “Abstract-methods”.  “Partial report” was considered if there was only WM or CM related indicators in the “Abstract-methods”.  “Not report” was considered if there were no indicators for assessing outcomes were reported in the “Abstract-methods”. | 1. Full report: “Abstract: After one course of treatment, evaluate the efficacy of TCM syndromes and mucosal lesions, and observe the changes of Sutherland disease activity index scores before and after treatment.” 2. Partial report: “Abstract: Observe the improvement of clinical symptoms, intestinal mucosa and recurrence rate of the two groups.” 3. Not report: “Abstract: Compare the efficacy of the two groups.” |
|  | Q5 | Whether the effect of studied ICWM interventions was reported in Conclusion of Abstract? | “Full report” was considered if the feature of ICWM was presented in “Conclusion of Abstract “, including the word “ICWM” or the specific interventions name of combined CM and WM.  “Not report” was considered if there was no relevant description. | 1. Full report: “Abstract: Conclusion：The combination of traditional Chinese and western medicine has advantages in treating ulcerative colitis.” 2. Full report: “Abstract: Conclusion: The combination of traditional Chinese medicine and western medicine has a good effect on UC.” |
| Key words | Q6 | Whether the feature or design of ICWM study were reflected in Keywords? | “Full report” was considered if Keywords included the word “ICWM” or the CM and WM intervention.  “Partial report” was considered if Keywords only included CM or WM interventions.  “Not report” was considered if there were not Keywords or any interventions in the Keywords. | 1. Full report: “Key words: Chronic ulcerative colitis; Enema therapy; Sulfasalazine; Integrated Chinese and Western Medicine” 2. Partial report: “Key words: Ulcerative colitis; Mesalazine, retention enema; Inflammatory factor level; immunity; Recurrence rate” 3. Not report: “Key words: Ulcerative colitis; treatment” |
| Introduction | Q7 | Whether the reason/rationale about ICWM intervention for the study design was reported in Background? | “Full report” was considered if the current condition and problems of CM and WM in the treatment of UC were reported in “Introduction”.  “Partial report” was considered if the article only reported the condition of CM or WM in the treatment of UC in “Introduction”.  “Not report” was considered if there was not any description of current status and problems of CM or WM in the treatment of UC in “Introduction”. | 1. Full report: “At present, the therapeutic drugs for UC are mainly salicylic acid, adrenocortical hormones, immunosuppressants, etc. Most of them are prone to relapse after drug withdrawal, with many long-term side effects, and some stubborn cases have poor efficacy. In recent years, Chinese medicine has achieved satisfactory results in the treatment of UC, and has the advantages of small side effects, low recurrence rate and good long-term effect.” 2. Partial report: “Salicylic acid, corticosteroids, immunosuppressants and other drugs are often used in western medicine, but the clinical cure rate is low and it is easy to relapse.” 3. Not report: “The author treated 204 cases of ulcerative colitis from 2002 to 2009. 119 cases were treated with integrated traditional Chinese and western medicine, and 86 cases were treated with western medicine alone as the control group. The curative effect was satisfactory, as reported below.” |
|  | Q8 | Whether any necessity/advantage about ICWM intervention was reported in Background? | “Full report” was considered if the necessity or advantage about ICWM intervention was reported in “Introduction”.  “Not report” was considered if there was not any description of necessity or advantage about ICWM intervention in “Introduction” | 1. Full report: “In recent years, with the continuous development of traditional Chinese medicine dosage forms, the combination of traditional Chinese and western medicine has made breakthrough progress in the treatment of ulcerative colitis, and has the advantages of strong pertinence, diverse treatment methods, small side effects, and accurate efficacy.” 2. Full report: “For many years, the anorectal department of our hospital, together with the Department of Gastroenterology and the Endoscope Center, has used the combination of traditional Chinese and western medicine to treat this disease, especially the local enema treatment with Chinese medicine for clearing heat and activating blood circulation, which has significantly improved the clinical effect.” |
|  | Q9 | Whether the objectives or hypotheses were focused on the ICWM interventions in the Background (e.g., improve the efficacy/safety, or reduce the side-effects)? | “Full report” was considered if the readers can determine the study objectives or hypotheses were focused on the “ICWM” interventions in “Introduction”.  “Not report” was considered if there were not any descriptions of ICWM in “Introduction”. | 1. Full report: “To explore the clinical efficacy of Changyankang combined with mesalazine in the treatment of ulcerative colitis.” 2. Full report: “The purpose of this study was to observe the effect of Changchangling and 5-ASA on UC.” |
| Methods |  |  |  |  |
| Participants | Q10 | Whether the eligibility criteria of participants included both Chinese and western medical diagnosis in Methods? | “Full report” was considered if the eligibility criteria of participants included both Chinese and western medical diagnosis and provided references.  “Partial report” was considered if the eligibility criteria of participants included both Chinese and western medical diagnostic criteria, but no references.  “Not applicable” was considered if there was only Chinese or western medical diagnosis in the eligibility criteria of participants. | 1. Full report: “Diagnostic criteria of western medicine: According to the consensus opinion on the diagnostic and therapeutic norms of inflammatory bowel disease in China issued by the inflammatory bowel disease working group of Digestive Branch of Chinese Medical Association in 2007; The syndrome differentiation standard of traditional Chinese medicine refers to the syndrome diagnosis standard of "Guiding Principles for Clinical Research of New Chinese Medicines" for the treatment of chronic non-specific ulcerative colitis, which conforms to the syndrome differentiation of traditional Chinese medicine with blood stasis syndrome.” 2. Partial report: “A patient with mild to moderate ulcerative colitis at the initial stage of activity admitted to our hospital. It is in line with the diagnostic standard of the Consensus on the Diagnosis and Treatment of Ulcerative Colitis issued by the Spleen and Stomach Disease Branch of the Chinese Society of Traditional Chinese Medicine in 2010, and the TCM syndrome differentiation is the damp heat type of large intestine.” 3. Not applicable: “According to the national diagnostic criteria for chronic pulmonary infection and intestinal diseases, the patient's condition is judged, and patients with other intestinal diseases are excluded.” |
|  | Q11 | Whether the specific information of disease (e.g., classification of disease, treatment points, stages of diseases) of the ICWM was reported in Methods? | “Full report” was considered if the classification of UC was reported in “Methods”.  “Not report” was considered if there was only “UC” without any classification. | 1. Full report: “Inclusion criteria: meet the diagnostic criteria formulated by the Xiaoxue Branch of the Chinese Medical Association; The diagnosis was confirmed by routine stool examination, electronic colonoscopy and pathological biopsy; In UC activity period; Aged 18-65. Exclusion criteria: UC remission period.” 2. Full report: “112 patients with active ulcerative colitis in our hospital from January 2010 to June 2013 were selected, all of whom met the diagnostic and therapeutic criteria for inflammatory bowel disease formulated by the Digestive Branch of the Chinese Medical Association.” |
|  | Q12 | Whether any specific criteria related ICWM in the selection of study centers? | “Full report” was considered if there was ICWM-related study center in “Methods”.  “Not report” was considered if there was not ICWM-related study center in “Methods”. | 1. Full report: “All cases were from the outpatient and hospitalized UC patients of mild to moderate active stage in Nanjing Integrated Traditional Chinese and Western Medicine Hospital and the Third Affiliated Hospital of Nanjing University of Traditional Chinese Medicine.” 2. Full report: “76 patients with ulcerative colitis were selected as the study subjects. These patients were all admitted to Chengdu Integrated Traditional Chinese and Western Medicine Hospital from January 2016 to January 2018.” |
| Intervention | Q13 | Whether the specific type/way of integration of CM and WM interventions (such as overlying, one-after-another, or add-on design) was reported in Methods? | “Full report” was considered if the specific type/way of the combination of CM and WM was reported. Generally, the ways included overlying, one-after-another, or add-on design.  “Not report” was considered if there was not any description of specific way. | 1. Full report: “The treatment group was treated with Meizhu Kuijie enema soup and mesalazine.” 2. Full report: “Both groups were treated by supplementing intestinal beneficial bacteria and mesalazine sustained-release agent orally. If the stool purulent cells were more than normal, they were given short-term anti infection treatment according to the results of stool culture drug sensitivity. The treatment group added Kuijie I.” |
|  | Q14 | In the ICWM group, whether CM intervention(s) was reported with sufficient details to allow replication, including how and when they were administered? | “Full report” was considered if sufficient details of CM interventions in the ICWM-design group were reported. Generally, the details include name, dosage, route of administration, frequency of intervention, and therapeutic period. If the CM intervention is a formula decoction, it ought to report the name and dosage of every herb included in the formula. If the CM intervention is acupuncture or other external therapy, it should report the acupoint(s) or any details.  “Not report” was considered if there was insufficient information about CM intervention of ICWM group, which reader could not repeat the trial followed the direction. | 1. Full report: “Pulsatilla, Cortex Fraxini, Fried Atractylodes Macrocephalae, Radix Paeoniae Alba, Radix Saposhnikoviae, Stewed Radix Puerariae, Stewed Radix Scutellariae, Stewed Muxiang, and Fried Fructus Aurantii, 10g each, 30g Dijincao, 3g each, Rhizoma Curcumae Curcumae and Glycyrrhizae. Increase or decrease with symptoms: pyemia will increase 10g of Sanguisorba officinalis and Sophora japonica respectively; Add 5g rhubarb to the instant weight; For those with cold dampness, add 5g of ginger, 10g of agastache and perilla leaves respectively, and subtract scutellaria baicalensis, coptis chinensis, etc. 1 dose per day, decoction twice, 15 days as a course of treatment, can be used for 2 to 3 courses.” 2. Full report: “On the basis of oral sulfasalazine, the treatment group used 2.0g sulfasalazine, 10g tin powder, 50ml 0.9% sodium chloride solution, and mixed well for retention enema.” |
|  | Q15 | In the ICWM group, whether WM intervention(s) was reported with sufficient details to allow replication, including how and when they were administered? | “Full report” was considered if sufficient details of WM interventions in the ICWM group were reported. Generally, the details include name, dosage, route of administration, frequency of intervention, and therapeutic period.  “Not report” was considered if there was insufficient information about WM intervention of ICWM group, which reader could not repeat the trial followed the direction. | 1. Full report: “The control group was given sulfasalazine orally, 1.5 g/time, 4 times/day. After symptom relief, it was changed to 1g times per time, 3 times per day. After 4 weeks, it was changed to 0.5g each time, 3 times for 1 day, and the drug was administered for 2 weeks. The treatment group was given the above treatment and Chinese medicine retention enema at the same time.” 2. Full report: “The control group was given sulfasalazine orally, 1.5 g/time, 4 times/day. After symptom relief, it was changed to 1g times per time, 3 times per day. After 4 weeks, it was changed to 0.5g each time, 3 times for 1 day, and the drug was administered for 2 weeks. The treatment group was given the above treatment and Chinese medicine retention enema at the same time.” |
|  | Q16 | Whether the rationale for the choice of the control group(s) was provided? | “Full report” was considered if there was the rationale for the choice of the control group(s) in “Methods”.  “Not report” was considered if there was not the rationale for the choice of the control group(s) in “Methods”. | 1. Full report: “The condition of patients in the control group was similar to that in the experimental group, and the baseline data was not statistically significant and comparable.” |
|  | Q17 | In the control group, whether sufficient details were reported to allow replication? | “Full report” was considered if sufficient details of interventions in the controlled group were reported, the requirement was as the same as ICWM group.  “Not report” was considered if there was insufficient information that reader could not repeat the trial followed the direction. | 1. Full report: “The western medicine sulfasalazine was given orally for 4 days, 1g each time. After 4 weeks of administration, it was changed to 1g each time, twice a day, and then used for 4 weeks.” 2. Full report: “Control group: Take 0.2 g of norfloxacin capsule, 0.1 g of vitamin C, 10 mg of vitamin B, 0.4 g of berberine hydrochloride, and 5 mg of nisone acetate tablets orally, all three times a day.” |
|  | Q18 | Whether any description of treatment providers’ background (e.g., qualification and/or experiences in ICWM, or whether the providers conducted CM and WM separately)? | “Full report” was considered if there was any description of treatment providers’ background.  “Not report” was considered if there was not descriptions of treatment providers’ background. | 1. Full report: “Acupuncture treatment is performed by the Acupuncture and Moxibustion Department of our hospital.” 2. Full report: “The above acupuncture was completed by the Acupuncture and Moxibustion Room of the Rehabilitation Department of Traditional Chinese Medicine of our hospital, and the points were added or subtracted according to different conditions.” |
|  | Q19 | Whether any measures were adopted to evaluate or improve the compliance of participants? | “Full report” was considered if there were any measures were adopted to evaluate or improve the compliance of participants.  “Not report” was considered if there were not measures were adopted to evaluate or improve the compliance of participants. | 1. Full report: “To improve patient compliance: 1. The questionnaire survey method was used to monitor the compliance of subjects with oral drugs. At the same time, in order to ensure uniform operation methods and clear compliance of patients, the retention enema was uniformly operated by nurses specially trained by the hospital. 2 The retention enema bottle is also packaged with black opaque paper to avoid the patient knowing the medicine used. 3 Explain the treatment plan and the significance and purpose of this experiment to the patient in detail, write clearly the medication method on the drugs provided, explain clearly the time of return visit, and use telephone notification to ensure that the return visit is carried out smoothly on schedule. 4 Provide patients with reduced or exempted registration fees, health education or disease assessment with more medical care knowledge, provide fare for patients with financial difficulties, improve service attitude, and establish contact cards for doctor-patient relationship.” |
| Outcome | Q20 | Whether the outcome measures included both CM and WM related endpoints in Methods? | “Full report” was considered if the assessed outcomes included both CM and WM related indicators were reported.  “Not report” was considered if there was only CM or WM outcome indicators. | 1. Full report: “Criteria for efficacy determination: clinical cure: main clinical symptoms (diarrhea, mucopurulent bloody stool, abdominal pain) disappear, tongue and pulse conditions return to normal, mucosa lesions return to normal after colonoscopy, or ulcer lesions have formed scar, and stool is normal after routine microscopy for 3 times. Remarkable effect: the main clinical symptoms have basically disappeared, the tongue and pulse conditions have basically returned to normal, the degree of recovery of mucosal lesions by colonoscopy is above grade II (3++), and the number of red blood cells and white blood cells in stool routine examination is less than 3 per high magnification field of vision; Improvement: the main clinical symptoms were moderately improved by more than Grade II, the recovery of mucosal lesions by colonoscopy was more than Grade I, and the number of red blood cells and white blood cells in stool routine examination was about 5; Ineffective: cases that fail to meet the above effective standards.” 2. Full report: “Electronic colonoscopy was performed before and after treatment to observe intestinal mucosal lesions; Observe the curative effect of TCM syndromes and the changes of main symptom scores.” |
| Blinding | Q21 | For the studies with open label, whether any reasons or explanations for such design was reported? | “Full report” was considered if there was any explanation for such design was conducted or the study was blinding design.  “Not report” was considered if there was no explanation of the design. | 1. Full report: “Based on the inherent characteristics of acupuncture clinical research, this study adopts the single blind method, that is, the trial researchers and clinical operators are separated, the spirit of blind method is implemented, blind method is adopted for evaluation, and the efficacy is evaluated by a third party who does not know the grouping situation; The data were summarized by blind statistical analysis, and the researchers, operators and statisticians were separated.” 2. Full report: “Because the number of patients is small, the trial drug is complex, and it is difficult to implement blind method, so this study is an open clinical trial.” |
| Control | Q22 | In the control group(s), did the placebo of WM invention(s) was included? If so, whether sufficient details were provided? | “Full report” was considered if the study conducted the WM placebo.  “Not report” was considered if the trial conducted the placebo design but there was not WM placebo.  “Not applicable” was considered if the trial was not placebo design. | 1. Full report: “Test group: Hudi enteric coated capsule, 4 capsules once, 3 times a day; Mesalazine enteric coated tablets simulator (composition: dextrin and starch, taste and appearance are the same as those of mesalazine enteric coated tablets), 4 tablets once, 3 times a day.” 2. Not report: “The basic treatment was to take orally mesalazine enteric coated tablets (Sunflower Pharmaceutical Group Jiamusi Luling Pharmaceutical Co., Ltd., National Drug Approval No. H19980148), 2g/time, twice a day, for two consecutive weeks. If the symptoms were not well controlled (Mayo score decreased by less than 30% from the baseline or fecal blood score decreased by less than 1 point), they were changed to prednisone acetate tablets (Furen Pharmaceutical Group Co., Ltd., National Drug Approval No. H20074062), 0.75 mg kg · d, three times orally. In case of remission, maintain treatment with mesalazine enteric coated tablets, 0.5 g/time, 3 times/d The control group was given a simulated drug of colon heat clearing pill (made of starch, pigment and flavoring in the preparation room of Henan Traditional Chinese Medicine Hospital, with the same smell, color and packaging as the colon heat clearing pill), 10 g/time, 3 times/day, before meals. The course of treatment of both groups was 12 weeks, and 3 months of follow-up was carried out.” 3. Not applicable: “The control group received symptomatic treatment such as adjusting the balance of water and electrolyte, and took sulfasalazine enteric coated tablets (Shanghai Fuda Pharmaceutical Co., Ltd., National Drug Approval No. H31020840, specification: 0.25 g/tablet) 0.5 g/time, 4 times/day. After no obvious discomfort, the dosage could be increased to 1.0 g/time, 4 times/day, and gradually reduced to the maintenance dosage after symptom relief, 0.5 g/time, 3 times/day.” |
|  | Q23 | In the control group(s), did the placebo of CM invention(s) was included? If so, whether sufficient details were provided? | “Full report” was considered if the study conducted the CM placebo.  “Not report” was considered if the trial conducted the placebo design but there was not CM placebo.  “Not applicable” was considered if the trial was not placebo design. | 1. Full report: “The treatment group was treated with integrated traditional Chinese and western medicine. (1) Mesalazine enteric coated tablets (produced by Sunflower Pharmaceutical Group Jiamusi Luling Pharmaceutical Co., Ltd., specification: 0.25g/capsule, batch number: National Drug Approval No. H19980148) are taken orally, 1g each time, three times a day; (2) The self-made traditional Chinese medicine enema prescription is: Patrinia villosa 10g, Coptis chinensis 10g, Portulaca oleracea 10g, Pulsatilla chinensis 10g, Phellodendron chinense 10g, Sanguisorba officinalis charcoal 10g, Agrimonia pilosa 10g, Radix Angelicae alba 10g, and Galla chinensis 10g. The decoction is uniformly decocted into 100ml traditional Chinese medicine decoction by the hospital's pharmacy, and the patient is allowed to empty the stool before sleep and then retain the enema for more than 30min. The control group was treated with conventional western medicine. (1) The usage of mesalazine enteric coated tablets was the same as that of the treatment group; (2) Placebo (100mL normal saline packed by the pharmacy of the hospital) was given for enema, and the usage was the same as that of the treatment group.” 2. Not report: “The control group took sulfasalazine tablets placebo, 4g each time, 3 times a day; Take orally the self-made Jianpi Shugan decoction (fried atractylodes macrocephala 30g, poria 15g, fried coix seed 10g, fried tangerine peel 20g, bupleurum chinense 12g, fructus aurantii 15g, green peel 10g, vanilla 10g, white peony 30g, black plum 6g, fangfeng 20g, licorice 6g, jujube 3 pieces), 200ml each time, twice a day. The course of treatment is 10 weeks.” 3. Not applicable: “The control group was treated with sodium aminosalicylate enteric coated tablets (Huazhong Pharmaceutical Co., Ltd., GYZZ H42021217), orally, 1g times, 3 times a day. On this basis, the observation group was additionally treated with traditional Chinese medicine for tonifying the spleen and stomach. The prescription was as follows: 15g Dangshen, 12g Poria cocos, 12g Coix seed, 9g Atractylodes macrocephala, Platycodon grandiflorum, and Chinese yam, 6g Glycyrrhiza uralensis, 3g Amomum villosum. They were decocted in water, 300mL of juice was taken, taken warm twice in the morning and evening, 1 dose per dose. Both groups were treated continuously for 3 months. Both groups of patients should avoid eating cold, spicy and other stimulating foods before treatment.” |
| Results |  |  |  |  |
| Baseline | Q24 | In the section of Results, whether any information about the participants exposed to ICWM treatment prior to recruitment was mentioned in the baseline data? | “Full report” was considered if the information about the participants exposed to ICWM treatment prior to recruitment (restrict to ICWM interventions).  “Not report” was considered if there was not any description about previous treatment of ICWM. | 1. Full report: “All patients have not received TCM treatment before.” |
| Discussion |  |  |  |  |
|  | Q25 | Whether any discussion about external validity of ICWM results was reported, particular in different environments? | “Full report” was considered if there was any discussion about external validity of ICWM results was reported.  “Not report” was considered if there was not any discussion about external validity of ICWM results was reported. | 1. Full report: “In this study, the two clinical interveners in charge of acupuncture treatment were all famous TCM doctors at or above the provincial level specializing in acupuncture treatment. They had relatively rich clinical experience, so they may have an objective impact on the efficacy.” |
|  | Q26 | Whether interpretation and significance of studied ICWM interventions for the disease was reported in Discussion? | “Full report” was considered if the article respectively reported the advantages and disadvantages of CM and WM intervention(s) and manifested that ICWM works better.  “Partial report” was considered if the article only reported the efficacy of CM or WM intervention(s), or it only described “ICWM works better” without further description.  “Not report” was considered if there was not any description about ICWM interventions. | 1. Full report: “The traditional Chinese medicine enema can bypass the liver and enter the large circulation from the rectum, avoiding the first pass elimination effect of oral administration, and can reduce the gastrointestinal irritation; The enema makes the medicine reach the disease place directly, improves the local drug concentration, and the Chinese medicine has definite curative effect, small side effects, and is easy for patients to accept. At the same time, it is taken orally in combination with the western medicine SASP. This product is a sulfa antibiotic, which is not easy to be absorbed by oral administration. The absorbed part is decomposed into 5-aminosalicylic acid and sulfapyridine under the action of intestinal microorganisms. 5-aminosalicylic acid stays in the intestinal wall tissue for a long time after complexing with the intestinal wall connective tissue to play an antibacterial, anti-inflammatory and immunosuppressive role, such as reducing Escherichia coli and Clostridium, At the same time, it can inhibit the synthesis of prostaglandins and other inflammatory mediators leukotriene to achieve therapeutic purposes.” 2. Partial report: “The combination of traditional Chinese medicine retention enema and Aidisa oral administration can significantly improve the clinical symptoms of dogs, especially the highest clinical complete remission rate when the lesions are limited to the rectum and sigmoid colon, giving full play to the advantage of retention enema to reach the hospital directly, and improving the efficacy.” 3. Not report: “Through this clinical control study, the effect of oral mesalazine combined with traditional Chinese medicine enema in the treatment of mild and moderate ulcerative colitis is positive, especially for ulcerative rectitis.” |
| Other information |  |  |  |  |
| Interests | Q27 | Whether any potential conflicts of interests were clearly reported? | “Full report” was considered if the article provides sufficient details about the conflicts of interests; or it declared no interest.  “Partial report” was considered if the article did not refer to the potential conflicts of interests, but it reported that the formula is made by experts of hospital where conducted the RCT trial.  “Not report” was considered if there was not mentioned about conflicts of interests in the article. | 1. Full report: “Conflict of interest: All authors declare that there is no conflict of interest.” 2. Partial report: “The Sanhuang enema liquid developed by our hospital is mainly composed of rhubarb, scutellaria baicalensis and phellodendron chinense, which has the effects of clearing heat, drying dampness, detoxification, anti-inflammatory, spasmolysis and hemostasis. It has good long-term local application effect.” 3. Not report: “After oral administration, sulfasalazine colon enteric coated capsules release drugs after reaching the colon through the stomach and small intestine, thus prolonging the local retention time of drugs in the colon. Drugs can be widely distributed in the cecum, ascending and descending intestines, transverse colon and sigmoid colon. It can effectively improve the therapeutic effect, and at the same time, it is convenient to use, so that the adverse reactions of gastrointestinal tract can be significantly reduced, which has a significant therapeutic effect on ulcerative colitis.” |

*Integrated Chinese and Western Medicine (ICWM); Chinese Medicine (CM); Western Medicine (WM)*

**Appendix 3. Types of CM complex intervention (n=64)**

| **Types** | **N (%)** |
| --- | --- |
| Chinese Compound formulas combined with acupuncture | 21 (32.81) |
| Chinese Compound formulas combined with moxibustion | 18 (28.13) |
| Chinese Compound formulas combined with ear points therapy | 5 (7.81) |
| Chinese Compound formulas combined with acupuncture and moxibustion | 3 (4.69) |
| Chinese Compound formulas combined with Point injection | 2 (3.13) |
| Chinese Compound formulas combined with hot ironing therapy | 2 (3.13) |
| Chinese Compound formulas combined with acupuncture, moxibustion and diet therapy | 1 (1.56) |
| Chinese Compound formulas combined with Chinese single herbal medicine | 1 (1.56) |
| Chinese Compound formulas combined with acupoint massage | 1 (1.56) |
| Chinese Compound formulas combined with Five notes therapy | 1 (1.56) |
| Chinese Compound formulas combined with Catgut-embedding therapy | 1 (1.56) |
| Chinese Compound formulas combined with Thermal Design Power (TDP) light therapy | 1 (1.56) |
| Acupuncture combined with moxibustion | 4 (6.25) |
| Moxibustion combined with Five notes therapy | 2 (3.13) |
| Single herbs combined with moxibustion | 1 (1.56) |

# Appendix 4. Included studies (n=1,458)

1. 乐毅.中西医结合治疗溃疡性结肠炎临床观察[J].中医药学报,2010,38(01):87-88.
2. 伍运生,熊国强,陈出新.柳氮磺吡啶联合中药保留灌肠治疗溃疡性结肠炎的疗效观察[J].新医学,2010,41(08):538-540.
3. 刘凌云,杨伟峰,丁纪明.联用疏肝理脾片和柳氮磺吡啶治疗溃疡性结肠炎的临床观察[J].实用预防医学,2010,17(11):2254-2255.
4. 刘红.水针疗法配合保留灌肠治疗溃疡性结肠炎45例[J].中西医结合研究,2010,2(04):198.
5. 刘红,许雷.中西医结合治疗溃疡性结肠炎的相关性研究[J].中国医药导报,2010,7(26):68-69.
6. 刘伟华.中西医结合灌肠治疗结肠炎临床疗效观察[J].航空航天医药,2010,21(05):818.
7. 卜辉. 中西医结合治疗溃疡性结肠炎31例[J]. 实用中医内科杂志,2010,24(5):76-77.
8. 叶武,刘鹏飞. 锡类散保留灌肠治疗溃疡性结肠炎30例[J]. 陕西中医,2010,31(9):1141-1142.
9. 向晓玲. 中药保留灌肠治疗溃疡性结肠炎效果观察[J]. 现代医药卫生,2010,26(14):2109-2111.
10. 唐付才,查文俊,王莹,等. 中药内服结合保留灌肠治疗活动期溃疡性结肠炎临床观察[J]. 中国中医药信息杂志,2010,17(12):67-68.
11. 商月娥,冯中贤,王建一. 中药保留灌肠治疗非特异性溃疡性结肠炎60例[J]. 中国中医急症,2010,19(6):1034-1035.
12. 孙宝民. 探讨中西医结合治疗溃疡性结肠炎的临床疗效[J]. 中国现代药物应用,2010,4(2):151-152.
13. 孙阳. 中药灌肠联合美沙拉嗪治疗溃疡性结肠炎临床疗效及对TNF-α的影响[D]. 黑龙江:黑龙江中医药大学,2010.
14. 宋素华,孟淑红,关慧娟. 中西医结合治疗溃疡性结肠炎疗效观察和机制探讨[J]. 四川中医,2010,28(4):66-68.
15. 崔静. 针刺治疗对溃疡性结肠炎患者细胞因子的影响[J]. 河北医药,2010,32(11):1409-1410.
16. 常东,刘子志,李键,等. 中药溃结灵配合柳氮磺胺吡啶对溃疡性结肠炎患者防御素、一氧化氮及丙二醛表达的影响[J]. 中国中医急症,2010,19(10):1654-1655,1673.
17. 张勇,耿云涛. 中药保留灌肠在溃疡性结肠炎治疗中的应用[J]. 中国中医药现代远程教育,2010,8(24):163-163. DOI:10.3969/j.issn.1672-2779.2010.24.138.
18. 张国旗. 中西医结合治疗溃疡性结肠炎的临床疗效[J]. 中医临床研究,2010,2(11):38-39. DOI:10.3969/j.issn.1674-7860.2010.11.023.
19. 张怡,霍红. 美沙拉嗪肠溶片联合紫艾汤中药保留灌肠治疗溃疡性结肠炎的临床观察[J]. 中国老年学杂志,2010,30(9):1284-1285.
20. 张春燕. 中西医结合治疗溃疡性结肠炎的护理[J]. 中国美容医学,2010,19(z4):234.
21. 张淑英,魏月兰,潘永峰. 冠心宁注射液联合美沙拉嗪治疗溃疡性结肠炎疗效观察[J]. 临床合理用药杂志,2010,3(8):39.
22. 张瑜,张凤云,李海华. 中西医结合治疗溃疡性结肠炎43例[J]. 中国中医药现代远程教育,2010,8(11):53-54.
23. 张立英,庄克生,郭洪英,王彦敏,郭秀伏.美沙拉嗪联合中药保留灌肠治疗溃疡性结肠炎疗效分析60例[J].中国医药指南,2010,8(29):92-93.
24. 张红昌,韩淑凯,董进友.头针疗法联合柳氮磺胺吡啶治疗慢性溃疡性结肠炎46例疗效观察[J].中国中医急症,2010,19(02):220+243.
25. 彭红琼.半夏泻心汤加减治疗溃疡性结肠炎64例[J].检验医学与临床,2010,7(13):1377-1378.
26. 戴光耀,王海,王明祥,孙轶飞,王立伟,陈稳.柳氮磺胺吡啶结肠溶胶囊治疗溃疡性结肠炎疗效分析[J].河北医药,2011,33(02):208-209.
27. 李召兵. 中西医结合治疗溃疡性结肠炎临床疗效分析[J]. 中医临床研究,2010,2(16):7-7.
28. 李惠民. 中西医结合治疗溃疡性结肠炎36例[J]. 中国中医药现代远程教育,2010,8(9):31-32.
29. 李明,路文军,胡艺丰. 芪枳黄连汤治疗慢性持续性溃疡性结肠炎临床研究[J]. 中华中医药学刊,2010,28(7):1505-1506.
30. 李洪涛. 柳氮磺胺吡啶口服加中药灌肠治疗60例溃疡性结肠炎临床观察[J]. 中医学报,2010,25(3):495-496.
31. 李素萍,薛迪强,郑兴基.中西医结合治疗慢性溃疡性结肠炎疗效观察[J].现代中西医结合杂志,2010,19(12):1481-1482.
32. 李金莲,王天娇.中西医结合治疗溃疡性结肠炎效果观察[J].光明中医,2010,25(07):1257-1258.
33. 杨建新. 中西医结合治疗溃疡性结肠炎20例[J]. 内蒙古中医药,2010,29(8):25.
34. 林佐光. 复方灌肠液治疗溃疡性结肠炎的临床观察[J]. 中国临床实用医学,2010,04(5):3-5.
35. 柳传鸿. 中西医结合治疗慢性溃疡性结肠炎75例[J]. 陕西中医学院学报,2010,33(2):29-30.
36. 梁志勇,李玉华,张俊华,等. 复方青黛丸口服、中西药灌肠、TDP灯照射治疗慢性非特异性溃疡性结肠炎[J]. 健康必读（下旬刊）,2010(6):143-144.
37. 殷文娟. 中西药联用治疗慢性结肠炎50例[J]. 中国中医药现代远程教育,2010(18):54.
38. 王兆春,李耀龙. 中西医结合治疗溃疡性结肠炎131例疗效观察[J]. 热带医学杂志,2010,10(9):1108-1110.
39. 王少萍,康卫红. 清热解毒中药配合利多卡因保留灌肠治疗溃疡性结肠炎45例[J]. 陕西中医,2010,31(9):1143-1144.
40. 王悦辉,余智涛,肖依珠. 中药液改良结肠途径治疗溃疡性结肠炎临床观察[J]. 中国中医急症,2010,19(1):32,36.
41. 王洪斌. 柳氮磺胺吡啶口服联合中药保留灌肠治疗溃疡性结肠炎36例效果分析[J]. 中国医药指南,2010,8(25):95-96.
42. 王诗社. 芪术健脾汤灌肠保留治疗溃疡性结肠炎90例[J]. 中国中医药现代远程教育,2010,8(19):19.
43. 窦英磊.中西医结合治疗溃疡性结肠炎临床观察[J].内蒙古中医药,2010,29(16):23.
44. 罗明,万恒荣,陈海生,陈壮浩.白头翁汤治疗溃疡性结肠炎50例分析[J].中国中西医结合外科杂志,2010,16(04):463-465.
45. 肖敏,吕荣锋. 中西医结合治疗溃疡性结肠炎23例[J]. 中国中医药现代远程教育,2010,8(7):43.
46. 苏建春,于云华,塔衣尔江,黄明,马聪,张剑荣,党菊英,代尔曼.梅术溃结灌肠汤合美沙拉嗪治疗溃疡性结肠炎临床观察[J].新疆中医药,2010,28(04):31-32.
47. 苏淑娟. 中药灌肠联合美沙拉嗪治疗溃疡性结肠炎临床疗效及对IL-6、IL-8的影响[D].黑龙江中医药大学,2010.
48. 董保林.柳氮磺胺吡啶联合锡类散灌肠治疗溃疡性结肠炎临床观察[J].中国误诊学杂志,2010,10(07):1608.
49. 蒋艳茹,王玉浔,阎翠兰,蔡春江,安雅臣.改良愈疡汤灌肠配合护理干预对慢性非特异性溃疡性结肠炎的疗效观察[J].河北中医,2010,32(11):1720-1722.
50. 蔺志娟. 中西医结合治疗溃疡性结肠炎70例临床观察[J]. 中国保健营养（下半月）,2010, (11):110-111.
51. 薛育政,刘宗良,陆宇峰,戴泓,张萍芳.中药溃结Ⅰ号治疗溃疡性结肠炎临床研究[J].新中医,2010,42(07):21-22.
52. 许洁如. 中药溃克灵治疗溃疡性结肠炎的随机对照临床研究[D].南京中医药大学,2010.
53. 许雪全,王显飞. 美沙拉嗪联合锡类散灌肠治疗溃疡性结肠炎84例[J]. 中国中医药咨讯,2010,2(14):168-169.
54. 谢敏,杨肇寿. 中西医结合治疗慢性溃疡性结肠炎128例疗效观察[J]. 中国医药导报,2010(3):40,42.
55. 赵波. 中西医结合治疗慢性溃疡性结肠炎疗效观察[J]. 现代中西医结合杂志,2010,19(23):2908-2909.
56. 郁卫洲,王笑秋,胡顺明. 川芎嗪注射液辅助治疗溃疡性结肠炎60例[J]. 中国医药指南,2010,8(29):255-256.
57. 陈天亚. 中西医结合治疗溃疡性结肠炎的疗效分析[J]. 当代医学,2010,16(34):139,19.
58. 韦健盛,谭勇明,廖远庄. 中西医结合治疗溃疡性结肠炎33例[J]. 实用中医内科杂志,2010,24(9):10-12.
59. 韦明,唐奇端. 中西医结合灌肠治疗溃疡性结肠炎35例疗效观察[J]. 长春中医药大学学报,2010,26(6):891.
60. 韩立坤,狄亚杰,常宏. 丹参川芎嗪注射液治疗炎症性肠病的研究[J]. 现代中西医结合杂志,2010,19(30):3230-3231,3234.
61. 魏思忱,郑国启,孔郁,田树英,张秀刚,宋慧.谷参肠安对溃疡性结肠炎患者细胞因子的影响[J].中成药,2010,32(03):366-369.
62. 鲁龙生,何永恒,罗敏,罗育连,谭正洋,刘景.芍药汤治疗湿热内蕴型溃疡性结肠炎30例[J].中国中医药现代远程教育,2010,8(19):11-12.
63. 黄媛华,黄国栋.美珍颗粒剂灌肠联合美沙拉嗪口服治疗溃疡性结肠炎的疗效观察[J].中成药,2010,32(03):369-371.
64. 黄平富,刘守志.美沙拉嗪口服联合中药灌肠治疗溃疡性结肠炎临床观察[J].中国实用医药,2010,5(32):6-7.
65. 丁乾德.辨证治疗慢性溃疡性结肠炎疗效观察[J].四川中医,2011,29(06):64-65.
66. 于大海.美沙拉嗪配合中药灌肠治疗溃疡性结肠炎68例临床观察[J].中国社区医师(医学专业),2011,13(17):163-164.
67. 代英巍,荣淑贤,孙剑峰.锡类散等中西药联合灌肠治疗溃疡性结肠炎的疗效分析[J].中国误诊学杂志,2011,11(21):5108.
68. 代雯,范金萍.中西医结合治疗溃疡性结肠炎的临床护理体会[J].按摩与康复医学,2011(29):167-167.
69. 余天智,黎琮毅,黄宁生,梁英.补中益气汤加味联合康复新液保留灌肠治疗老年人溃疡性结肠炎的疗效观察[J].广西医学,2011,33(08):978-979.
70. 倪广婷,陈诗华.锡类散灌肠联合美沙拉嗪肠溶片口服治疗溃疡性结肠炎的疗效观察[J].江西医药,2011,46(10):923-924.
71. 农子彪.中药灌肠联合西药治疗溃疡性结肠炎疗效观察[J].广西中医药,2011,34(04):52-53.
72. 刘召茹,周茂京.美沙拉嗪联合川芎嗪治疗溃疡性结肠炎的临床疗效[J].中国当代医药,2011,18(29):86-87.
73. 刘彦平,周淑艳.黄芪注射液联合美沙拉嗪治疗溃疡性结肠炎42例[J].中国药业,2011,20(10):77-78.
74. 刘玉海. 中药保留灌肠合西医口服治疗溃疡性结肠炎40例疗效分析[J]. 中国中医药咨讯,2011,3(16):350.
75. 刘玉琼. 溃结合剂治疗溃疡性结肠炎的药物疗效观察[J]. 微量元素与健康研究,2011,28(3):33-35.
76. 刘秀丽.中西医结合治疗溃疡性肠炎疗效分析[J].中国医药指南,2011,9(33):171-172.
77. 吕利亚. 中西医结合治疗溃疡性结肠炎26例[J]. 内蒙古中医药,2011,30(20):21-21.
78. 吕小青. 中西医结合治疗溃疡性结肠炎的疗效及临床分析[J]. 健康必读（下旬刊）,2011(11):74-74.
79. 吴颖,罗武陵. 复方黄柏液结肠水疗辅助治疗溃疡性结肠炎40例[J]. 中国中医急症,2011,20(7):1174-1175.
80. 周欣,邓静雯,黄国栋,等. 美珍颗粒剂灌肠配合音乐疗法治疗溃疡性结肠炎的疗效观察[J]. 中国肛肠病杂志,2011,31(4):19-21.
81. 周正颜,李海强. 艾箱灸治疗慢性溃疡性结肠炎的疗效观察及护理[J]. 中国社区医师（医学专业）,2011,13(19):299-300.
82. 孔卫华. 中药灌肠配合美沙拉嗪治疗溃疡性结肠炎疗效观察[C]. //中国肛肠病研究心得集. 2011.
83. 孙金辉,何成伟. 柳氮磺吡啶联合四神丸超微颗粒剂保留灌肠治疗中度溃疡性结肠炎脾肾阳虚证疗效观察[J]. 天津中医药,2011,28(1):27-29.
84. 席作武,王凯,刘文清. 惠迪口服联合葛根芩连汤保留灌肠治疗湿热型溃疡性结肠炎67例临床研究[J]. 中国肛肠病杂志,2011,31(10):38-40.
85. 廉敏. 中西医结合治疗溃疡性结肠炎50例[J]. 中国医药导报,2011,8(30):118-120.
86. 张元澧,梁玉杰,段永强,刘建发,李重昆,朱立鸣.痛泻二草方联合柳氮磺吡啶治疗溃疡性结肠炎32例[J].中医研究,2011,24(12):19-21.
87. 张国荣. 中西医结合治疗溃疡性结肠炎的临床研究[J]. 中国现代医生,2011,49(19):159-160.
88. 张大成. 中西医结合治疗溃疡性结肠炎118例临床观察[J]. 长春中医药大学学报,2011,27(1):97.
89. 张广武.中药内服加灌肠治疗溃疡性结肠炎[J].湖北中医杂志,2011(12):55-56.
90. 张莉,尹丽菊. 中西结合治疗慢性非特异性溃疡性结肠炎82例临床观察[J]. 河北中医,2011,33(7):1029-1029.
91. 徐权胜,肖新李,黄春旭.中药保留灌肠治疗溃疡性结肠炎疗效观察[J].实用中西医结合临床,2011,11(02):30-31.
92. 徐纪文,刘长运.连理汤治疗溃疡性结肠炎作用与机制研究[J].内蒙古中医药,2011,30(24):21-22.
93. 戴巧君,李敏雅. 中西医结合治疗溃疡性结肠炎43例[J]. 浙江中医杂志,2011,46(11):825-826.
94. 戴高中,范先靖,赵克学,诸静芬,沈旦蕾.“溃结安”联合柳氮磺胺吡啶灌肠治疗非特异性溃疡性结肠炎急性期的临床研究[J].江苏中医药,2011,43(01):20-21.
95. 房栩丞. 中药保留灌肠配合西药口服治疗溃疡性结肠炎临床观察[C]//.中国肛肠病研究心得集.,2011:416-417.
96. 施丽婕. 化瘀通阳方对溃疡性结肠炎肠屏障血液高凝状态干预的临床观察[C]//.中华中医药学会脾胃病分会第二十三次全国脾胃病学术交流会论文汇编.[出版者不详],2011:459-460.
97. 朱代林,冯钢,曾德志,王兵华.低频振动电磁治疗仪治疗溃疡性结肠炎临床观察[J].海南医学院学报,2011,17(04):491-494.
98. 朱金庆.地塞米松配合云南白药灌肠治疗溃疡性结肠炎效果分析[J].中医临床研究,2011,3(08):45-46.
99. 李军,姜红英,王健.复方黄柏液保留灌肠治疗溃疡性结肠炎疗效观察[J].辽宁中医杂志,2011,38(01):108-110.
100. 李国进,王学员.白头翁汤结合西药治疗溃疡性结肠炎48例[J].上海中医药杂志,2011,45(07):45-46.
101. 李明,曾艳.黄芪建中汤为主治疗慢性溃疡性结肠炎60例[J].陕西中医,2011,32(09):1134-1135.
102. 李春耕,李洪涛,李淑娟. 中西医结合灌肠治疗直乙型溃疡性结肠炎临床观察[J]. 中国中医药咨讯,2011,3(13):94.
103. 李曙光,孙华波.中药灌肠治疗中重度溃疡性结肠炎的疗效观察[J].实用心脑肺血管病杂志,2011,19(07):1228.
104. 李翠.美沙拉嗪联合中药灌肠治疗溃疡性结肠炎临床研究[J].中医临床研究,2011,3(03):15-16.
105. 李范君.联合用药治疗溃疡性结肠炎患者疗效探讨[J].中国医学工程,2011,19(01):72.
106. 李雪芹.肠舒汤联合柳氮磺吡啶治疗溃疡性结肠炎临床观察[J].中国误诊学杂志,2011,11(15):3562-3563.
107. 杜坤庭,葛勤利,杨伟捷,等. 柳氮磺吡啶结肠溶胶囊联合云南白药治疗溃疡性结肠炎[J]. 中国基层医药,2011,18(18):2465-2466.
108. 杜文武,张亚峰. 中西医结合治疗溃疡性结肠直肠炎疗效观察[J]. 西部中医药,2011,24(9):80-81.
109. 杨周瑞. 扶阳清肠汤治疗溃疡性结肠炎的临床观察[C]. //2011首届国际扶阳论坛暨第四届全国扶阳论坛论文集. 2011.
110. 杨旭. 中药气药灌肠法对溃疡性结肠炎患者食物不耐受状态影响的研究[D]. 江苏:南京中医药大学,2011.
111. 杨琦,赵靖松,杨凯. 云南白药联合锡类散保留灌肠治疗溃疡性结肠炎的疗效观察[J]. 中国实用医药,2011,6(25):156-157.
112. 杨红娟,赵凊玲,赵曙光. 艾灸神阙穴治疗慢性结肠炎50例效果观察[J]. 齐鲁护理杂志,2011,17(34):封3.
113. 林森. 中药灌肠治疗溃疡性结肠炎71例临床分析[J]. 健康必读（中旬刊）,2011(5):82.
114. 毛晓斌,刘永芬,陈爱民. 美沙拉嗪联合中药穴位帖敷治疗溃疡性结肠炎效果观察[J]. 实用中西医结合临床,2011,11(2):28-29.
115. 毛海滨. 中西医结合治疗慢性结肠炎临床观察[J]. 中国中医药咨讯,2011,3(6):181.
116. 汤建生. 复方苦参结肠溶胶囊联合SASP治疗溃疡性结肠炎20例[J]. 承德医学院学报,2011,28(4):368-370.
117. 汪萍波,赵杏芳. 葛根芩连汤联合柳氮磺胺吡啶保留灌肠治疗溃疡性结肠炎的疗效及安全性观察[J]. 中国医药指南,2011,9(1):131-132.
118. 沈光茂. 中西医结合治疗溃疡性结肠炎40例临床观察[J]. 黑龙江中医药,2011,40(6):12-13.
119. 沈建法,宋树斌,高利强. 芍药汤加味联合美沙拉嗪治疗溃疡性结肠炎30例[J]. 中国中医急症,2011,20(6):991-992.
120. 牟景敏,龙梅,王靖. 中药溃结煎配合西药治疗慢性溃疡性结肠炎40例疗效观察[J]. 中国肛肠病杂志,2011,31(3):55-56.
121. 王国香.锡类散中药煎剂灌肠治疗溃疡性结肠炎的临床观察[J].中国民康医学,2011,23(04):445+469.
122. 王家华.葛根芩连汤治疗溃疡性结肠炎的疗效观察[J].现代医药卫生,2011,27(22):3462.
123. 王永青,孙承洪.中西医结合治疗溃疡性结肠炎体会[J].中国医学工程,2011,19(11):115.
124. 王志刚. 中西医结合治疗溃疡性结肠炎65例临床疗效[J]. 中国中医药咨讯,2011,3(14):449.
125. 王玲君,王志民,孙嘉伟,等. 柳氮磺吡啶口服联合锡类散保留灌肠治疗溃疡性结肠炎的疗效探讨[J]. 中国肛肠病杂志,2011,31(12):50-51.
126. 王金艳. 中西药保留灌肠治疗溃疡性结肠炎的疗效观察[J]. 中国中医药咨讯,2011,3(14):436.
127. 田蕾,李舒. 中药保留灌肠治疗溃疡性结肠炎的临床观察[J]. 中国药房,2011,22(23):2189-2191.
128. 白雪松,刘伟,张春阳.丹参联合美沙拉嗪对溃疡性结肠炎患者乳铁蛋白的影响[J].吉林医学,2011,32(18):3645.
129. 瞿惠燕.白及苦参汤保留灌肠联合柳氮磺胺吡啶治疗溃疡性结肠炎临床观察[J].上海中医药杂志,2011,45(12):56-57.
130. 石晓玲.中西药联用灌肠治疗慢性溃疡性结肠炎观察及护理[J].实用中医药杂志,2011,27(10):698-699.
131. 石生源. 慢性溃疡性结肠炎临床治疗分析[J]. 中外医疗,2011,30(34):83.
132. 祝宇平,王玲玉. 中药灌肠治疗溃疡性结肠炎47例的疗效观察及护理[J]. 中医外治杂志,2011,20(6):9-10.
133. 葛娅琳,吴晖. 美沙拉嗪口服联合锡类散灌肠治疗溃疡性结肠炎的临床疗效观察[J]. 海峡药学,2011,23(9):114-115.
134. 董莹,解刘松. 溃速康汤联合美沙拉嗪治疗直肠及乙状结肠溃疡性结肠炎56例[J]. 中医药导报,2011,17(7):32-33.
135. 蒋崇福,张述平,刘燕. 联用补脾益肠丸和柳氮磺吡啶治疗溃疡性结肠炎的临床观察[J]. 临床医学工程,2011,18(3):359-360.
136. 蒋艳茹,姚娜,王玉浔,等. 改良愈疡汤灌肠治疗溃疡性结肠炎疗效观察[J]. 陕西中医,2011,32(7):861-863.
137. 衣丽虹. 中西医结合治疗消化性溃疡50例疗效观察[J]. 中外健康文摘,2011,8(18):210-211.
138. 谭震宇. 中西医结合治疗溃疡性结肠炎52例临床观察[J]. 浙江中医杂志,2011,46(12):901.
139. 许可银. 中西医结合治疗溃疡性结肠炎的临床疗效观察[J]. 现代医药卫生,2011,27(13):2031-2032.
140. 赵浩然. 美沙拉嗪联合资生汤治疗溃疡性结肠炎52例疗效观察[J]. 中外医学研究,2011,9(23):53-54.
141. 赵磊. 中西医结合治疗溃疡性结肠炎优化方案的研究[D]. 山东:山东中医药大学,2011.
142. 赵立军,赵立民. 中西医结合治疗溃疡性结肠炎80例疗效观察[J]. 现代中西医结合杂志,2011,20(36):4673-4674.
143. 迟莉丽,于明明. 健脾愈疡方神阙穴贴敷配合柳氮磺胺吡啶治疗慢性溃疡性结肠炎44例[J]. 实用中医内科杂志,2011,25(8):55-57.
144. 邓健敏,韩宇斌,陈建林,等. 中西医治疗溃疡性结肠炎对细胞因子及疗效影响[J]. 中国实用医药,2011,6(20):107-108.
145. 郑树青. 中西药合用保留灌肠治疗溃疡性结肠炎临床分析[J]. 实用中医药杂志,2011,27(12):856-857.
146. 陈四辈,盛长健,邹晓华. 加味溃愈汤联合柳氮磺胺吡啶治疗溃疡性结肠炎临床观察[J]. 中医药临床杂志,2011,23(3):239-241.
147. 陈德凤,高天,范晓娇. 溃疡性结肠炎病人应用盒灸的效果评价[J]. 护理研究,2011,25(34):3151-3152.
148. 陈观尚. 溃疡性结肠炎患者C反应蛋白的变化及美沙拉嗪联合白头翁汤灌肠的疗效观察[J]. 广东医学院学报,2011,29(2):147-148.
149. 雒福东. 血竭治疗活动期溃疡性结肠炎的实验及临床研究[D]. 四川:成都中医药大学,2011.
150. 高富明,高阳,彭玉亮,等. 复方诺氟沙星灌肠液加血竭粉锡类散保留灌肠治疗溃疡性结肠炎33例[J]. 中国中西医结合消化杂志,2011,19(6):406-407.
151. 高峰,葛亚强,张中平. 中西医结合治疗溃疡性结肠炎30例临床观察[J]. 江苏中医药,2011,43(12):40-41.
152. 鲍良生,孔梅. 中西医结合治疗活动期溃疡性结肠炎的疗效[J]. 皖南医学院学报,2011,30(6):480-482.
153. 黄光斌. 160例溃疡性结肠炎中西医结合治疗效果分析[J]. 医药前沿,2011,1(10):72-73.
154. Gong Y, Zha Q, Li L, Liu Y, Yang B, Liu L, Lu A, Lin Y, Jiang M. Efficacy and safety of Fufangkushen colon-coated capsule in the treatment of ulcerative colitis compared with mesalazine: a double-blinded and randomized study. J Ethnopharmacol. 2012 Jun 1;141(2):592-8. doi: 10.1016/j.jep.2011.08.057. Epub 2011 Sep 6. PMID: 21911045.
155. 丁建华. 中药保留灌肠联合西药治疗溃疡性结肠炎临床观察[J]. 长春中医药大学学报,2012,28(5):867-868.
156. 任秀梅,刘少宁,董志强. 云南白药与锡类散保留灌肠联合口服美沙拉嗪治疗溃疡性结肠炎的疗效观察[J]. 现代消化及介入诊疗,2012,17(4):231-232.
157. 刘别影,王少渊,侯英奎,张剑锋,张凯,马刚,王白波.康复新液合锡类散保留灌肠治疗溃疡性结肠炎疗效观察[J].中国保健营养,2012,22(08):1008-1009.
158. 刘祥,黄修海,毕超.结肠透析仪结肠灌洗联合中药保留灌肠治疗炎症性肠病疗效观察[J].中国中西医结合消化杂志,2012,20(07):311-313.
159. 单伟峰,李艳丽,杨元生.溃疡性结肠炎患者甲硝唑联合中药灌肠的疗效观察[J].齐齐哈尔医学院学报,2012,33(12):1627-1628.
160. 吴中平,吴晓茹,徐意,汤世伟.中药保留灌肠联合美沙拉秦对溃疡性结肠炎患者血清肿瘤坏死因子-α、白细胞介素-6的影响[J].中国中西医结合消化杂志,2012,20(11):510-511.
161. 周毅,刘红华,叶松. 中药灌肠联合西药治疗溃疡性结肠炎的疗效观察[J]. 湖北中医杂志,2012,34(4):9-10.
162. 周燕. 乌梅丸治疗溃疡性结肠炎的疗效观察[D].南京中医药大学,2012.
163. 宋玉梅,秦佰焰. 中西医结合治疗慢性溃疡性结肠炎49例[J]. 中国实验方剂学杂志,2012,18(16):324-326.
164. 容海鹰,杨元生,彭卫斌,朱雅丽.美沙拉嗪联合云南白药灌肠对溃疡性结肠炎患者的临床研究[J].中国医药指南,2012,10(19):40-41.
165. 常有. 粪钙卫蛋白与溃疡性结肠炎的相关性研究及中药气药灌肠治疗的疗效分析[D].南京中医药大学,2012.
166. 廖信茜.中西医结合治疗近段溃疡性结肠炎的临床疗效观察[J].现代诊断与治疗,2012,23(10):1655-1656.
167. 张冰. 肠愈宁颗粒对活动期溃疡性结肠炎（大肠湿热型）患者肠粘膜中Toll样受体4表达影响的研究[D].黑龙江中医药大学,2012.
168. 张勇. 中西医结合治疗溃疡性结肠炎80例观察[J]. 实用中医药杂志,2012,28(3):193-193.
169. 张合红.中西医结合治疗溃疡性结肠炎44例[J].中医临床研究,2012,4(21):61+63.
170. 张娟,曹泽伟.清肠愈疡汤联合美沙拉嗪治疗溃疡性结肠炎的疗效分析[J].中国全科医学,2012,15(01):90-92.
171. 张宁,黄颖娴,刘革兰,杨茜湄.中药加美沙拉嗪肠溶片灌肠治疗慢性溃疡性结肠炎的疗效观察与护理[J].中国医药指南,2012,10(18):347-348.
172. 张新华,耿梅. 中西医结合治疗溃疡性结肠炎30例[J]. 中国中医药现代远程教育,2012,10(13):69-70.
173. 张曦. 中西医结合治疗慢性溃疡性结肠炎疗效观察[J]. 中国卫生产业,2012(5):155.
174. 张月凡,李楠,张林,翟俊山,吴凯,王艳梅.远端型溃疡性结肠炎临床特点及复方血竭灌肠疗效分析[J].军医进修学院学报,2012,33(05):470-472+515.
175. 张璐璐,宋雪诗. 美沙拉嗪口服联合中药灌肠治疗溃疡性结肠炎疗效观察[J]. 中国误诊学杂志,2012,12(14):3620-3621.
176. 张迎泉,王红艳,朱应福. 清解运脾和血法调控溃疡性结肠炎患者血清TNF-α、IL-6的临床研究[J]. 中医临床研究,2012,4(4):71-73.
177. 徐国荣. 中西药联用保留灌肠治疗慢性溃疡性结肠炎50例疗效观察[J]. 浙江中医杂志,2012,47(6):426.
178. 徐玉玲. 中西医结合治疗肝郁脾虚型溃疡性结肠炎的临床疗效[J]. 中国医药科学,2012,2(10):101-102.
179. 施丽婕,杨强,垢敬,陈大权,王静,周正华.化瘀通阳灌肠方对溃疡性结肠炎患者高凝状态的影响[J].中华中医药杂志,2012,27(08):2155-2157.
180. 施文杰.红藤灌肠液灌肠联合SASP治疗溃疡性结肠炎40例[J].黑龙江中医药,2012,41(04):23.
181. 曲连军. 中西医结合治疗溃疡性结肠炎的疗效分析[J]. 中国医药科学,2012,2(22):86-87.
182. 李军,李红,赵玉娟,赵克.中西医结合治疗溃疡性结肠炎的临床观察[J].北京中医药,2012,31(01):56-58.
183. 李卫玲,李红刚.痛泻要方及四神丸配合西药治疗溃疡性结肠炎52例[J].陕西中医,2012,33(05):528-529.
184. 李娜.中西医结合治疗溃疡性结肠炎36例观察[J].实用中医药杂志,2012,28(10):848-849.
185. 李康,刘旭明,王强.白头翁汤、理中汤灌肠治疗溃疡性结肠炎疗效观察[J].辽宁医学院学报,2012,33(05):430-431.
186. 李成田.中西医结合治疗溃疡性结肠炎疗效观察[J].现代中西医结合杂志,2012,21(14):1533+1536.
187. 李日光.补中益气丸治疗溃疡性结肠炎80例临床观察[J].中国实用医药,2012,7(07):172-173.
188. 李明. 肠愈宁联合美沙拉嗪治疗溃疡性结肠炎的临床观察[J]. 中国农村卫生,2012(z2):90.
189. 李朱明. 芍药汤加味联合奥沙拉秦钠治疗湿热内蕴型活动期溃疡性结肠炎对照研究[J]. 实用中医内科杂志,2012,26(5):74-75.
190. 李森娟,季霞,邱敏,凌怡庭.乌梅汤治疗溃疡性结肠炎70例分析[J].浙江中医药大学学报,2012,36(06):656-657.
191. 李灿群,解刘松,岳栋.中药灌肠联合柳氮磺吡啶肠溶片治疗溃疡性结肠炎的效果观察[J].当代护士(中旬刊),2012(03):111-112.
192. 李辉.中西医结合治疗慢性溃疡性结肠炎68例临床观察[J].江苏中医药,2012,44(06):42-43.
193. 李静. 芍芪椿皮汤治疗大肠湿热型溃疡性结肠炎的临床疗效观察[D].山东中医药大学,2012.
194. 杜山鹏,侯媛,陈宝和.美沙拉嗪配伍中药保留灌肠治疗溃疡性结肠炎的临床观察[J].中医临床研究,2012,4(11):53-54.
195. 杜强,毛刚,何玲,龚枚.溃愈汤保留灌肠治疗活动期溃疡性结肠炎疗效观察[J].四川中医,2012,30(04):68-69.
196. 杜维成,王志刚,汪世平.芪参肠泰肠溶胶囊治疗溃疡性结肠炎30例[J].中医研究,2012,25(07):27-29.
197. 杨彬.美沙拉嗪肠溶片口服联合中药灌肠治疗溃疡性结肠炎的疗效观察[J].中国现代药物应用,2012,6(24):78-80.
198. 杨沈秋,郑丽红,张禹,王楠楠,刘定,姚丹.美沙拉嗪联合“于氏头针”法治疗溃疡性结肠炎临床疗效观察[J].中医药信息,2012,29(03):96-98.
199. 林小芬,余杨,张伟艺,余凯云,汪润,饶泽珍.中西医结合治疗溃疡性结肠炎33例临床体会[J].中国医药指南,2012,10(27):275.
200. 毕伟平,苘辉斌,荣晓峰.美沙拉嗪与中药保留灌肠联用治疗溃疡性结肠炎临床研究[J].中国社区医师(医学专业),2012,14(05):197-198.
201. 沈玉. 中西医综合治疗溃疡性结肠炎43例[J]. 中医研究,2012,25(7):41-42.
202. 潘庚,朱桢.改良康复新液保留灌肠治疗溃疡性结肠炎疗效及对血清凝血指标的影响[J].河北医药,2012,34(11):1623-1625.
203. 王中甫,韩瑞锋. 中西医结合治疗溃疡性结肠炎的临床观察[J]. 中国医疗前沿,2012,7(14):21-22.
204. 王怀珍,李晓云,姚玉花. 中西医结合治疗溃疡性结肠炎的临床体会[J]. 中国民族民间医药,2012,21(11):103.
205. 王振江. 四逆黄芪汤治疗肝郁脾虚型溃疡性结肠炎的临床研究[D]. 山东:山东中医药大学,2012.
206. 王晓霞. 美沙拉嗪联合中药灌肠治疗溃疡性结肠炎的临床观察[J]. 中外医疗,2012,31(32):77-78.
207. 王若腾,徐伟刚. 中西医结合治疗慢性非特异性溃疡性结肠炎[J]. 中国现代医生,2012,50(9):76-77,83.
208. 王蕾. 中西药结合治疗溃疡性结肠炎42例疗效观察[J]. 赤峰学院学报（自然科学版）,2012(24):102-103.
209. 王靖.应用中西医结合多途径治疗近段溃疡性结肠炎的疗效[J].中国卫生产业,2012,9(33):172.
210. 盛英丽.鸦胆子油口服乳液保留灌肠治疗溃疡性结肠炎75例效果观察[J].齐鲁护理杂志,2012,18(16):12-14.
211. 肖克安.云南白药胶囊治疗溃疡性结肠炎腹痛的疗效观察[J].中国当代医药,2012,19(35):77+79.
212. 肖高健,游旭东,王占波.中西医结合治疗溃疡性结肠炎左半结肠型42例[J].中国药物经济学,2012(03):207-208.
213. 苑坤,齐玲芝,于燕.舒血宁注射液治疗溃疡性结肠炎的疗效观察[J].中国实用医药,2012,7(32):145-146.
214. 范崇信.中西医结合治疗溃疡性结肠炎的临床观察[J].中国医药指南,2012,10(33):291-292.
215. 葛飞,马小平,肖明兵.电针联合柳氮磺吡啶治疗溃疡性结肠炎效果观察[J].交通医学,2012,26(02):173-174.
216. 蔡学兵.三黄汤加味保留灌肠联合美沙拉嗪治疗溃疡性结肠炎疗效观察[J].中国实用医药,2012,7(18):166-167.
217. 蔡明建,朱迪. 中西药结合治疗溃疡性结肠炎临床疗效观察[J]. 医学信息,2012,25(1):274-275.
218. 谢晶日,刘朝霞,许明月. 观察肠愈宁治疗溃疡性结肠炎前后肠炎症活动指数评分[C]. //第二十五届全国中西医结合消化系统疾病学术会议论文集. 2013:236-236.
219. 谢晶日,陆振华,刘朝霞,等. 肠愈宁对活动期溃疡性结肠炎患者肠黏膜NF-κB mRNA表达的影响[J]. 中医药信息,2012,29(3):40-42.
220. 谭艳芳. 中西医结合治疗对溃疡性结肠炎疗效的影响研究[J]. 心理医生（下半月版）,2012(8):136-137.
221. 赵敏. 拟益气清热汤治疗溃疡性结肠炎的临床疗效[J]. 健康必读（中旬刊）,2012,11(11):68-68.
222. 赵毅杰. 中西医结合治疗溃疡性结肠炎40例临床观察[J]. 基层医学论坛,2012,16(20):2665-2665.
223. 赵甲英. 美沙拉嗪口服联合锡类散灌肠治疗老年溃疡性结肠炎的疗效[J]. 吉林医学,2012,33(16):3459-3460.
224. 赵莹. 60例溃疡性结肠炎中西医结合治疗临床观察[J]. 内蒙古中医药,2012,31(2):34-35.
225. 辛涛. 美沙拉嗪联合中药脐疗治疗溃疡性结肠炎临床研究[J]. 中国中医药咨讯,2012,4(1):31-32.
226. 邓世明. 中西医结合治疗溃疡性结肠炎临床分析[J]. 中国中医药咨讯,2012,4(5):302.
227. 邓国法. 中西药结合与单用西药治疗溃疡性结肠炎的疗效对比分析[J]. 医学信息,2012,25(12):154.
228. 郝帅. 中药溃结康保留灌肠治疗溃疡性结肠炎的临床疗效评价[D].辽宁中医药大学,2012.
229. 钟山.慢性溃疡性结肠炎50例的中西医结合治疗体会[J].内蒙古中医药,2012,31(09):59.
230. 钟锐生,陈桂红. 参苓白术散治疗溃疡性结肠炎的疗效及对患者免疫状态的影响[J]. 中国基层医药,2012,19(22):3391-3393.
231. 闫守月,邱胜民,吕辉. 综合疗法治疗溃疡性结肠炎76例疗效观察[C]//.2012医学前沿——中华中医药学会肛肠分会第十四次全国肛肠学术交流大会论文精选.,2012:264-266.
232. 陆振华. 肠愈宁颗粒对活动期溃疡性结肠炎（大肠湿热型）患者肠黏膜中的核因子-KB影响的研究[D].黑龙江中医药大学,2012.
233. 席作武,刘文清,王凯.惠迪口服联合加味附子理中汤保留灌肠治疗脾肾阳虚型溃疡性结肠炎临床研究[J].中医学报,2011,26(09):1114-1116.
234. 陈伟,张亚锋,权隆芳. 自拟中药保留灌肠治疗溃疡性结肠炎的临床分析[J]. 医药前沿,2012(33):346-347.
235. 陈娇娥. 柳氮磺吡啶、氢化可的松、刺五加注射液联合灌肠治疗溃疡性结肠炎的临床研究[J]. 河北医科大学学报,2012,33(6):629-631.
236. 陈建辉. 自拟溃迅康方联合畅美(奥沙拉嗪钠)治疗溃疡性结肠炎(活动期)临床观察[D]. 湖北:湖北中医药大学,2012.
237. 陈正超. 中西药结合治疗溃疡性结肠炎的临床疗效和安全性研究[J]. 中国医药指南,2012,10(20):433-434.
238. 陶伟. 芍药汤加减联合灌肠治疗活动期湿热型溃疡性结肠炎的临床观察[D]. 江苏:南京中医药大学,2012.
239. 韩灵善. 中西医结合治疗溃疡性结肠炎36例临床观察[J]. 卫生职业教育,2012,30(17):153-154.
240. 高富明. 血竭治疗活动期溃疡性结肠炎临床及实验研究[D].成都中医药大学,2012.
241. 高永珍,秦新荣,李雪梅,郝文静.甘草调中汤治疗溃疡性结肠炎的临床疗效[J].光明中医,2012,27(05):925-926.
242. 黄柳向,刘振杰.仙榆汤治疗慢性持续性溃疡性结肠炎的临床研究[J].中医临床研究,2012,4(17):7-8+11.
243. 黄革红,沈桂荣.中西医结合保留灌肠治疗溃疡性结肠炎临床观察[J].内蒙古中医药,2012,31(23):63-64.
244. 丁道峰. 芍药甘草汤加味口服加保留灌肠治疗溃疡性结肠炎临床研究[D]. 江苏:南京中医药大学,2013.
245. 尹清辉.中西医结合治疗溃疡性结肠炎临床观察[J].中国保健营养,2013,23(04):972-973.
246. 仇瑞莉.中西医结合治疗中度溃疡性结肠炎(湿热内蕴证)45例疗效观察[J].国医论坛,2013,28(06):38-39.
247. 任范文,张云志.复方黄柏液联合柳氮磺吡啶加氢化可的松治疗溃疡性结肠炎的临床观察[J].吉林医学,2013,34(20):4042-4043.
248. 何泽生,钟世彪.雷火灸联合柳氮磺吡啶治疗轻、中度溃疡性结肠炎30例[J].长春中医药大学学报,2013,29(06):1051-1053.
249. 余晓,杨俊.呋喃唑酮片、锡类散联合蒙脱石散剂保留灌肠对溃疡性结肠炎的疗效观察[J].中国医刊,2013,48(05):46-48.
250. 党滢,王鹏希. 中西药结合治疗溃疡性结肠炎64例临床研究[J]. 健康大视野,2013,21(12):112-112.
251. 冯玲吉,丁明红,王成雪. 肠澼Ⅰ号方治疗溃疡性结肠炎疗效观察[J]. 山东医药,2013,53(26):84-85.
252. 凡荣喜,龚丽萍,赵丽娟. 中药保留灌肠治疗溃疡性结肠炎的护理观察[J]. 黑龙江医药,2013,26(3):537-538.
253. 刘剑君,李华翔,李廷超.美沙拉嗪结合中药灌肠在治疗溃疡性结肠炎中的临床应用[J].吉林医学,2013,34(06):1088-1089.
254. 刘启旺,柯素霞,牛立军.中药灌肠联合美沙拉嗪栓治疗直肠型溃疡性结肠炎的临床观察[J].四川中医,2013,31(08):80-82.
255. 刘宏晶,刘茂坤,苗德芳,冯文涛.中药灌肠联合美沙拉嗪及布拉氏酵母菌治疗溃疡性结肠炎34例[J].环球中医药,2013,6(12):943-946.
256. 刘超.丹参注射液联合双歧三联活菌佐治溃疡性结肠炎36例[J].中国药业,2013,22(18):97.
257. 刘青春.中药疏肝健脾止痢方联合5-氨基水杨酸治疗慢性溃疡性结肠炎的临床疗效及其对血清TNF-α水平的影响[J].中国中西医结合消化杂志,2013,21(01):29-31.
258. 刘颖.中西医结合治疗溃疡性结肠炎临床研究[J].中医学报,2013,28(03):418-419.
259. 匡思祯.自制灌肠合剂治疗炎症性肠病的临床观察[J].江西医药,2013,48(09):800-802.
260. 卢霞. 辩证治疗溃疡性结肠炎54例[J]. 中国保健营养（下旬刊）,2013,23(12):7695.
261. 史一成,黄湘霞. 中药口服加灌肠为主治疗活动期溃疡性结肠炎26例[J]. 浙江中医杂志,2013,48(9):656.
262. 叶彬,樊德利. 蒲芩败酱汤与美沙拉嗪灌肠对溃疡性结肠炎患者血清TNF-α及IL-10的影响[J]. 浙江中医杂志,2013,48(7):476-477.
263. 肖克安. 云南白药胶囊治疗溃疡性结肠炎腹痛的疗效观察[J]. 中国当代医药,2012,19(35):77,79.
264. 吴晓军.中西医结合治疗慢性溃疡性结肠炎的疗效[J].求医问药(下半月),2013,11(02):487-488.
265. 吴爱玲. 中西医联合多途径治疗近段溃疡性结肠炎临床疗效评价[J]. 健康大视野,2013,21(18):281-282.
266. 吾米提汗·热合曼. 中西医结合治疗溃疡性结肠炎脾胃虚弱型的临床对比观察[J]. 贵阳中医学院学报,2013,35(4):150-151.
267. 周毅,叶松. 中药灌肠联合西药治疗溃疡性结肠炎的疗效观察[C]. //中华中医药学会脾胃病分会第二十五届全国脾胃病学术交流会论文集. 2013:466-466.
268. 周燕,费建平,李保良. 乌梅丸汤剂联合柳氮磺胺吡啶对溃疡性结肠炎患者细胞免疫功能的影响[J]. 中成药,2013,35(12):2760-2761.
269. 姜成军. 中西药保留灌肠治疗慢性复发型溃疡性结肠炎急性发作期的临床观察[J]. 中国中医药科技,2013,20(3):294-295.
270. 孙冰. 中西医结合治疗溃疡性结肠炎160例疗效观察[J]. 河北医学,2013,19(10):1553-1555.
271. 宋尚熙. 慢性溃疡性结肠炎芍药汤与痛泻要方联用的临床疗效观察[J]. 医学信息（下旬刊）,2013,26(10):207.
272. 宋雨鸿,贺亮,拾慧,方建志,潘锦瑶,蔡敬宙,陈励,徐舒.黄芩汤颗粒剂治疗大肠湿热型溃疡性结肠炎疗效观察[J].吉林中医药,2013,33(02):159-160.
273. 宋顺福. 参苓白术散治疗气虚型溃疡性结肠炎59例[J]. 江西中医药,2013(6):50-51.
274. 崔刚.中西医结合治疗49例溃疡性结肠炎的疗效观察[J].中国医药指南,2013,11(03):256-257.
275. 干晓花. 穴位敷贴法治疗脾虚型溃疡性结肠炎的临床观察[D].南京中医药大学,2013.
276. 张千娥.柳氮磺胺吡啶口服联合中药灌肠治疗溃疡性结肠炎的临床疗效观察[J].中国医院药学杂志,2013,33(22):1902-1904.
277. 张国彦. 锡类散联合美沙拉嗪肠溶片治疗溃疡性结肠炎35例[J]. 中国药业,2013,22(18):99.
278. 张居劲. 中西医结合治疗慢性溃疡性结肠炎疗效观察[J]. 健康大视野,2013,21(8):151.
279. 张晓凤. 中西医结合治疗溃疡性结肠炎的效果分析[J]. 吉林医学,2013,34(15):2964.
280. 张磊昌,张森,钟武,龙军先,李修宁,陈利生.壮医药线点灸联合柳氮磺吡啶治疗轻、中度溃疡性结肠炎的随机对照研究[J].针刺研究,2013,38(05):399-402.
281. 张福兴,江一平.中西医结合治疗溃疡性结肠炎30例[J].江西中医药,2013,44(09):31-32.
282. 张美艳. 中西医结合治疗溃疡性结肠炎的临床观察[J]. 健康大视野,2013,21(18):385-385.
283. 张荣. 肠炎康联合美沙拉嗪治疗溃疡性结肠炎的护理体会[J]. 中外女性健康（下半月）,2013(7):42-42.
284. 张颖,王玉玲.采用中西医结合方法治疗溃疡性结肠炎疗效观察[J].中国中西医结合外科杂志,2013,19(01):53-55.
285. 徐进康,陆喜荣,徐宏伟,吴坚芳,许邹华,陶鸣浩.溃结灵对溃疡性结肠炎患者DNA酶Ⅰ、Fascin蛋白表达的影响[J].中医杂志,2013,54(01):45-47.
286. 方晓锦,苏燕妮,黄雅慧.中药保留灌肠治疗溃疡性结肠炎36例疗效观察[J].湖南中医杂志,2013,29(11):42-43.
287. 施伟. 美沙拉嗪结合中药灌肠在治疗溃疡性结肠炎中的临床应用[J]. 医学信息,2013(13):297-297,298.
288. 曾祥武. 参苓白术散联合维柳芬治疗溃疡性结肠炎的临床效果观察[J]. 中国医疗前沿,2013(22):26-26,27.
289. 朱刚. 中西医结合治疗溃疡性结肠炎58例临床分析[J]. 北方药学,2013(12):24-24,25.
290. 朱厚荣. 美沙拉嗪肠溶片口服联合锡类散保留灌肠治疗溃疡性结肠炎的临床疗效性[J]. 中国医药指南,2013(25):58-58,59.
291. 朱清,黄重发,施斌斌. 口服与灌肠联合口服给药治疗溃疡性结肠炎临床对比研究[J]. 临床和实验医学杂志,2013,12(24):1982-1984.
292. 李俊. 中西医结合治疗溃疡性结肠炎体会[J]. 医药前沿,2013(1):336-337.
293. 李广宣. 芍药汤与痛泻药方联用治疗慢性溃疡性结肠炎的临床疗效观察[J]. 内蒙古中医药,2013,32(30):47.
294. 李敬峰,张鹏飞,姜红英. 复方黄柏液保留灌肠治疗溃疡性结肠炎的疗效[J]. 实用临床医学,2013,14(4):12-13,39.
295. 李桂娥,李小娥,许利华. 锡类散灌肠联合柳氮磺吡啶肠溶片治疗活动性溃疡性结肠炎的护理观察[J]. 吉林医学,2013,34(26):5472-5473.
296. 李玉明.溃克灵治疗炎症性肠病临床研究[J].中医学报,2013,28(12):1911-1913.
297. 杨俊波.乌梅汤加减联合西药治疗溃疡性结肠炎50例临床疗效[J].中国实用医药,2013,8(08):160-161.
298. 杨旭,王元钊,叶妮,王轶,张苏闽,方健,周惠芬.中药超声导入对溃疡性结肠炎患者的临床疗效及其细胞因子的影响[J].世界华人消化杂志,2013,21(31):3450-3455.
299. 林小芬,张伟艺,余杨,余凯云,饶泽珍.三黄汤合胃肠黏膜保护剂治疗溃疡性结肠炎37例临床观察[J].吉林医学,2013,34(26):5379-5380.
300. 樊冬梅,陶双友,赵小山.固本化瘀法对溃疡性结肠炎复发患者结肠组织表皮生长因子受体水平的影响[J].新中医,2013,45(03):48-50.
301. 武颜荣,阚存玲.固本益肠片联合美沙拉嗪片治疗中重度溃疡性结肠炎19例[J].西部中医药,2013,26(01):79-80.
302. 潘晓明.益气活血、清热利湿法治疗溃疡性结肠炎疗效观察[J].中国中医药现代远程教育,2013,11(09):29-30.
303. 王丽萍,马俊,宁晓梅.柳氮磺胺吡啶结合双料喉风散保留灌肠对溃疡性直乙状结肠炎疗效的影响[J].实用医学杂志,2013,29(12):2032-2034.
304. 王再见,李会霞,梁洁,侯朝英.血竭联合地榆对溃疡性结肠炎黏膜愈合的影响[J].北京中医药大学学报,2013,36(06):426-428.
305. 王友敏.中药保留灌肠治疗溃疡性结肠炎的临床观察及护理[J].内蒙古中医药,2013,32(05):36-37.
306. 王胜文,王晓平,桑原锋,等. 中西药结合治疗菌群失调性溃疡性结肠炎的临床疗效[J]. 中国肛肠病杂志,2013,33(10):41-43.
307. 王进.加味不换金正气散灌肠治疗溃疡性结肠炎临床疗效观察[J].辽宁中医药大学学报,2013,15(09):176-178.
308. 白明.中西医结合治疗慢性溃疡性结肠炎45例[J].河南中医,2013,33(10):1740-1741.
309. 皇甫建新,齐晶晶,黄强,潘国宏.穴位埋线联合美沙拉嗪治疗溃疡性结肠炎36例[J].河南中医,2013,33(12):2203-2204.
310. 罗海英.中药灌肠联合西药治疗溃疡性结肠炎疗效观察[J].山西中医,2013,29(06):25-26.
311. 臧贞祥.中西医结合治疗104例溃疡性结肠炎临床观察[J].亚太传统医药,2013,9(10):137-138.
312. 谢晶日,刘朝霞,许明月. 观察肠愈宁治疗溃疡性结肠炎前后肠炎症活动指数评分[C]//.第二十五届全国中西医结合消化系统疾病学术会议论文集.[出版者不详],2013:273.
313. 贺洁,彭学博.清热药保留灌肠配合西药治疗溃疡性结肠炎48例[J].陕西中医,2013,34(01):24-25.
314. 贾军峰.中西医诊断、治疗溃疡性结肠炎的疗效分析[J].贵阳中医学院学报,2013,35(02):66-67.
315. 赵中良.布地奈德、锡类散灌肠治疗溃疡性结肠炎的疗效观察[J].中国医药指南,2013,11(22):488.
316. 赵淑芳.肠炎灵联合5-氨基水杨酸(5-ASA)治疗溃疡性结肠炎观察[J].中国实用医药,2013,8(06):126-127.
317. 邹君君,朱莹,张晓江,蔡植,郭维军,张建伟,李擎虎,王宇红.溃结宁膏穴位敷贴对脾肾阳虚型溃疡性结肠炎的临床疗效及其对血清γ干扰素、白细胞介素-4的影响[J].中国中西医结合消化杂志,2013,21(06):305-307.
318. 邹琳,苑军伟,白蔷薇,金吉,毛庆琳,董宇翔.肠炎康联合美沙拉嗪治疗溃疡性结肠炎[J].中国实验方剂学杂志,2013,19(09):323-325.
319. 郑丽红,王海强,王楠楠,姚丹.脏连丸联合美沙拉嗪治疗溃疡性结肠炎疗效观察[J].中医药信息,2013,30(06):114-115.
320. 郑少康,杜刚毅,林锡芬,张宁.复方丹参注射液加吸氧联合西药治疗溃疡性结肠炎随机平行对照研究[J].实用中医内科杂志,2013,27(17):58-60.
321. 郝广清.中西医结合治疗溃疡性结肠炎55例[J].中国中医药现代远程教育,2013,11(24):62.
322. 郝琳.中医护理干预对溃疡性结肠炎治疗效果的影响[J].北方药学,2013,10(03):170-171.
323. 郭维军,朱莹,赵希.溃结宁膏穴位贴敷治疗脾肾阳虚型溃疡性结肠炎临床研究[J].中国中医药信息杂志,2013,20(07):10-12.
324. 钟贞. 针灸健脾补肾治疗溃疡性结肠炎临床随机对照试验[D].成都中医药大学,2013.
325. 钱弘泉.中西医结合治疗溃疡性结肠炎80例疗效观察[J].山东中医杂志,2013,32(04):263-264.
326. 陈东兴.中西药灌肠加柳氮磺胺吡啶口服治疗溃疡性结肠炎20例临床观察[J].基层医学论坛,2013,17(26):3497-3498.
327. 陈平.中药灌肠联合西药治疗溃疡性结肠炎随机平行对照研究[J].实用中医内科杂志,2013,27(10):105-107.
328. 陈文杰. 加味芍药汤口服联合灌肠治疗溃疡性结肠炎的疗效观察[D].南京中医药大学,2013.
329. 陈斌,刘艳红.安肠愈疡汤保留灌肠联合常规疗法治疗溃疡性结肠炎48例[J].江西中医药,2013,44(11):21-22.
330. 陈涛,叶柏.中西医结合治疗溃疡性结肠炎活动期30例[J].山东中医杂志,2013,32(06):418-419.
331. 马刚,戴伟杰.丹红注射液对溃疡性结肠炎患者CD62p的影响[J].皖南医学院学报,2013,32(06):471-473.
332. 黄更珍,贺国斌,张耀丹,陈予,廖娟,明文,张琴.美沙拉嗪联合锡类散、云南白药及地塞米松保留灌肠治疗溃疡性结肠炎[J].湖北民族学院学报(医学版),2013,30(03):31-33+36.
333. 黄磊,蔡植,朱莹,万虎.溃结宁膏穴位贴敷治疗脾肾阳虚型溃疡性结肠炎:随机对照研究[J].中国针灸,2013,33(07):577-581.
334. 黄英姿,张丽.中药疏肝健脾止痢方治疗溃疡性结肠炎患者的临床作用[J].世界华人消化杂志,2013,21(31):3445-3449.
335. 杨达,刘艾.中西医结合治疗难治性溃疡性结肠炎疗效观察[J].中西医结合实用临床急救,1998(07):12-14.
336. 王星田,赵玲.溃结丸配合思密达治疗溃疡性结肠炎34例[J].国医论坛,1998(04):31.
337. Zhou Q, Yu J, Gu S. [Clinical and experimental study on treatment of retention enema for chronic non-specific ulcerative colitis with quick-acting kuijie powder]. Zhongguo Zhong Xi Yi Jie He Za Zhi. 1999 Jul;19(7):395-8. Chinese. PMID: 11783209.
338. 宋海波,陈万强,李虎林.中西医结合治疗溃疡性结肠炎30例[J].陕西中医,1999(04):168.
339. 张兆泉,张运贵.中西医结合治疗慢性非特异性溃疡性结肠炎26例[J].河北中医,1999(04):246-247.
340. 史肃育.中西药治疗慢性溃疡性结肠炎63例疗效分析[J].时珍国医国药,2000(04):331-332.
341. 宇傲霜,王红梅,任文海.中西医结合治疗溃疡性结肠炎的疗效观察[J].现代中西医结合杂志,2000(21):2095-2096.
342. 李媛.固本益肠片等治疗溃疡性结肠炎30例临床观察[J].现代中西医结合杂志,2000(14):1341-1342.
343. 李智君,陈淑婷,何小平,张素琴.隔姜灸配合药物灌肠治疗慢性溃疡性结肠炎40例[J].山西中医,2000(01):35.
344. 李雅华.中西医结合治疗溃疡性结肠炎120例[J].广西中医学院学报,2000(02):27-28.
345. 王言飞,祝传丹,魏善和.思密达锡类散氢化考的松保留灌肠治疗溃疡性结肠炎32例分析[J].济宁医学院学报,2000(02):41.
346. 温玉玲.中西医结合治疗慢性溃疡性结肠炎42例临床体会[J].深圳中西医结合杂志,2000(03):118-119+123.
347. 田永峰,魏玮.中西医结合治疗溃疡性结肠炎40例临床观察[J].山西中医,2000(06):25-26.
348. 胡团敏,张丽婷,郑溪水. 3种方法治疗溃疡性结肠炎临床疗效观察[J]. 世界今日医学杂志,2000,001 (4):323.
349. 赵增虎,丁瑞亮,李丽荣,李成云.多因子灌肠治疗溃疡性结肠炎62例疗效观察[J].现代中西医结合杂志,2000(19):1876-1877.
350. 尹合坤,邝子良.柳氮磺胺吡啶、甲硝唑、云南白药联合灌肠治疗溃疡性结肠炎疗效观察[J].中国中西医结合消化杂志,2001(06):357-358.
351. 周益萍.中西医结合治疗溃疡性结肠炎疗效观察[J].镇江医学院学报,2001(01):83-84.
352. 宋庆江,王韶华.高压氧合锡类散灌肠治疗溃疡性结肠炎[J].浙江中西医结合杂志,2001(02):16-17.
353. 廖云峰,谷时雨.中西医结合治疗溃疡性结肠炎30例[J].湖南中医药导报,2001(06):307-308.
354. 张兆泉,韩琴,苏建华. 中医辨证用药配合思密达灌肠治疗溃疡性结肠炎40例[J]. 中国肛肠病杂志,2001,21(7):26-27.
355. 徐大龙,顾月星,李国安. 中西医结合治疗慢性溃疡性结肠炎142例[J]. 中国中西医结合消化杂志,2001,9(1):49-50.
356. 房玉梅. 复方三黄汤配合甲硝唑灌肠治疗溃疡性结肠炎临床观察[J]. 河北中医,2001,23(11):811-812.
357. 李岳山,景菊,范培菊. 中西药结合治疗溃疡性结肠炎32例分析[J]. 中国腹部疾病杂志,2001,1(1):66.
358. 杨艳军.中西医结合治疗溃疡性结肠炎[J].内蒙古中医药,2001(04):22.
359. 王洪斌.中西药结合治疗活动期溃疡性结肠炎30例[J].医药导报,2001(12):748-749.
360. 罗葵良,黄生林,蓝珍,苏兰菊,蒋义生,蓝艳春.中西药灌肠治疗溃疡性结肠炎的对比研究[J].右江民族医学院学报,2001(01):14-15.
361. 蔡凌威.致康胶囊灌肠治疗溃疡性结肠炎34例疗效观察[J].中国药业,2001(11):66-67.
362. 任志刚,汪培军,尚桂梅.锡类散复合灌肠液治疗慢性溃疡性结肠炎22例报道[J].河南职工医学院学报,2002(01):69-70.
363. 何家桐.黄芪建中汤合用柳氮磺吡啶、氢化可的松治疗溃疡性结肠炎疗效观察[J].广东医学院学报,2002(02):128.
364. 夏金荣 ,张少虹 ,杨杰.中西药联合灌肠治疗溃疡性结肠炎的疗效观察[J].临床消化病杂志,2002(01):19-21.
365. 孙向红,于新民,陈秀兰.中西医结合治疗慢性非特异性溃疡性结肠炎60例[J].陕西中医,2002(07):607-608.
366. 张仁杰.中西医结合治疗溃疡性结肠炎28例[J].湖北中医杂志,2002(08):20.
367. 施进.中西药灌肠治疗轻、中度溃疡性结肠炎疗效比较分析[J].蚌埠医学院学报,2002(06):507-508.
368. 李锦成.中西医结合治疗慢性溃疡性结肠炎84例[J].陕西中医,2002(07):609-610.
369. 税典奎,刘宏伟.中西医结合治疗溃疡性结肠炎的临床观察[J].临沂医学专科学校学报,2002(04):300-301.
370. 侯再恩.肠炎平治疗溃疡性结肠炎58例[J].新乡医学院学报,2003(05):376-377.
371. 周振辉,王秀芹,魏兆华.中西医结合治疗溃疡性结肠炎64例疗效观察[J].现代中西医结合杂志,2003(02):150-151.
372. 李林运.愈溃散保留灌肠治疗非特异性溃疡性结肠炎46例[J].陕西中医,2003(07):621-622.
373. 王小平,郑亮.中西医结合治疗溃疡性结肠炎疗效观察[J].现代中西医结合杂志,2003(04):366-367.
374. 王旭.从痈论治溃疡性结肠炎52例报告[J].甘肃中医,2003(11):22-23.
375. 王璐,李克迪,谭华. 中药脐疗辅助治疗溃疡性结肠炎的临床研究[J]. 中国肛肠病杂志,2003,23(7):9-10.
376. 蔡军峰.锡类散保留灌肠治疗溃疡性结肠炎的护理[J].实用临床医学,2003(03):122-123.
377. 贾小强,张丽娟.中西医结合治疗慢性溃疡性结肠炎临床观察[J].山西中医,2003(06):23-24.
378. 赵杰芬,刘清德,周俊琴.中西药保留灌肠治疗溃疡性结肠炎46例临床观察[J].临床荟萃,2003(01):25.
379. 邱日森.中西医结合治疗溃疡性结肠炎86例临床观察[J].海南医学,2003(09):85-86.
380. 陈劲勇,周志光,王跃平.柳氮磺吡啶加中药与单用柳氮磺吡啶在溃疡性结肠炎中的疗效比较[J].中国新药与临床杂志,2003(05):287-288.
381. 周淑琴,翟秀华,张玉洁. 中药加地塞米松灌肠治疗溃疡性结肠炎40例临床观察[J]. 中国肛肠病杂志,2004,24(7):14-15.
382. 张金炎. 中西医结合治疗溃疡性结肠炎78例[J]. 现代中西医结合杂志,2004,13(18):2427-2428.
383. 曹维宏,赵婵娟.中西医结合治疗溃疡性结肠炎40例[J].陕西中医,2004(12):1108-1109.
384. 李柏林,韩永强.复方甘草片混悬液保留灌肠治疗溃疡性结肠炎疗效观察[J].现代中西医结合杂志,2004(14):1859.
385. 杨曙东,杨清,易无庸.中西医结合治疗溃疡性结肠炎临床观察[J].湖北中医杂志,2004(09):38-39.
386. 王永福,郭春林,耿立霞.中西医结合治疗溃疡性结肠炎80例临床分析[J].内蒙古中医药,2004(02):8-9.
387. 罗先珍.中西医结合治疗慢性溃疡性结肠炎疗效观察[J].护理学杂志,2004(09):40-41.
388. 陆素琴.中西药联用治疗溃疡性结肠炎48例临床观察[J].江苏中医药,2004(11):30.
389. 陈丽英.“健脾清肠汤”结合SASP治疗UC23例临床观察[J].江苏中医药,2004(03):31-32.
390. 陈利平,王发渭,刘萍.中西医结合治疗溃疡性结肠炎65例报告[J].解放军医学杂志,2004(07):630-631.
391. 蔡春江,褚志敏,崔淑芬,陈彤君.愈疡汤灌肠治疗溃疡性结肠炎的临床观察[J].中成药,2005(11):1287-1290.
392. 伍彦.中西医结合保留灌肠治疗溃疡性结肠炎临床观察[J].四川中医,2005(01):39-40.
393. 刘文龙,初丽云,呼汉雷.中西医结合治疗溃疡性结肠炎活动期疗效观察[J].吉林医学,2005(02):199-200.
394. 宋洪梅,杨红,刘丹,陶然.保留灌肠在治疗溃疡性结肠炎32例临床观察[J].伤残医学杂志,2005(04):36-37.
395. 尚利娜.中西医结合治疗溃疡性结肠炎疗效观察[J].辽宁中医学院学报,2005(05):494-495.
396. 崔勇,张荣香.普乐拜尔、惠迪口服加锡类散灌肠三联治疗溃疡性结肠炎初步观察[J].邯郸医学高等专科学校学报,2005(06):22-23.
397. 张琳.中西药保留灌肠治疗溃疡性结肠炎的临床观察[J].海南医学,2005(07):138-139.
398. 徐建峰.中西医结合治疗溃疡性结肠炎36例临床观察[J].四川中医,2005(05):56-57.
399. 朱薇,贾满仓.清溃汤治疗溃疡性结肠炎30例[J].陕西中医,2005(05):430-431.
400. 朱迪.中西医结合治疗溃疡性结肠炎25例临床观察[J].中医药导报,2005(12):14-15.
401. 林一梅.加用中西药灌肠治疗溃疡性结肠炎36例疗效观察[J].中国全科医学,2005(17):1450.
402. 林锡芬.麦滋林、白及胶囊、甲哨唑合用保留灌肠治疗溃疡性结肠炎64例临床观察[J].国际医药卫生导报,2005(14):90-91.
403. 梅德祥.中西医结合治疗溃疡性结肠炎90例临床分析[J].山西职工医学院学报,2005(02):42.
404. 田永峰.40例溃疡性结肠炎患者的治疗与护理[J].山西职工医学院学报,2005(02):49-50.
405. 葛华阶,张建华.中西药结合治疗溃疡性结肠炎疗效观察[J].湖南中医学院学报,2005(04):46-61.
406. 蔡春江,褚志敏,崔淑芬,陈彤君.愈疡汤灌肠治疗溃疡性结肠炎的临床观察[J].中成药,2005(11):1287-1290.
407. 许杰忠,许铮.中西医结合治疗活动期溃疡性结肠炎42例疗效观察[J].新中医,2005(01):66-67.
408. 贺军,赵建霞,孟皓,陈隆典.中西医结合治疗溃疡性结肠炎临床分析[J].中医药学刊,2005(10):1919-1920.
409. 陈成活,曾雅静,黄印.痛泻要方加味联合洛赛克治疗溃疡性结肠炎[J].右江医学,2005(03):246-247.
410. 韩如英,毛学勤.中西药联合保留灌肠治疗慢性溃疡性结肠炎疗效观察[J].现代护理,2005(06):485-486.
411. 于晓红,路聪哲.美沙拉嗪口服联合锡类散保留灌肠治疗溃疡性结肠炎40例[J].中国中西医结合消化杂志,2006(06):401-402.
412. 刘华宝.中西医结合治疗慢性非特异性溃疡性结肠炎30例[J].四川中医,2006(04):46-47.
413. 吴坚芳,王良花,陈文奇.中西医结合治疗溃疡性结肠炎疗效观察[J].辽宁中医学院学报,2006(02):97.
414. 张丽.美常安与中药灌肠治疗溃疡性结肠炎[J].辽宁中医杂志,2006(08):990.
415. 彭飞,吴清.中西医结合治疗慢性溃疡性结肠炎48例临床观察[J].中医药导报,2006(06):20-21.
416. 曹永胜,陈银环.中西药保留灌肠治疗溃疡性结肠炎疗效观察[J].辽宁中医杂志,2006(08):1001-1002.
417. 李必瑾,高秀华,张爱琼,王正江,杨建梅.中西医结合治疗溃疡性结肠炎[J].内蒙古中医药,2006(06):33-34.
418. 涂立德.中西医结合治疗慢性溃疡性结肠炎34例疗效观察[J].赣南医学院学报,2006(03):423-424.
419. 王萌,白克运. 溃结祛毒合剂配合复方角菜酸酯栓保留灌肠治疗溃疡性结肠炎的临床观察[J]. 中国肛肠病杂志,2006,26(7):31-33.
420. 苏加强,黄泽林. 锡类散甲硝唑与思密达保留灌肠治疗溃疡性结肠炎78例临床观察[J]. 黑龙江医学,2006,30(9):686-687.
421. 赵开莉,罗英.中西医结合治疗慢性溃疡性结肠炎的观察及护理[J].现代医药卫生,2006(13):2028-2029.
422. 章连新,温成平.自免清合SASP结肠溶胶囊治疗溃疡性结肠炎的临床研究[J].浙江中医药大学学报,2006(05):472-473.
423. 马丽昕.中西药复合灌肠在慢性溃疡性结肠炎中的应用及护理[J].齐鲁护理杂志,2006(11):1030-1031.
424. 高彩莲.中药保留灌肠结合西药治疗溃疡性结肠炎护理[J].实用医技杂志,2006(14):2536-2537.
425. 高琛,仝霞,毕少茹.中西药灌肠治疗溃疡性结肠炎80例临床观察[J].中国厂矿医学,2006(02):169-170.
426. 刘丹,王奇艳. 黄芪注射液结合中药灌肠治疗溃疡性结肠炎的护理[J]. 现代护理,2007,13(32):3131-3132.
427. 刘利华,彭澎,陈开娟,邵华.中西医结合治疗慢性溃疡性结肠炎临床观察[J].中华中医药学刊,2007(03):633-634.
428. 刘福文,吴际萍,卢福元,蒋惠,马代敏.愈溃结汤加SASP及甲硝唑灌肠治疗UC[J].浙江中西医结合杂志,2007(11):676-677.
429. 卫忠妹,张金炎.中西医结合治疗溃疡性结肠炎80例[J].浙江中西医结合杂志,2007(10):609-610.
430. 周云祥,周玉中.利多卡因、柳氮磺胺吡啶联合中药灌肠治疗慢性溃疡性结肠炎56例疗效观察[J].河北中医,2007(03):217-218.
431. 唐庆林,张鸣青,王爱民,马桂芳,杨青平,李仙丽.经结肠治疗机联合柳氮磺胺吡啶治疗溃疡性结肠炎临床观察[J].现代医药卫生,2007(16):2397-2398.
432. 孙杰,付立芳.中西医结合治疗溃疡性结肠炎70例临床观察[J].现代医药卫生,2007(17):2618.
433. 廉晓露,李雅.中西医结合治疗溃疡性结肠炎56例[J].陕西中医,2007(05):559-560.
434. 张宪明,王天英. 中西医结合治疗溃疡性结肠炎68例疗效观察[J]. 实用医技杂志,2007,14(22):3086-3087.
435. 朱莹,袁伟建,白晓明. 穴位埋线对溃疡性结肠炎淋巴细胞凋亡调控蛋白的影响[J]. 中医杂志,2007,48(6):526-528.
436. 李世辉. 中西医结合治疗慢性非特异性溃疡性结肠炎32例临床观察[J]. 云南中医中药杂志,2007,28(3):22-22.
437. 李海,赵自星.中西医结合治疗溃疡性结肠炎临床研究[J].中国中西医结合消化杂志,2007(03):193-194.
438. 李素云,崔德广.温阳解毒化瘀联合柳氮磺胺砒啶治疗溃疡性结肠炎37例[J].亚太传统医药,2007(05):66+65.
439. 李颖,李志强,杜艳君.参附注射液治疗溃疡性结肠炎的临床疗效观察[J].吉林医学,2007(05):678-679.
440. 杜艳茹,刘启泉,王志坤,白海燕,刘晓辉,谭宝,张纨.药穴结合治疗溃疡性结肠炎的临床研究[J].四川中医,2007(03):52-53.
441. 杨云. 三联疗法治疗溃疡性结肠炎临床观察[C]//.首届国际中西医结合大肠肛门病学术论坛暨第十二届全国中西医结合大肠肛门病学术会议论文集萃.[出版者不详],2007:268-270.
442. 杨宪煌.川连止泻胶囊治疗溃疡性结肠炎30例[J].中国中西医结合消化杂志,2007(05):341-342.
443. 汤瑜,姚平.白头翁汤加减灌肠联合柳氮磺胺吡啶治疗非特异性溃疡性结肠炎[J].临床和实验医学杂志,2007(12):107.
444. 温燕,刘文辉.加味参苓白术散治疗慢性非特异性结肠炎57例临床观察[J].中医药导报,2007(04):37-38.
445. 王星田,董炜.中西医结合治疗慢性溃疡性结肠炎77例临床观察[J].江苏中医药,2007(12):34-35.
446. 王艳民.中西医结合治疗慢性溃疡性结肠炎60例[J].青岛医药卫生,2007(02):133-134.
447. 纪逢春,于海鹰.中西医结合灌肠治疗溃疡性结肠炎临床观察[J].辽宁中医杂志,2007(05):628-629.
448. 罗远汉,李平.自拟清癖整肠汤加西药治疗溃疡性结肠炎35例疗效观察[J].广西中医药,2007(05):23-24.
449. 翟建国,路江海,刘金松. 中西药合用灌肠治疗溃疡性结肠炎的疗效观察[J]. 现代保健·医学创新研究,2007,4(18):75-76.
450. 翟建国,路江海,刘金松,李粉格,王庆周,李爱然,王林现,常文青.丹香液与锡类散联合西药灌肠治疗溃疡性结肠炎疗效观察[J].社区医学杂志,2007(13):27-28.
451. 胡柱佳.中西医结合治疗溃疡性结肠炎60例疗效观察[J].临床医药实践杂志,2007(S3):916-917.
452. 荣家慧.丹参注射液和中药保留灌肠辅助治疗溃疡性结肠炎54例疗效分析[J].中国实用医药,2007(23):52-53.
453. 莫慧琴,汤艳兰. 云南白药和凝血酶交替保留灌肠在溃疡性结肠炎并出血中的应用及护理[J]. 现代护理,2007,13(20):1882-1883.
454. 董淑春. 中西医结合治疗慢性溃疡性结肠炎50例临床疗效观察[J]. 亚太传统医药,2007,3(9):49-50.
455. 蔡少峰,谢贵文,李继端. 中西医结合治疗慢性溃疡性结肠炎34例临床观察[J]. 江苏中医药,2007,39(3):33.
456. 郑刚. 中西医结合治疗溃疡性结肠炎52例[J]. 山西中医,2007,23(3):33.
457. 陈立东,刘莉.中西医结合治疗溃疡性结肠炎60例临床观察[J].中国中医急症,2007(12):1467-1468.
458. [1]韩桂华,鲍秀琦,姜威. 丹参联合柳氮磺胺吡啶治疗溃疡性结肠炎的疗效观察[C]//.国际血瘀证及活血化瘀研究学术大会—中西医结合防治循环系统疾病高层论坛论文集.[出版者不详],2007:453.
459. 项家席,项家陆. 中西药结合治疗慢性溃疡性结肠炎38例临床观察[J]. 中国肛肠病杂志,2007,27(3):42-43.
460. 齐玲芝,孙玉红,杨文颖. 丹参辅助治疗溃疡性结肠炎的临床分析[J]. 吉林中医药,2007,27(4):24-25.
461. 丁虎,王莉莉,肖劲声,李云峰,胡盼盼.瓦楞子散联合美沙拉嗪治疗溃疡性结肠炎[J].中医学报,2018,33(12):2436-2440.
462. 乔虹,娄华.耳穴埋豆加溃结膏穴位贴敷联合柳氮磺胺吡啶对溃疡性结肠炎的疗效观察[J].内蒙古医学杂志,2018,50(05):536-538.
463. 于效力.黄白兰草煎治疗脾肾阳虚型溃疡性结肠炎临床研究[J].国医论坛,2018,33(05):31-34.
464. 余水岸,卢孝能,余秀晶.白头翁汤联合布地奈德灌肠治疗远段溃疡性结肠炎活动期的效果观察[J].黑龙江中医药,2018,47(06):70-72.
465. 候兵.美沙拉嗪联合中药灌肠法治疗溃疡性结肠炎的效果探究[J].当代医药论丛,2018,16(17):143-144.
466. 侯炜炜,吴志美,许亮亮.隔药灸结合美沙拉嗪治疗溃疡性结肠炎的临床疗效及对血清免疫球蛋白、白细胞介素-17和血栓素B2的影响[J].临床和实验医学杂志,2018,17(19):2097-2101.
467. 信纪朋. 溃结一号方治疗脾肾阳虚型溃疡性结肠炎的临床研究[D].河南中医药大学,2018.
468. 冯桂英,韩志军,胡海燕,王晓丽,徐永祥.益肾通督汤治疗慢性非特异性溃疡性结肠炎脾肾阳虚证临床观察[J].河北中医,2018,40(01):94-97.
469. 刘倩.美沙拉嗪联合益阳愈溃汤治疗溃疡性结肠炎疗效分析[J].实用中医药杂志,2018,34(09):1080-1081.
470. 刘君君,吴际,何瑞华,李艳.观察中西医联合疗法对溃疡性结肠炎的治疗效果[J].中国实用医药,2018,13(16):127-129.
471. 刘学成,孙慧,张宏国,睢勇.自拟补脾泻火方联合西药治疗溃疡性结肠炎近期疗效观察[J].中国中医急症,2018,27(05):880-882.
472. 刘春雨,卢彩宝,叶秋,骆文君,李丽.健脾补肾方联合柳氮磺吡啶对慢性溃疡性结肠炎患者疗效及炎症因子影响[J].云南中医学院学报,2018,41(01):65-67+80.
473. 刘歆.中西结合治疗溃疡性结肠炎临床疗效观察[J].药品评价,2018,15(02):38-42.
474. 刘泽涛,于爱萍. 中西医结合方法治疗重症溃疡性结肠炎的效果[J]. 医学食疗与健康,2018(11):164,166.
475. 刘淑正,孟宪静,李伟,吕婕,付翠艳.补气祛湿方联合美沙拉秦缓释颗粒治疗慢性非特异性溃疡性结肠炎临床观察[J].河北中医,2018,40(07):1054-1057.
476. 卢时万. 针灸结合中西药物综合治疗慢性溃疡性结肠炎的临床疗效评价[J]. 医药前沿,2018,8(15):328.
477. 厉启芳,刘善军,景德怀,刘国霞,朱亚珍,孙冰,章洪华,于斌.基于临床疗效及Th1/Th2、Th17/Treg炎性平衡变化探讨痛泻要方治疗溃疡性结肠炎的机制[J].辽宁中医杂志,2018,45(08):1569-1572.
478. 吴辉,袁晓玲.板蓝根免煎颗粒灌肠联合美沙拉嗪治疗溃疡性结肠炎的临床研究与应用[J].中国现代药物应用,2018,12(17):206-207.
479. 周晓凤,宋亚一,王伟,曹志群.中药“灌肠方”灌肠辅助美沙拉嗪肠溶片口服治疗溃疡性结肠炎32例[J].山东医药,2018,58(39):71-74.
480. 商湘. 溃结灵Ⅳ号保留灌肠联合美沙拉嗪口服治疗大肠湿热型UC的临床观察[D].黑龙江中医药大学,2018.
481. 孙婕怡,胥冰.“健脾益气、解毒通络”方保留灌肠对溃疡性结肠炎患者血清IL-8、TNF-α含量变化及生存质量的影响[J].世界中西医结合杂志,2018,13(07):960-964.
482. 孙燕,刘涛.美沙拉嗪结合中药灌肠治疗溃疡性结肠炎临床疗效观察[J].新疆医学,2018,48(08):847-849.
483. 孙盟朝,田晶晶,崔莉红.乌梅丸治疗溃疡性结肠炎的疗效分析及对T淋巴细胞的影响[J].中国中西医结合外科杂志,2018,24(05):545-549.
484. 安平. 益气活血清肠汤治疗溃疡性结肠炎的临床观察[J]. 医学新知杂志,2018,28(z1):248-249.
485. 崔景怡,金玲,李慕然,崔纪芳,靳明星,刘艳迪.促愈祛毒方内服灌肠辅助治疗活动期溃疡性结肠炎疗效及对血清炎性细胞因子、TGF-β、MMP-1水平的影响[J].中国中医急症,2018,27(07):1263-1265.
486. 常为伟.中西医结合治疗溃疡性结肠炎临床研究[J].实用中医药杂志,2018,34(05):541-542.
487. 常为伟.健脾化湿汤联合清肠栓对脾虚湿热型溃疡性结肠炎患者生存质量的影响[J].北方药学,2018,15(07):16-17.
488. 廖衡,石立鹏,张金龙,杨德钱,谭克文,邬红霞,张燕.芍药汤加味联合臭氧直肠灌注治疗溃疡性结肠炎大肠湿热证临床研究[J].新中医,2018,50(01):53-56.
489. 张伟,杨银利,张林,林俊超,郝蕾,吴洁琼,相祎.青柏溃结汤灌肠对溃疡性结肠炎大肠湿热证患者结肠黏膜愈合及血清炎性因子水平的影响[J].新乡医学院学报,2018,35(06):474-477.
490. 张大伟. 中西医联合多途径治疗近段溃疡性结肠炎临床疗效分析[J]. 心理医生,2018,24(13):171-172.
491. 张宏,谢小强.自拟扶正通络解毒汤治疗慢性溃疡性结肠炎的临床观察[J].中医临床研究,2018,10(26):42-44.
492. 张慧田,周知然,雷云霞. 益气除湿法联合美沙拉嗪治疗溃疡性结肠炎临床观察[J]. 西部中医药,2018,31(5):87-89.
493. 张晋资,朱文宗,宋成城. 加味吴茱萸粗盐包治疗溃疡性结肠炎的临床观察[J]. 中国中西医结合消化杂志,2018,26(4):386-388.
494. 张浩彬,叶柏. 清肠化湿灌肠方保留灌肠治疗激素抵抗型重症溃疡性结肠炎疗效及对血清炎症因子的影响[J]. 现代中西医结合杂志,2018,27(17):1858-1860,1867.
495. 张爱军,朱叶珊,费亚军,费璇,郑皓,张艳君,刘润华.中药封包电磁波导入辅治溃疡性结肠炎的疗效及对血清炎性因子和免疫球蛋白的影响[J].疑难病杂志,2018,17(04):370-373.
496. 张爱军,朱叶珊,费亚军,郑皓,费璇,刘润华,吉梅,张艳君.健脾祛浊解毒中药舒适灌肠法治疗溃疡性结肠炎疗效评价[J].中医药通报,2018,17(02):54-56+64.
497. 张玉梅.健脾除湿汤联合柳氮磺吡啶治疗溃疡性结肠炎?(?脾虚湿蕴?)随机平行对照研究[J].实用中医内科杂志,2018,32(12):21-23.
498. 张肖.参苓白术颗粒联合美沙拉嗪治疗脾虚湿盛型溃疡性结肠炎16例[J].江西中医药,2018,49(06):39-41.
499. 张莉敏,周显华,郑政隆,冯阳,刘源,杜强,任燕怡,钟森.中医“伏邪”理论论治活动期溃疡性结肠炎的多中心临床研究[J].光明中医,2018,33(24):3663-3666.
500. 张雪莹,单海燕,薄淑萍.隔药灸联合五音疗法治疗溃疡性结肠炎伴焦虑抑郁疗效观察[J].上海针灸杂志,2018,37(07):733-737.
501. 张雪莹,单海燕,薄淑萍.沈氏达郁汤加减方联合五音疗法治疗溃疡性结肠炎伴焦虑抑郁[J].山东中医杂志,2018,37(10):824-826+868.
502. 朱叶珊,费亚军,费璇,郑皓,刘润华,董雪莲,吉梅,张艳君.健脾祛浊解毒法联合美沙拉嗪对溃疡性结肠炎黏膜愈合的影响[J].中国中医药现代远程教育,2018,16(14):122-124.
503. 李显峰.中药灌肠法联合美沙拉嗪治疗溃疡性结肠炎的疗效观察[J].中西医结合心血管病电子杂志,2018,6(33):170.
504. 李松.美沙拉嗪缓释片联合中药灌肠治疗溃疡性结肠炎的临床疗效探讨[J].中国民康医学,2018,30(09):75-76.
505. 李玉春,郝毅,王景琦.中药灌肠治疗溃疡性结肠炎的效果分析[J].临床医药文献电子杂志,2018,5(41):143-144.
506. 李积良,王继宁,陈凌燕,柳春生.固肠煎灌肠配合西药治疗溃疡性结肠炎50例临床分析[J].青海医药杂志,2018,48(03):61-62.
507. 李美霞. 中西医结合治疗溃疡性结肠炎临床观察[J]. 实用中医药杂志,2018,34(9):1084-1085.
508. 杜丽君.穴位埋线疗法联合口服美沙拉嗪治疗溃疡性结肠炎的护理分析[J].医学信息,2018,31(05):172-173.
509. 杨小娟,赵莹,王春玲.恒温中药灌肠治疗溃疡性结肠炎的临床应用[J].中国肛肠病杂志,2018,38(06):47-48.
510. 杨斌. 温经汤治疗溃疡性结肠炎（脾肾阳虚夹瘀证）的临床观察[D].长春中医药大学,2018.
511. 杨晓蓓,谭文婧,节阳华,谭星.补脾益肠丸联合美沙拉嗪缓释颗粒剂治疗溃疡性结肠炎[J].长春中医药大学学报,2018,34(06):1130-1133.
512. 杨继芬.美沙拉嗪结合白头翁汤灌肠用于溃疡性结肠炎治疗116例临床分析[J].影像研究与医学应用,2018,2(20):247-248.
513. 杨颖,闫文峰.参苓白术散合美沙拉嗪治疗溃疡性结肠炎临床观察[J].中国民族民间医药,2018,27(20):90-91.
514. 梁新雨,张相安.益气活血法联合美沙拉嗪治疗慢性溃疡性结肠炎疗效观察[J].国医论坛,2018,33(04):47-48.
515. 樊雅宁,李雪青,石志敏.当归拈痛汤联合美沙拉嗪治疗湿热内蕴型溃疡性结肠炎的随机对照研究[J].中国中西医结合消化杂志,2018,26(11):915-918.
516. 牟大礼.美沙拉嗪+中药汤剂治疗溃疡性结肠炎的临床效果分析[J].中外医疗,2018,37(28):166-168.
517. 王传颂.葛根芩连汤联合柳氮磺胺吡啶对溃疡性结肠炎患者炎症反应的影响[J].光明中医,2018,33(10):1465-1467.
518. 王国策. 白头翁汤加减方与美沙拉嗪联合治疗活动期溃疡性结肠炎的临床效果[J]. 医药前沿,2018,8(2):336-337.
519. 王欣,陈爱霞,王佳,王梅青.平溃健脾汤联合美沙拉嗪在溃疡性结肠炎治疗中的应用[J].山东医药,2018,58(26):44-46.
520. 王海燕,刘友伦. 复方青黛胶囊配合益生菌治疗溃疡性结膜炎疗效及对血清HSP70、GSH-Px、IL-23和SOD水平的影响[J]. 国际医药卫生导报,2018,24(9):1410-1412.
521. 王红. 中药灌肠联合口服给药治疗溃疡性结肠炎患者的临床观察[J]. 中国民康医学,2018,30(11):88-89.
522. 石罗君,李海元,黄瑞华,王晓明,黄小艺,梁莹莹,汤倩倩.壮医药线点灸配合壮医药物灌肠疗法治疗溃疡性结肠炎疗效观察及安全性评价[J].世界中西医结合杂志,2018,13(11):1581-1584+1588.
523. 石罗君,李海元,黄瑞华,王晓明,黄小艺,梁莹莹,汤倩倩.壮医药物灌肠辅助治疗溃疡性结肠炎疗效观察[J].中医临床研究,2018,10(20):46-47.
524. 秦晓纲.清肠化湿灌肠方直肠滴入治疗激素抵抗型重症溃疡性结肠炎的疗效观察[J].实用临床医药杂志,2018,22(11):62-65.
525. 管亚丽,张杰.加味白术黄芪汤联合西药治疗溃疡性结肠炎34例[J].中医研究,2018,31(09):17-19.
526. 罗红.健脾清肠汤联合双歧杆菌三联活菌胶囊治疗慢性非特异性溃疡性结肠炎临床观察[J].河北中医,2018,40(09):1363-1366.
527. 罗超兰,胡正昌,杨向东.龙胆泻肝汤联合美沙拉嗪治疗湿热下注型溃疡性结肠炎的临床观察[J].中国中医药科技,2018,25(05):696-697.
528. 罗青华,袁恩.少腹逐瘀汤内服联合康复新液灌肠治疗血瘀肠络型溃疡性结肠炎的疗效及对APTT和FIB的影响[J].现代中西医结合杂志,2018,27(28):3141-3144.
529. 罗瑞娟,杨玉刚,张艳红.自拟愈疡汤保留灌肠联合美沙拉嗪口服治疗溃疡性结肠炎的疗效[J].保健医学研究与实践,2018,15(04):36-39.
530. 腾阳,汤绍迁.四白黄连愈合散联合密波电针治疗溃疡性结肠炎50例疗效观察[J].湖南中医杂志,2018,34(04):62-64.
531. 董雪莲,常玉洁,吴艳红,赵凤玲,张晓蕾.不同腧穴配伍灸法联合美沙拉嗪治疗溃疡性结肠炎的平行对照研究[J].世界中西医结合杂志,2018,13(05):649-652.
532. 薛兰花.白头翁汤与美沙拉嗪联合治疗溃疡性结肠炎疗效观察[J].内蒙古中医药,2018,37(05):55-56.
533. 薛雾松,刘仍海.康复新液对老年UC患者的治疗效果及对血清MDA、SOD、hs-CRP水平的影响[J].解放军预防医学杂志,2018,36(10):1248-1250+1254.
534. 袁玲.美沙拉嗪联合中药灌肠疗法治疗溃疡性结肠炎的效果观察[J].当代医药论丛,2018,16(21):121-122.
535. 袁联华.中西医结合治疗溃疡性结肠炎疗效观察[J].实用中医药杂志,2018,34(06):694-695.
536. 许秀芬,何泮,李桂云,等. 中药灌肠治疗溃疡性结肠炎的临床观察[J]. 光明中医,2018,33(1):57-58,104.
537. 谢伟昌,姜小艳,张竞超,李健,郭绍举,黄明河,黄彬.基于“微生物-脑-肠轴”观察理肠汤对脾虚湿困型溃疡性结肠炎患者的作用[J].中国中西医结合消化杂志,2018,26(12):1013-1017.
538. 谢静,杨雪,赵鲁卿,沈洪,赵文霞,唐志鹏,谢胜,张声生.健脾温肾清化方治疗溃疡性结肠炎近期疗效观察[J].北京中医药,2018,37(07):602-605.
539. 赵崧,王晓骁,张露,沈洪.清肠化湿方联合美沙拉嗪对溃疡性结肠炎患者粪便钙卫蛋白的影响[J].河北医学,2018,24(03):445-448.
540. 赵悦. 美沙拉嗪肠溶片联合溃结灵1号灌肠治疗溃疡性结肠炎疗效观察[D].河北北方学院,2018.
541. 郭红,刘蔚,曾娟,钟毅.中药联合臭氧灌肠对溃疡性结肠炎患者生存质量及TNF-α、IL-6、hs-CRP的影响[J].中医药导报,2018,24(22):93-95+100.
542. 郭育慧.中药内外合治溃疡性结肠炎42例临床研究[J].名医,2018(04):56.
543. 郭逢源.加味芍药汤方与美沙拉嗪联用对溃疡性结肠炎患者的疗效及其对血清超敏C-反应蛋白水平的影响[J].抗感染药学,2018,15(07):1156-1158.
544. 钟元帅. 安肠汤保留灌肠联合口服治疗轻、中度溃疡性结肠炎的临床研究[D].广西中医药大学,2018.
545. 陈世芬,李敏莹,纪树亮,孙治中,孙伟鹏,任悦怡,李宗瑶.针灸结合中药保留灌肠治疗溃疡性结肠炎的临床观察[J].中国民族民间医药,2018,27(23):103-106.
546. 陈俊余,王剑,程丽敏.穴位贴敷配合药物治疗湿热内蕴型溃疡性结肠炎疗效观察[J].上海针灸杂志,2018,37(10):1144-1147.
547. 陈倩,孙玲. 中西药物保留灌肠联合针灸治疗溃疡性结肠炎的护理效果探究[J]. 结直肠肛门外科,2018(s2).
548. 陈建林,陈锦锋,韩宇斌,等. 芍药汤保留灌肠对湿热型溃疡性结肠炎患者炎性因子的影响[J]. 中国中西医结合消化杂志,2018,26(11):938-940.
549. 陈旭侠,梁三红,马费强. 丹参注射液联合美沙拉嗪对轻中度活动期溃疡性结肠炎患者血清HIF-1α、炎症细胞因子水平的影响[J]. 上海中医药杂志,2018,52(7):55-58.
550. 韩婷,郭喜军,张晓艳,丁晓坤,师虹艳,霍耐月,闫一慧.加味白头翁汤为主治疗溃疡性结肠炎临床疗效及对血清炎症因子水平的影响[J].河北中医药学报,2018,33(06):16-19.
551. 顾振.白头翁汤加减联合美沙拉嗪治疗湿热型溃疡性结肠炎疗效分析[J].中医临床研究,2018,10(10):105-106.
552. 顾锡桂,蒲应香,邢成文.针灸配合中药灌肠治疗溃疡性结肠炎近远期疗效及安全性分析[J].河北医药,2018,40(04):518-522.
553. 冯桂英,韩志军,胡海燕,王晓丽,徐永祥.益肾通督汤治疗慢性非特异性溃疡性结肠炎脾肾阳虚证临床观察[J].河北中医,2018,40(01):94-97.
554. 马淑彦,焦聪敏.中西医结合治疗炎症性肠病的疗效及免疫指标改变的研究分析[J].临床医药文献电子杂志,2018,5(98):49.
555. 黄琪,雷银福,荆媛. 中药联合美沙拉嗪肠溶片口服治疗中重度活动期 溃疡性结肠炎的随机对照临床研究[J]. 心理医生,2018,24(29):140-141.
556. 龚雪瑶. 标本兼顾治疗老年溃疡性结肠炎及积分法评定疗效的临床研究[D]. 湖北:湖北中医药大学,2018.
557. 丁少华. 清肠化湿颗粒治疗活动期溃疡性结肠炎湿热内蕴证的临床观察[D].广西中医药大学,2019.
558. 乔鑫,李丹丹,沈蕾蕾.清热解毒生肌汤保留灌肠治疗活动期溃疡性结肠炎的临床观察[J].中国中医急症,2019,28(11):2022-2025.
559. 俞媛,李晨.芍黄安肠汤对溃疡性结肠炎患者肠道菌群及氧化应激的影响[J].北京中医药,2019,38(09):927-930.
560. 俞媛,李晨.芍黄安肠汤治疗溃疡性结肠炎的临床疗效及对免疫功能的影响[J].现代中西医结合杂志,2019,28(01):30-33.
561. 刘伙军.中西医结合治疗溃疡性结肠炎60例临床分析[J].心理月刊,2019,14(18):192.
562. 刘兴华.中西医结合治疗慢性溃疡性结肠炎临床疗效观察[J].中国实用医药,2019,14(28):92-93.
563. 刘厚强,孙明雷. 中西医结合治疗溃疡性结肠炎临床观察[J]. 中国保健营养,2019,29(10):83.
564. 刘弼,陈萍,肖鹏. 四逆汤加减联合美沙拉嗪栓治疗溃疡性结肠炎64例临床观察[J]. 湖南中医杂志,2019,35(6):46-47.
565. 刘朝峰. 中西医结合治疗溃疡性结肠炎的临床疗效分析[J]. 中国肛肠病杂志,2019,39(11):30-31.
566. 刘芳,雷娜,唐学贵. 当归芍药散合槐花散加减治疗溃疡性结肠炎活动期大肠湿热证的临床观察[J]. 中国实验方剂学杂志,2019,25(20):82-87.
567. 刘超,杨静,朱凤池,李晓琳,马靖.丹参注射液联合复合乳酸菌胶囊治疗溃疡性结肠炎的疗效评估[J].河北医药,2019,41(13):2012-2014+2018.
568. 卢本银,史仁杰.美沙拉嗪联合附子理中丸治疗脾肾阳虚证溃疡性结肠炎的效果及对血清HIF-1α、SOCS-3水平的影响[J].中国现代医生,2019,57(16):30-33.
569. 叶惠珍,陈陶甫,徐权胜.安肠愈疡汤联合美沙拉嗪治疗溃疡性结肠炎的疗效及对血清炎症因子水平的影响[J].云南中医中药杂志,2019,40(04):58-59.
570. 周艳阳,陈立平.清肠愈疡汤联合美沙拉嗪治疗溃疡性结肠炎的效果观察[J].中国民康医学,2019,31(24):88-90.
571. 姚轶,耿昌海,张国宝.固肠愈疡汤联合美沙拉嗪治疗溃疡性结肠炎临床观察及对肠黏膜屏障功能的影响[J].四川中医,2019,37(10):121-124.
572. 姜小艳,谢伟昌,周大桥,李健,郭绍举,黄彬.理肠汤对脾虚湿困型溃疡性结肠炎患者肠道微生态、代谢产物及炎症因子的影响[J].新中医,2019,51(02):142-146.
573. 姜璐,郭良清,吴波,姜建国.愈溃凉血颗粒联合美沙拉嗪肠溶片治疗大肠湿热型溃疡性结肠炎临床观察[J].山东医药,2019,59(31):80-82.
574. 孙寿峰.美沙拉嗪联合丹参注射液治疗溃疡性结肠炎的临床分析[J].中国医药指南,2019,17(04):60-61.
575. 孙龙娥.激素难治性溃疡性结肠炎临床特点与中西医结合治疗疗效观察[J].世界最新医学信息文摘,2019,19(09):140-141.
576. 季芳,鞠宝兆,高文艳.基于浊毒理论治疗溃疡性结肠炎的疗效及对血清脑肠肽、炎症因子的影响[J].海南医学院学报,2019,25(20):1557-1561.
577. 宋东旭,何洪芹,张文岭,李亚培.糜蛋白酶联合锡类散灌肠治疗溃疡性结肠炎的疗效及对患者炎症反应和免疫功能的影响[J].中国中西医结合外科杂志,2019,25(06):968-972.
578. 庞慧明.八髎穴导气针法联合美沙拉嗪治疗活动期溃疡性结肠炎临床研究[J].新中医,2019,51(11):209-213.
579. 张亚利,郭倩,郑烈,戴彦成,唐志鹏.健脾清肠方治疗脾虚湿热型激素依赖溃疡性结肠炎患者的临床疗效[J].中国实验方剂学杂志,2019,25(10):69-73.
580. 张双喜,宋晓锋,张相安,安永康.薏苡附子败酱散联合美沙拉嗪对溃疡性结肠炎患者的临床疗效[J].中成药,2019,41(11):2642-2646.
581. 张团结.白头翁汤加减灌肠方对急性期溃疡性结肠炎患者炎症反应的影响[J].临床医学,2019,39(08):119-121.
582. 张庆东,袁泉良,孙晓.化湿通络方灌肠联合美沙拉嗪肠溶片治疗溃疡性结肠炎临床研究[J].新中医,2019,51(12):115-117.
583. 张敏,鲁天瑜,王立群.仙白助运汤联合美沙拉嗪治疗肝郁脾虚型溃疡性结肠炎疗效观察[J].西部中医药,2019,32(11):95-97.
584. 张晓玉,郭喜军,王雨,谢卜超,默雪梅.化浊解毒消溃煎对浊毒内蕴型溃疡性结肠炎临床干预的影响[J].河北中医药学报,2019,34(05):26-29.
585. 张永华,冯辉,姜浩.清热祛湿协定方保留灌肠对溃疡性结肠炎(胃肠湿热)活动期疗效及血清炎性因子的影响[J].宁夏医学杂志,2019,41(02):184-187.
586. 张淑青,戚明.冰硼愈疡汤联合太宁复方角菜酸酯栓治疗左半结肠型急性期溃疡性结肠炎的研究[J].中国中西医结合消化杂志,2019,27(01):16-19+24.
587. 张炜娟.乌梅丸加味联合美沙拉嗪治疗寒热错杂型溃疡性结肠炎临床观察[J].光明中医,2019,34(12):1896-1898.
588. 张烨,李春涛,鲁蕾,刘亮.复方黄柏液灌肠联合美沙拉嗪对溃疡性结肠炎患者黏附因子的影响及疗效分析[J].临床消化病杂志,2019,31(05):312-315.
589. 张磊,王会丽.柳氮磺胺吡啶联合中药灌肠在治疗溃疡性结肠炎的临床疗效[J].中国合理用药探索,2019,16(11):163-165.
590. 张雪莹,单海燕,薄淑萍.隔药灸神阙八阵穴联合角调五音疗法治疗溃疡性结肠炎伴焦虑抑郁状态效果观察[J].天津中医药大学学报,2019,38(01):38-41.
591. 张霓.中西药合用治疗溃疡性结肠炎临床观察[J].实用中医药杂志,2019,35(01):49-50.
592. 徐佳萍,马朝群.白头翁汤灌肠治疗溃疡性结肠炎患者的疗效及对T细胞亚群的影响[J].南京中医药大学学报,2019,35(01):29-31+62.
593. 徐训贞,叶俊玲,林晋濠,王美蓉,王立杰,刘圣活.理中固肠汤治疗溃疡性结肠炎的疗效及机理[J].中国继续医学教育,2019,11(24):135-138.
594. 战晶玉,房莹莹,于卓,张雅丽.连草泻痢胶囊对活动期溃疡性结肠炎患者血清炎性因子及肠道黏膜屏障的影响[J].中国中西医结合消化杂志,2019,27(07):509-513+519.
595. 朱幼姗.丹参注射液联合美沙拉嗪对溃疡性结肠炎患者的治疗作用探讨[J].黑龙江中医药,2019,48(02):249-250.
596. 朱成占,张高伟,张勇. 美沙拉嗪栓联合自拟解毒凉血汤灌肠治疗直肠型溃疡性结肠炎患者临床疗效[J]. 国际医药卫生导报,2019,25(14):2345-2347.
597. 朱成慧,吕冠华,孙希良,曹玺,王丽丹,贾金玲,都嵬,张晓菲.中药溃结康丸治疗急性期溃疡性结肠炎(脾虚湿蕴证)临床效果观察及对血清炎症因子影响研究[J].辽宁中医药大学学报,2019,21(05):114-117.
598. 朱成慧,吕冠华,孙希良,曹玺,王丽丹,贾金玲,都嵬,张晓菲.溃结康丸联合美沙拉嗪缓释片治疗脾虚湿蕴型慢性非特异性溃疡性结肠炎的临床疗效及对患者血清白细胞介素8、肿瘤坏死因子α的影响[J].河北中医,2019,41(01):26-30+35.
599. 权春分,邵素菊.邵氏“五针法”治疗活动期溃疡性结肠炎临床观察[J].上海针灸杂志,2019,38(02):160-163.
600. 李仁军.溃疡性结肠炎中西医治疗优势[J].心理月刊,2019,14(02):124-125.
601. 李正军,王争艳.肠炎3号灌肠方联合西药治疗溃疡性结肠炎临床观察[J].光明中医,2019,34(23):3584-3586.
602. 李秋梅,田志颖.丹参酮ⅡA注射液联合美沙拉嗪肠溶片治疗溃疡性结肠炎患者的临床研究[J].中国临床药理学杂志,2019,35(14):1425-1427.
603. 李薇,高奎亮,李吉彦.冰及地榆汤灌肠治疗溃疡性结肠炎(左半结肠)临床研究[J].辽宁中医药大学学报,2019,21(03):25-28.
604. 李金玲.美沙拉嗪加半夏泻心汤加减联合中药保留灌肠治疗溃疡性结肠炎的效果[J].临床医学研究与实践,2019,4(01):120-121.
605. 杨杰,姜小艳,李健.理肠汤结合溃疡灵灌肠治疗脾虚湿困型溃疡性结肠炎40例[J].陕西中医药大学学报,2019,42(03):108-111.
606. 杨玉刚.甘草泻心汤对溃疡性结肠炎炎症状况及中医证候改善效果[J].中医药临床杂志,2019,31(09):1760-1762.
607. 杨薇薇.SASP加中药保留灌肠治疗溃疡性结肠炎64例[J].西部中医药,2019,32(01):102-105.
608. 杨记康.芍药汤加减保留灌肠联合艾灸治疗溃疡性结肠炎30例临床观察[J].中国民族民间医药,2019,28(03):106-108.
609. 林丹,许少华.复方苦参汤治疗慢性溃疡性结肠炎的临床疗效及对炎症因子的影响[J].慢性病学杂志,2019,20(07):1044-1046.
610. 毕夏.黄连木香配伍治疗溃疡性结肠炎临床疗效及机制研究[J].辽宁中医药大学学报,2019,21(05):187-190.
611. 沈洪,朱磊,胡乃中,孙宏普,王韶峰,孟宪梅,冯培民,张素云,任顺平,吴洁琼,胡阳黔,赵百岁,陈文习,张苏闽,王敏,田耀洲,肖建国,王琦,杨小军.虎地肠溶胶囊联合美沙拉秦肠溶片治疗活动期溃疡性结肠炎多中心、随机对照、双盲双模拟的临床研究[J].中国中西医结合杂志,2019,39(11):1326-1331.
612. 沈群,郑云硉,陆菁.隔附子饼灸联合美沙拉嗪治疗湿热型溃疡性结肠炎疗效观察[J].上海针灸杂志,2019,38(04):374-377.
613. 洪敏,陈曦.自拟溃速康汤联合西药对糖皮质激素抵抗型重度溃疡性结肠炎患者IL-4/IFN-γ失衡、血栓前状态的影响[J].现代中西医结合杂志,2019,28(29):3223-3227.
614. 潘英.中医内外合治对溃疡性结肠炎患者Th1/Th2细胞因子含量、血清胰岛素样生长因子-1、单核细胞趋化蛋白-1、高迁移率族蛋白-1水平的影响[J].环球中医药,2019,12(11):1743-1746.
615. 焦英伟,刘强,余兆惠.复方苦参结肠溶胶囊联合美沙拉嗪对改善溃疡性结肠炎患者生活质量的临床研究[J].山西医药杂志,2019,48(23):2932-2934.
616. 王丽丽,杨杰,张丽曼,王书奇,赵鹏飞,尹晓辉.理气健脾化瘀清肠法联合美沙拉嗪治疗活动期溃疡性结肠炎疗效观察[J].河北中医,2019,41(11):1680-1684+1728.
617. 王亚敏,安桂叶,宋艳琦,刘宝国,霍永利,李佃贵.翁连解毒汤治疗浊毒内蕴型溃疡性结肠炎临床疗效研究[J].河北中医药学报,2019,34(01):9-12.
618. 王会录,王孝郎.黄芩汤加味辅助美沙拉嗪治疗溃疡性结肠炎39例[J].现代中医药,2019,39(03):56-58.
619. 王凯,韩晓丽,马炳旭,刘利萍.解毒化浊促愈汤联合美沙拉嗪治疗溃疡性结肠炎80例疗效观察[J].中国肛肠病杂志,2019,39(09):27-28.
620. 王妤.美沙拉嗪联合补中益气汤治疗慢性溃疡性直肠炎的临床效果观察[J].结直肠肛门外科,2019,25(01):91-95.
621. 王小星,姚杰,姚华,高天妤,孙学东.白头翁汤加减治疗溃疡性结肠炎68例疗效观察[J].四川中医,2019,37(07):122-124.
622. 王小红,高斌.探讨健脾合剂联合美沙拉嗪治疗溃疡性结肠炎脾虚湿蕴型86例临床特征[J].中外医疗,2019,38(31):163-165.
623. 王峰,景姗,曹红艳.“健脾调肝化滞方”联合美沙拉嗪治疗溃疡性结肠炎合并抑郁30例临床研究[J].江苏中医药,2019,51(12):40-43.
624. 王晓岩,亚东,李玉春,王丽.芪芍颗粒联合美沙拉嗪治疗溃疡性结肠炎临床疗效观察[J].中国社区医师,2019,35(04):124-126.
625. 王添花,刘小聪,张检平,高满红,刘国红,肖振华.一贯煎治疗肝气郁滞型慢性溃
626. 王琼,朱晗婷,吴国荣,陈文.中西医结合治疗溃疡性结肠炎的疗效及对凝血和炎性因子水平的影响[J].海南医学院学报,2019,25(01):5-8.
627. 王瑶. 中药联合奥沙拉嗪钠胶囊治疗溃疡性结肠炎（活动期）的临床疗效探讨[D].湖北中医药大学,2019.
628. 王禾,雷天能.姜黄水煎剂灌肠联合英夫利昔单抗治疗难治性溃疡性结肠炎疗效观察[J].中国中西医结合消化杂志,2019,27(03):219-223.
629. 王紫婷.中医内外合治溃疡性结肠炎效果观察[J].临床合理用药杂志,2019,12(01):89-90.
630. 王丽丽,杨杰,张丽曼,王书奇,赵鹏飞,尹晓辉.理气健脾化瘀清肠法联合美沙拉嗪治疗活动期溃疡性结肠炎疗效观察[J].河北中医,2019,41(11):1680-1684+1728.
631. 田由京,陈兴超,张浩,李军,李合,张小丽.参苓白术颗粒联合美沙拉嗪对溃疡性结肠炎患者Th17/Treg细胞平衡的影响以及疗效分析[J].中国中西医结合消化杂志,2019,27(09):703-706.
632. 卢本银,史仁杰.美沙拉嗪联合附子理中丸治疗脾肾阳虚证溃疡性结肠炎的效果及对血清HIF-1α、SOCS-3水平的影响[J].中国现代医生,2019,57(16):30-33.
633. 肖波.益生菌、康复新液联合美沙拉嗪三联疗法治疗UC疗效观察[J].黑龙江中医药,2019,48(06):7-8.
634. 胡浩. 中药益气清肠汤治疗溃疡性结肠炎的疗效分析[J]. 现代消化及介入诊疗,2019(A02):2605-2606.
635. 胡裕耀,柯一帆,马芳笑,等. 乌梅汤联合美沙拉嗪治疗溃疡性结肠炎疗效及对炎性因子的影响[J]. 浙江临床医学,2019,21(11):1486-1487.
636. 范永强,张瑞芳,陈红霞,陈林,陈朝晖.白头翁汤加减治疗大肠湿热型溃疡性结肠炎疗效观察[J].中国中西医结合外科杂志,2019,25(04):474-477.
637. 谢梦达,彭旗. 美沙拉嗪口服联合中药灌肠治疗溃疡性结肠炎的疗效[J]. 母婴世界,2019(23):143.
638. 赖良彬,李观庆,林兆辉,车伟军,曹洪铭,曾芙蓉.柳氮磺吡啶肠溶片结合毫火针治疗溃疡性结肠炎临床观察[J].实用中医药杂志,2019,35(04):443-444.
639. 赵文文,贾占波,王庆峰,刘春志,吴东宁,蒋亦昕,李庆刚,聂艳,于边芳.脐灸疗法对溃疡性结肠炎患者BTNL2-HLA信号通路影响机制的生物信息学研究[J].中国中医药信息杂志,2019,26(08):18-23.
640. 赵红莉,闫燕,杨会,王双妮,汪永华,符翠,谢丹红.甘草泻心汤联合美沙拉嗪治疗对溃疡性结肠炎(寒热错杂证)患者中医证候积分、肝功能指标及不良反应的影响[J].四川中医,2019,37(02):113-115.
641. 赵芳超.芍药汤联合美沙拉嗪治疗溃疡性结肠炎疗效观察[J].实用中医药杂志,2019,35(03):311-312.
642. 邢一凡,柳越冬.优化溃结方治疗慢性持续性溃疡性结肠炎活动期的疗效观察[J].江西中医药,2019,50(04):57-59.
643. 郑伟伟,王嘉嘉.清肠止痢汤和艾灸辅助治疗溃疡性结肠炎的效果及对炎症因子的影响[J].中国临床药理学与治疗学,2019,24(02):198-204.
644. 郭根军,丁庆学.调肠解凝汤联合西药治疗肠道湿热型溃疡性结肠炎的临床观察[J].中国民间疗法,2019,27(10):66-67+77.
645. 钱卫珍,张卫峰.参苓白术散联合美沙拉嗪对溃疡性结肠炎儿童和青少年炎症因子与免疫功能的影响[J].医药导报,2019,38(05):584-588.
646. 闫成秋,包晗,刘屹,杨铁峥.中药塌渍治疗脾肾阳虚型溃疡性结肠炎36例[J].中国中医药现代远程教育,2019,17(05):54-56.
647. 阎玲.黄芩汤加减联合美沙拉嗪治疗溃疡性结肠炎临床观察[J].四川中医,2019,37(05):101-103.
648. 陈丽明.甘草泻心汤联合西药治疗溃疡性结肠炎30例[J].江西中医药,2019,50(03):49-50.
649. 陈俊余. 穴位贴敷联合美沙拉嗪口服治疗大肠湿热型溃疡性结肠炎的临床观察[D].黑龙江中医药大学,2019.
650. 陈华,楼颂羔,张水法.云南白药胶囊联合西药治疗溃疡性结肠炎血瘀肠络证临床研究[J].新中医,2019,51(06):148-150.
651. 陈启龙,费素娟,张易.白头翁汤灌肠联合口服美沙拉嗪对溃疡性结肠炎的疗效及其机制的研究[J].中国校医,2019,33(12):887-889+913.
652. 陈婷. 芪仙薏酱汤治疗脾虚湿蕴型溃疡性结肠炎的临床研究[D].山东中医药大学,2019.
653. 陈文竞,郭书娟,张瑞.白头翁汤加味保留灌肠治疗溃疡性结肠炎中医护理[J].光明中医,2019,34(07):1122-1123.
654. 章金钟.加味附子理中汤应用于脾肾阳虚型溃疡性结肠炎治疗的有效性分析[J].中国实用医药,2019,14(03):124-125.
655. 马乾章,李云,丁原全.白头翁汤加味联合美沙拉嗪缓释片治疗热毒炽盛型溃疡性结肠炎的临床疗效观察[J].海南医学院学报,2019,25(12):918-922.
656. 马天维,张红梅,庄晓惠,张兆美.复方黄柏液保留灌肠联合美沙拉嗪口服治疗溃疡性结肠炎的临床效果[J].中国当代医药,2019,26(01):176-179.
657. 高俊.美沙拉嗪三联用药及云南白药保留灌肠治疗E_2型活动期溃疡性结肠炎[J].海峡药学,2019,31(02):153-155.
658. 黄亮.半夏泻心汤加减治疗溃疡性结肠炎临床观察[J].光明中医,2019,34(02):199-201.
659. 黎琮毅,赵海燕,林才志,陈良荣,李桂贤,胡乃强.柴芍六君颗粒配合经肠镜植入益生菌治疗溃疡性结肠炎临床观察[J].山西中医,2019,35(05):9-13.
660. 丛龙玲,吕永慧,姚嘉茵,吴宇金,詹原泉.肠炎清合剂治疗慢性复发型溃疡性结肠炎大肠湿热证的临床观察[J].中国实验方剂学杂志,2020,26(04):120-125.
661. 于游.益脾理肠汤治疗活动期溃疡性结肠炎临床观察[J].中国中医药现代远程教育,2020,18(17):93-95.
662. 于福德.自拟中药灌肠方联合美沙拉嗪治疗溃疡性结肠炎（湿热蕴结）患者的疗效[J].中国药物经济学,2020,15(01):75-78.
663. 付荣华,王斌,郑勇,赵恩春.怡情止泻汤联合美沙拉嗪治疗肝郁脾虚型溃疡性结肠炎的可行性研究[J].湖南中医药大学学报,2020(S02):0030-0031.
664. 俞媛,王卿华,陈媛洁,李晨,张美云.痛泻要方对溃疡性结肠炎患者中医证候及肠道菌群的影响[J].湖南中医杂志,2020,36(12):12-14.
665. 修浩,高健,陆顺.真人养脏汤加减联合艾灸治疗溃疡性结肠炎脾肾阳虚证临床研究[J].河北中医,2020,42(05):700-704.
666. 刘亦阳. 自拟中药口服联合臭氧水灌肠治疗肝郁型溃疡性结肠炎临床疗效观察[D].山东中医药大学,2020.
667. 刘俊红,杨会举,雷丽亚. 槐榆炭方保留灌肠治疗溃疡性结肠炎临床观察[J]. 河南中医,2020,40(3):427-430.
668. 刘俊红,雷丽亚,杨会举. 消溃方治疗溃疡性直肠炎[J]. 中医学报,2020,35(2):407-411.
669. 刘平. 白术芍药散联合美沙拉嗪肠溶片治疗溃疡性结肠炎的临床疗效分析[J]. 中国肛肠病杂志,2020,40(8):31-32.
670. 刘志颖,张会转. 葛根芩连汤辅助治疗溃疡性结肠炎的临床疗效观察[J]. 首都食品与医药,2020,27(15):193.
671. 刘昊,霍永利,侯姿蕾,宋艳琦,李佃贵.翁连解毒汤联合美沙拉嗪治疗溃疡性结肠炎的临床研究[J].中国中西医结合消化杂志,2020,28(06):413-416+422.
672. 刘洋.香砂六君子汤加味联合美沙拉嗪治疗溃疡性结肠炎临床观察[J].实用中医药杂志,2020,36(06):766-767.
673. 刘笑彤. 槐花芍药散治疗湿热型溃疡性结肠炎的临床观察[D].山东中医药大学,2020.
674. 刘芳,孔鹏飞,郑和平.中药超声导入治疗溃疡性结肠炎的疗效[J].世界中医药,2020,15(14):2134-2137+2141.
675. 刘财堂.四君子汤加三七对脾虚湿盛型溃疡性结肠炎疗效及核周型抗中性粒细胞胞浆抗体水平的影响[J].中医临床研究,2020,12(29):68-70.
676. 刘远成,李永海,吕文辉,张承岳,张劲远,张娟,叶伟明.痛泻要方辅助治疗轻中度肝郁脾虚型溃疡性结肠炎直肠型合并焦虑状态临床研究[J].中医药临床杂志,2020,32(10):1914-1918.
677. 司马彦.柳氮磺吡啶配合半夏泻心汤治疗溃疡性结肠炎50例疗效观察[J].中国肛肠病杂志,2020,40(01):32-33.
678. 吕九娣.平溃健脾汤联合美沙拉嗪口服治疗溃疡性结肠炎的临床疗效[J].中国肛肠病杂志,2020,40(02):28-29.
679. 吴霞,龙再菊.自拟愈溃汤联合美沙拉嗪治疗溃疡性结肠炎疗效及对肠道黏膜屏障功能、氧化应激指标和血管内皮细胞功能的影响[J].现代中西医结合杂志,2020,29(10):1078-1081.
680. 周玲玲.白头翁汤在溃疡性结肠炎患者中的疗效观察及对炎症因子的影响研究[J].药品评价,2020,17(08):10-11+51.
681. 夏志勇,孟红军.中药保留灌肠联合美沙拉嗪治疗溃疡性结肠炎临床分析[J].中国中医药现代远程教育,2020,18(06):111-113.
682. 姜爱雯,杜佩珊,蒲洁琨,汤建华,刘晓明,张鹤鸣.葛根地榆紫草汤组方治疗溃疡性结肠炎的疗效及对炎症因子的影响[J].中国药物与临床,2020,20(03):341-343.
683. 姜璐,吴波,郭良清,曹志群.愈溃凉血颗粒联合美沙拉嗪对溃疡性结肠炎患者疗效及肠道屏障功能影响[J].辽宁中医药大学学报,2020,22(04):139-142.
684. 孙建新,张相安.基于“寒毒”理论探讨温阳益气解毒方治疗溃疡性结肠炎临床研究[J].新中医,2020,52(02):72-75.
685. 安桂叶,谢卜超,王亚敏,候姿蕾,刘昊,霍永利,李佃贵.翁连解毒汤对浊毒内蕴型溃疡性结肠炎患者炎症因子影响[J].时珍国医国药,2020,31(03):650-652.
686. 庄伟,马永刚.虎地肠溶胶囊结合二丙酸倍氯米松灌肠治疗活动期溃疡性结肠炎的疗效及机制分析[J].世界华人消化杂志,2020,28(13):532-537.
687. 张天彬. 健脾活血解毒颗粒联合美沙拉嗪肠溶片治疗虚实夹杂证轻度活动期溃疡性结肠炎的临床观察[D].广西中医药大学,2020.
688. 张宁,周晓丽,刘帅.加味葛根芩连汤联合常规治疗对重度湿热型溃疡性结肠炎患者的临床疗效[J].中成药,2020,42(02):351-355.
689. 张彩凤,张超群,杜学芳,夏永华,董良鹏,郭晓鹤,张利利,姬娟娟,韩宇.芪倍合剂联合美沙拉嗪治疗活动期溃疡性结肠炎的临床研究[J].现代药物与临床,2020,35(04):673-677.
690. 张微微,闫丽华,马晓晴,张寒煜.清溃愈肠饮佐治溃疡性结肠炎疗效及机制探讨[J].四川中医,2020,38(07):130-133.
691. 张艳君,常玉洁,张冬冬,葛时序,王巧玲.基于“土枢四象、一气周流”运用调脾胃升降温针法治疗溃疡性结肠炎疗效观察[J].现代中西医结合杂志,2020,29(35):3901-3906.
692. 张静文.健脾止泻汤联合美沙拉嗪治疗溃疡性结肠炎54例临床研究[J].新中医,2020,52(06):65-67.
693. 彭颖. 复方苦参汤联合美沙拉嗪肠溶片治疗溃疡性结肠炎的临床观察[J]. 世界最新医学信息文摘（连续型电子期刊）,2020,20(41):169-170.
694. 徐佳萍,马朝群.柴芍六君汤治疗肝郁脾虚型溃疡性结肠炎的疗效观察[J].中国现代医生,2020,58(15):158-161.
695. 徐琛.愈疡汤保留灌肠+美沙拉嗪栓塞肛对大肠湿热型直肠型溃疡性结肠炎症状改善及GIQLI评分的影响[J].江西中医药大学学报,2020,32(05):61-63.
696. 徐迪宇.溃疡性结肠炎治疗中美沙拉嗪联合丹参注射液的应用[J].中国社区医师,2020,36(20):76-77.
697. 朱凤池,杨静,刘超,乔茶,马靖,苏静伟.参苓白术丸联合美沙拉嗪治疗溃疡性结肠炎的疗效观察[J].中国中西医结合外科杂志,2020,26(04):715-718.
698. 朱超,常凤玲.白术黄芪汤对溃疡性结肠炎患者证候积分及炎性反应的影响[J].现代诊断与治疗,2020,31(08):1190-1191.
699. 李克亚,王真权,彭美瑶.乌梅丸治疗溃疡性结肠炎（脾肾虚寒，寒中蕴热证）的疗效及对肠道微生态的影响[J].中医药导报,2020,26(16):85-89.
700. 李剑,赵红波.自拟补脾益肠汤辅助治疗溃疡性结肠炎临床研究[J].四川中医,2020,38(04):114-117.
701. 李吉庆,朱静.清溃愈疡汤保留灌肠联合美沙拉嗪栓治疗直肠型溃疡性结肠炎临床观察[J].实用中医药杂志,2020,36(05):607-608.
702. 李开望.自拟中药清肠愈疡汤结合西药治疗溃疡性结肠炎疗效观察[J].中国处方药,2020,18(04):132-133.
703. 李玉,赵琳,李上达,李培.翁榆合剂联合中医特色护理对慢性非特异性溃疡性结肠炎(湿热内蕴证)的临床疗效观察[J].四川中医,2020,38(05):211-213.
704. 李芳,奚美娟,张平,徐璐,郭天威.祛湿愈疡方保留灌肠联合美沙拉嗪治疗溃疡性结肠炎大肠湿热证疗效观察[J].实用药物与临床,2020,23(07):599-602.
705. 杜学芳,马尚超,张彩凤,张超群,李光艳,张兰芳,张利利,郭晓鹤,顾莉莉,杨妮,司玉静.芪倍合剂联合美沙拉嗪对活动期溃疡性结肠炎患者Th17、Treg细胞及其相关细胞因子水平的影响[J].药物评价研究,2020,43(11):2254-2258.
706. 杨娜.西药联合热瘀散、中药灌肠治疗溃疡性结肠炎43例临床观察[J].中国民族民间医药,2020,29(22):110-112.
707. 杨娜.益气解毒化瘀方联合美沙拉嗪肠溶片治疗慢性复发型溃疡性结肠炎效果观察[J].实用中医药杂志,2020,36(08):1023-1024.
708. 杨影,房栩丞,郑萍.自拟结肠宁汤保留灌肠治疗慢性溃疡性结肠炎的临床观察[J].新疆中医药,2020,38(06):13-15.
709. 杨慧珍.锡类散灌肠联合柳氮磺吡啶栓治疗溃疡性结肠炎的临床观察[J].中国民间疗法,2020,28(18):70-72.
710. 林美容.穴位贴敷配合耳穴治疗对溃疡性结肠炎患者营养状况的影响[J].内蒙古中医药,2020,39(07):130-131.
711. 梁想,王梅青.参苓白术散联合美沙拉嗪治疗溃疡性结肠炎疗效研究[J].陕西中医,2020,41(09):1251-1253.
712. 梁霞,李锦,伍文. 中西医结合治疗溃疡性结肠炎的临床观察[J]. 中国处方药,2020,18(8):144-146.
713. 樊静娜,赵继亭,闫华,孙大娟,王帅,梁峻尉,尹德菲,苗秀明,迟莉丽.安肠愈疡汤联合美沙拉秦治疗脾虚湿阻型溃疡性结肠炎的疗效评价及对炎性因子的影响[J].中华中医药杂志,2020,35(07):3765-3770.
714. 毕文秋.白头翁汤结合针灸治疗溃疡性结肠炎对患者免疫功能及IL-1β、IL-17的影响[J].现代医学与健康研究电子杂志,2020,4(10):96-98.
715. 王凯,马丙旭,刘利萍.解毒化浊促愈汤联合美沙拉嗪肠溶片治疗溃疡性结肠炎的疗效及对炎性细胞因子的影响[J].中医临床研究,2020,12(03):77-78+86.
716. 王昆,韦梅.芍药汤加减结合针刺治疗溃疡性结肠炎湿热蕴结证活动期疗效观察[J].现代中西医结合杂志,2020,29(07):763-766.
717. 王晓明.美沙拉嗪联合丹参注射液治疗溃疡性结肠炎的效果探究[J].中国实用医药,2020,15(03):135-137.
718. 王海萍.清热化湿饮联合针刺治疗溃疡性结肠炎临床疗效及对血清炎症因子的影响[J].湖北中医药大学学报,2020,22(02):78-80.
719. 王红娟.自拟清热燥湿化瘀汤口服灌肠治疗溃疡性结肠炎大肠湿热证临床研究[J].四川中医,2020,38(11):109-113.
720. 王金周,王萍,侯亭开,张运希.固肠止泻丸联合康复新液灌肠治疗对溃疡性结肠炎患者肠道菌群及炎症因子的影响[J].世界中西医结合杂志,2020,15(12):2289-2293.
721. 王飞,毕信亚,周莉,钱华松.温阳止泻汤联合温针灸治疗脾肾阳虚型溃疡性结肠炎疗效观察[J].现代中西医结合杂志,2020,29(31):3496-3499.
722. 王腾飞,黄一薇.中医综合疗法联合美沙拉嗪治疗溃疡性结肠炎的临床观察[J].智慧健康,2020,6(19):138-139+164.
723. 白宁,刘巍,张光宇.清肠愈疡汤联合美沙拉嗪治疗溃疡性结肠炎临床观察[J].中国中医药现代远程教育,2020,18(12):123-125.
724. 白鹏飞,张艳君,李敏.荆芥连翘汤加减从肺论治溃疡性结肠炎疗效及对患者肠黏膜屏障功能、免疫调节、氧化应激指标的影响[J].陕西中医,2020,41(07):897-900.
725. 石超学.中药保留灌肠联合穴位按摩干预活动期溃疡性结肠炎效果研究[J].新中医,2020,52(11):161-163.
726. 窦鹏鹏.美沙拉嗪配合中药口服治疗溃疡性结肠炎35例疗效观察[J].中国肛肠病杂志,2020,40(01):34-35.
727. 翟永治.中西医结合治疗溃疡性结肠炎疗效观察[J].实用中医药杂志,2020,36(04):478-479.
728. 聂珍静,申文静,姚蕊,王欣蕾.白头翁汤口服联合美沙拉嗪灌肠治疗溃疡性结肠炎的效果[J].中国医药导报,2020,17(33):149-152+169.
729. 聂荣慧.自拟中药清肠愈疡汤对溃疡性结肠炎患者肠道菌群及炎性状态的影响[J].现代医学与健康研究电子杂志,2020,4(24):65-67.
730. 肖小龙. 三仁汤加减联合美沙拉嗪治疗湿热型溃疡性结肠炎的临床观察[D].成都中医药大学,2020.
731. 苏广春.地塞米松联合云南白药保留灌肠在溃疡性结肠炎治疗中的应用疗效分析[J].中国肛肠病杂志,2020,40(05):53-54.
732. 蒋华,武国营,蒋荣伟.柴胡疏肝散联合美沙拉秦肠溶片治疗溃疡性结肠炎的疗效及对炎症因子及血流动力学指标的影响[J].血栓与止血学,2020,26(05):745-747.
733. 许玲,盛好,陈健.改良灌肠袋直肠滴入肠炎灵1号方治疗激素抵抗型重症溃疡性结肠炎疗效及对肠道菌群和炎症因子的影响[J].现代中西医结合杂志,2020,29(04):367-370+375.
734. 谭高展,孙俊,屈银宗,周芳,裴畅,冯涛.芍药汤联合英夫利昔单抗治疗中重度溃疡性结肠炎临床观察[J].山西中医,2020,36(07):23-26.
735. 赵克学,徐辰,戴高中,张征波,沈旦蕾,范先靖,袁蒲.愈疡膏外敷联合西药治疗复发型活动期溃疡性结肠炎临床研究[J].陕西中医,2020,41(05):600-604.
736. 赵岩,武虎.美沙拉嗪联合温针灸治疗溃疡性结肠炎疗效及对肠黏膜组织炎症因子及Th17、Th22亚群的影响[J].现代中西医结合杂志,2020,29(01):55-59.
737. 赵胜乾.建中化湿汤联合柳氮磺胺吡啶片治疗溃疡性结肠炎临床观察[J].实用中医药杂志,2020,36(06):755-756.
738. 邓旦. 参苓白术散加减联合美沙拉嗪治疗溃疡性结肠炎的临床观察[J]. 世界最新医学信息文摘（连续型电子期刊）,2020,20(11):170,172.
739. 郭师. 康复新液联合美沙拉嗪治疗溃疡性结肠炎临床观察[J]. 实用中医药杂志,2020,36(11):1448-1449.
740. 郭志伟,徐跃军,唐迎春,车浩,陈艳,黄婷,孙易娜.肠炎1号对肝郁脾虚型溃疡性结肠炎患者的疗效[J].世界中医药,2020,15(13):1972-1976.
741. 钟彩玲,郭淳,王师英,赵喜颖,张北平.调肠消炎片对轻中度溃疡性结肠炎患者血清细胞因子的影响[J].广州中医药大学学报,2020,37(02):226-233.
742. 闫丽华,帅飞.化浊清解方联合柳氮磺吡啶对溃疡性结肠炎患者肠黏膜屏障功能、血清炎症因子及其机制研究[J].药物生物技术,2020,27(04):319-323.
743. 闫文慧,闫文峰.调补脾胃中药治疗脾胃虚弱型溃疡性结肠炎的疗效观察[J].中国疗养医学,2020,29(03):335-336.
744. 陈天杰,张明红.参苓白术散治疗慢性复发型溃疡性结肠炎的疗效及基于β_2AR/β-arrestin2/NF-κB信号转导通路的作用机制[J].中药材,2020,43(04):996-999.
745. 陈柏谦,李瑛.大柴胡汤联合柳氮磺胺吡啶肠溶片治疗溃疡性结肠炎的效果观察[J].中国医学创新,2020,17(15):66-69.
746. 陈立平,王国海,周志红,谭仁琼.大柴胡汤治疗溃疡性结肠炎的临床研究[J].现代中医药,2020,40(03):88-91.
747. 雷娜,孔鹏飞,陈思敏,唐学贵.槐花散合桃花汤加减对溃疡性结肠炎活动期寒热错杂证免疫炎症的调节作用[J].中国实验方剂学杂志,2020,26(07):86-91.
748. 雷彪,冯文哲,石鹏,牛魁,柴小琴.白头翁汤加减治疗轻中度溃疡性结肠炎急性发作临床观察[J].中国临床研究,2020,33(12):1681-1683+1687.
749. 韩晓丽,席作武,王凯.清热利湿方联合美沙拉嗪治疗溃疡性结肠炎38例疗效观察[J].国医论坛,2020,35(01):47-49.
750. 顾涛.中西医结合治疗溃疡性结肠炎临床观察[J].实用中医药杂志,2020,36(09):1158-1159.
751. 马太成,王爱红,高寰,等. 中西医结合治疗溃疡性结肠炎的疗效观察及作用机制探讨[J]. 养生保健指南,2020(34):260.
752. 马月香,侯凤霞,李洪海.白头翁汤加减联合西药治疗大肠湿热型溃疡性结肠炎疗效观察[J].辽宁中医杂志,2020,47(01):114-116.
753. 高强,彭文梅. 中西医结合治疗溃疡性结肠炎的优势及临床应用[J]. 中国保健营养,2020,30(13):54-55.
754. 魏永辉,单海燕,戚丹凤,刘钰,陈伟,李春耕,李松柱,赵宇琦,朱叶珊,张茹.清热化湿调气通络法治疗急性溃疡性结肠炎的临床研究[J].中国中医急症,2020,29(05):817-819.
755. 黄素素.中西药联合治疗溃疡性结肠直肠炎的疗效及安全性分析[J].临床合理用药杂志,2020,13(07):66-67.
756. 黄银僖,高建辉,马鑫文,何俊峰,刘俊昌.脏腑推拿联合美沙拉嗪治疗发作期溃疡性结肠炎的临床观察[J].新疆中医药,2020,38(03):22-24.
757. 齐淑文,汪益兴.中药灌肠对湿热型急性溃疡性结肠炎的疗效及效果评价[J].现代消化及介入诊疗,2020(S01):0350-0351.
758. Li S, Hao X, Gong Y, Liu S, Niu W, Jia J, Tang Y. Effect of shenling baizhu powder on the serum TH1 cytokines of elderly patients with ulcerative colitis complicated by bloody purulent stool. Am J Transl Res. 2021 Aug 15;13(8):9701-9707. PMID: 34540098; PMCID: PMC8430119.
759. Shen H, Zhang S, Zhao W, Ren S, Ke X, Gu Q, Tang Z, Xie J, Chen S, Chen Y, Zou J, Zhang L, Shen Z, Zheng K, Liu Y, Gu P, Cheng J, Hu J, Zhu L. Randomised clinical trial: Efficacy and safety of Qing-Chang-Hua-Shi granules in a multicenter, randomized, and double-blind clinical trial of patients with moderately active ulcerative colitis. Biomed Pharmacother. 2021 Jul;139:111580. doi: 10.1016/j.biopha.2021.111580. Epub 2021 Apr 13. PMID: 33857914.
760. 丁益宏,田巍巍,韩晓梅,陈恳.愈疡合剂直肠滴注治疗大肠湿热型溃疡性结肠炎的临床研究[J].南通大学学报(医学版),2021,41(06):569-571.
761. 丛龙玲,姚嘉茵,吴宇金,吕永慧.肠炎清合剂联合美沙拉嗪肠溶片维持治疗缓解期溃疡性结肠炎的临床疗效[J].中国实验方剂学杂志,2021,27(03):99-104.
762. 尹平,李炜,杨红梅,董曼,宋君宇.加味芍药汤和康复新液保留灌肠联合美沙拉嗪肠溶片口服治疗大肠湿热型溃疡性结肠炎的临床研究[J].河北中医,2021,43(02):278-282.
763. 于策.翁连解毒汤结合美沙拉嗪肠溶片治疗溃疡性结肠炎的临床效果分析[J].中国实用医药,2021,16(31):150-152.
764. 何建伟,丁益宏,韩晓梅,田巍巍,夏小丽.愈疡合剂直肠滴注疗法对溃疡性结肠炎大肠湿热型患者中医证候、血清IL-6、IL-10、TNF-α水平的影响[J].四川中医,2021,39(10):98-101.
765. 刘凌华.白头翁汤加减+美沙拉嗪治疗湿热型溃疡性结肠炎的临床效果[J].中国现代药物应用,2021,15(18):203-205.
766. 刘宝通,刘位. 中医综合疗法联合美沙拉嗪治疗溃疡性结肠炎的临床观察[J]. 健康必读,2021(11):158,161.
767. 刘明顺. 补脾泻火方联合美沙拉嗪治疗溃疡性结肠炎临床观察[J]. 实用中医药杂志,2021,37(3):458-460.
768. 刘荣明,颜玲玲. 健脾清热利湿愈疡汤联合柳氮磺吡啶治疗溃疡性结肠炎的临床观察[J]. 中国中医药科技,2021,28(4):682-684.
769. 刘超. 中西医结合治疗溃疡性结肠炎患者疗效及对血小板、凝血功能及炎性因子的影响[J]. 血栓与止血学,2021,27(2):229-230.
770. 刘二委. 中西医结合治疗中度溃疡性结肠炎67例临床观察[J]. 中国民族民间医药,2021,30(1):113-115.
771. 卜婷婷. 结肠宁灌肠调护联合美沙拉嗪治疗溃疡性结肠炎患者的临床效果[J]. 中国医药科学,2021,11(13):76-79.
772. 周丽,曾玲玲,季小健.温针灸联合参苓白术散治疗溃疡性结肠炎脾虚湿阻证的疗效及对脑-肠互动和炎症因子的影响[J].河北中医,2021,43(09):1483-1487+1524.
773. 周军怀.三皮愈疡汤灌肠联合小剂量美沙拉嗪口服治疗溃疡性结肠炎45例[J].中医学,2021,10(5):678-681.
774. 周屹,彭勇,贺平,肖小龙.三仁汤加减口服联合经验方灌肠治疗湿热型溃疡性结肠炎临床观察[J].四川中医,2021,39(04):114-118.
775. 周铖,王阳阳,陈金鑫,刘芳芳.白头翁汤加减保留灌肠治疗溃疡性结肠炎临床观察[J].辽宁中医杂志,2021,48(09):121-124.
776. 喻婷,胡德胜,楚思,刘星星,范恒.复方苦参汤联合美沙拉嗪对溃疡性结肠炎治疗效果及炎性因子水平的影响[J].现代生物医学进展,2021,21(01):50-53+91.
777. 国龙溪,吴华清,张广林,刘秋芹.愈疡灌肠方联合西药治疗溃疡性结肠炎湿热内蕴症的临床研究[J].系统医学,2021,6(18):74-76.
778. 夏敏,王红,张曼麒.中西医结合治疗溃疡性结肠炎的疗效观察[J].基层医学论坛,2021,25(36):5250-5252.
779. 宋晓微.葛根芩连汤结合聚焦解决护理模式对急性溃疡性结肠炎患者免疫功能及炎症反应的影响[J].光明中医,2021,36(15):2625-2628.
780. 席岚岚,宋太平,史孟华,刘全林,宋光瑞.补阳止泻汤加减治疗脾肾阳虚型溃疡性结肠炎的临床研究[J].中医药导报,2021,27(07):86-89+92.
781. 康雪.黄连清胃汤对溃疡性结肠炎患者肠道菌群及氧化应激的影响[J].中国中医药现代远程教育,2021,19(23):104-105+123.
782. 张冬梅,张雅明,夏泽华.清肠愈疡汤口服及中药灌肠对溃疡性结肠炎患者血清I-FABP、sRAGE和p-ANCA水平的影响[J].西部中医药,2021,34(03):14-18.
783. 张学峰.乌梅汤对溃疡性结肠炎患者Hgb、ESR、CRP及免疫功能的影响[J].内蒙古中医药,2021,40(08):66-67.
784. 张广林,国龙溪,刘秋芹,吴华清.中西医结合治疗溃疡性结肠炎患者的应用效果探究[J].系统医学,2021,6(14):69-71.
785. 张新,卢冬雪. 香连丸加减治疗溃疡性结肠炎的疗效观察[J]. 当代医药论丛,2021,19(16):169-170.
786. 彭云花,唐诚,陈天,王清园,杨巍.实证肠炎方联合美沙拉嗪治疗中重度大肠湿热型溃疡性结肠炎临床观察[J].浙江中医药大学学报,2021,45(03):260-264+269.
787. 徐敏嫦,苏振坤,林维茂,陈转艮,陈婷,陈润添.中西药合用治疗活动期溃疡性结肠炎大肠湿热型临床观察[J].实用中医药杂志,2021,37(01):60-61.
788. 惠慧,谷云飞,缪志伟.清热利湿方联合美沙拉嗪治疗溃疡性结肠炎的临床效果[J].中国中西医结合外科杂志,2021,27(02):272-277.
789. 房莹莹,耿宏亮,张金鑫. 分析美沙拉嗪联合中药汤剂治疗溃疡性结肠炎的临床效果[J]. 中国保健营养,2021,31(21):266.
790. 敖雪仁,廖聪,马凯敏,沈国喜.祛湿促愈汤结合灌肠疗法对轻中度溃疡性结肠炎急性期患者结肠黏膜愈合的促进作用[J].四川中医,2021,39(08):83-86.
791. 曲卓.涤肠愈疡灌肠方联合西药治疗溃疡性结肠炎临床观察[J].中国中医药现代远程教育,2021,19(15):119-121.
792. 朱伟宁,王丽媛,姜坤.自拟扶正祛邪愈溃汤治疗寒热错杂型溃疡性结肠炎的临床效果[J].中国医药导报,2021,18(27):142-145.
793. 朱燕,刘全喜,王韶华,刘大铭,陈亮.芍药汤加减联合美沙拉嗪治疗大肠湿热型溃疡性结肠炎35例临床观察[J].世界中西医结合杂志,2021,16(09):1653-1657.
794. 朱磊,沈洪,张声生,赵文霞,任顺平,柯晓,顾庆华,唐志鹏,谢晶日,陈苏宁,陈延,邹建东,沈照峰,张露,刘亚军.清热祛湿、凉血化瘀法治疗中度活动期溃疡性结肠炎大肠湿热证多中心、随机对照、双盲的临床研究[J].中国中西医结合消化杂志,2021,29(10):681-685+690.
795. 李丽梅,王永强.参苓白术散联合美沙拉嗪治疗溃疡性结肠炎临床观察[J].西部中医药,2021,34(10):124-126.
796. 李亚,刘岩,秦燕鸿,卢军仪.健脾益气、清热化腐生肌方联合美沙拉嗪对溃疡性结肠炎患者肠黏膜屏障功能的影响[J].中国中西医结合消化杂志,2021,29(07):455-459+463.
797. 李亚,王婧,张全乐,卢军仪.中药组方保留灌肠治疗轻中度溃疡性结肠炎的临床疗效及安全性观察[J].世界中西医结合杂志,2021,16(11):2100-2104.
798. 李向阳,唐泉淼,聂萌,王雷.资生丸联合柳氮磺吡啶肠溶片治疗溃疡性结肠炎的临床研究[J].现代药物与临床,2021,36(03):502-506.
799. 李振伟,牛栋良,赵松伟.中西医结合治疗溃疡性结肠炎43例临床观察[J].中国民族民间医药,2021,30(15):84-86+103.
800. 李敏,刘肖.甘草泻心汤联合龙血竭胶囊治疗溃疡性结肠炎临床研究[J].河南中医,2021,41(06):840-843.
801. 李春明,曹玉杰,师恩惠,徐晓霞.中西医联合治疗炎症性肠病患者的效果[J].中国民康医学,2021,33(03):97-99.
802. 李春燕,郭玮,韩帅.中药保留灌肠联合口服美沙拉嗪治疗溃疡性结肠炎临床观察[J].内蒙古中医药,2021,40(04):104-106.
803. 李泽涵,尚洪海,赵彩琪,靳桂春,冯五金.青黛散加减联合西药对急性期溃疡性结肠炎患者炎症-氧化应激的干预研究[J].中国中医急症,2021,30(05):801-804+808.
804. 李红薇.温针灸联合美沙拉嗪对溃疡性结肠炎患者免疫功能及TNF-α、IL-1β、IL-10水平的影响[J].中医外治杂志,2021,30(06):16-17.
805. 李达,车志英.收敛除溃汤联合美沙拉嗪治疗溃疡性结肠炎对T淋巴细胞亚群及炎症指标的影响[J].实用中医药杂志,2021,37(07):1210-1212.
806. 杨娅娟,王凯,席作武,高宗跃.结肠清热丸联合西医常规疗法治疗溃疡性结肠炎活动期大肠湿热证的临床观察[J].中国实验方剂学杂志,2021,27(09):112-117.
807. 杨柳,王秀娟,金铭,李妍.加味葛根芩连汤对轻中度湿热蕴结型溃疡性结肠炎的疗效及部分机制研究[J].中国处方药,2021,19(10):140-142.
808. 杨颖,王昊,黄清,娄华,臧宾宾.健脾补督汤联合美沙拉嗪对缓解期溃疡性结肠炎患者的临床疗效[J].中成药,2021,43(11):3023-3027.
809. 沈灵娜,刘军,钱赟达,熊烈.四神丸合参苓白术散联合美沙拉嗪治疗溃疡性结肠炎临床疗效及对肠道菌群、肠黏膜屏障功能的影响[J].新中医,2021,53(16):34-38.
810. 沈灵娜,刘军,钱赟达,熊烈.甘草泻心汤联合美沙拉嗪对溃疡性结肠炎患者疗效及肠道菌群和血清炎症因子水平的影响[J].中国中西医结合消化杂志,2021,29(07):474-478.
811. 渠磊.中西医结合治疗溃疡性结肠炎疗效观察[J].实用中医药杂志,2021,37(04):605-606.
812. 王亿鹏,刘华一.自拟扶正祛邪方联合西药治疗溃疡性结肠炎的临床疗效及安全性分析[J].四川中医,2021,39(11):119-123.
813. 王俊敏.温肾健脾理肠汤联合美沙拉嗪治疗脾肾阳虚型溃疡性结肠炎的临床观察[J].中国中医药科技,2021,28(05):821-822.
814. 王向军,邱雪霏.中药保留灌肠治疗激素依赖型溃疡性结肠炎临床观察[J].湖北中医药大学学报,2021,23(03):75-77.
815. 王晓红,胡兵,铉力.美沙拉嗪联合结肠炎Ⅰ号治疗31例溃疡性结肠炎患者的临床疗效及对炎症因子的影响[J].世界中西医结合杂志,2021,16(08):1476-1480.
816. 王柯棣,李晨.芍黄安肠汤联合美沙拉嗪肠溶片治疗活动期溃疡性结肠炎大肠湿热证临床观察[J].实用中医药杂志,2021,37(05):785-786.
817. 王森.参苓白术丸联合美沙拉嗪治疗溃疡性结肠炎患者的临床效果[J].中国药物经济学,2021,16(10):55-57+65.
818. 王燕玲,刘蔚.丹参注射液联合复合乳酸菌胶囊治疗溃疡性结肠炎的疗效观察[J].临床医药实践,2021,30(04):280-281+320.
819. 申军华,靳文军.中药灌肠联合健脾清肠方加减及美沙拉嗪治疗溃疡性结肠炎患者氧化应激反应的临床观察[J].世界中西医结合杂志,2021,16(09):1658-1662+1666.
820. 申文静,姚蕊,聂珍静.改良白头翁汤结合麦滋林-S灌肠调节溃疡性结肠炎患者机体炎症反应及免疫功能的作用研究[J].中国中西医结合消化杂志,2021,29(10):702-706.
821. 相兵.丹参注射液结合柳氮磺吡啶治疗溃疡性结肠炎患者的临床观察及对COX-2、NF-κB水平的影响[J].中国处方药,2021,19(07):126-128.
822. 石鹏岩.自拟中药方加减联合美沙拉嗪肠溶片治疗溃疡性结肠炎缓解期20例临床观察[J].中国民间疗法,2021,29(15):74-77.
823. 娄华,乔虹,王昊.健脾补督汤、循督火龙灸联合美沙拉嗪对缓解期溃疡性结肠炎患者的临床疗效[J].中成药,2021,43(10):2919-2922.
824. 翟进诚,闵家星.中药保留灌肠治疗溃疡性结肠炎32例疗效观察[J].中国肛肠病杂志,2021,41(10):28-30.
825. 聂光荣.中西医结合治疗溃疡性结肠炎临床疗效对照研究[J].中医临床研究,2021,13(09):72-73+76.
826. 胡婕,郭修田,周大成,姜东萍,金文琪,李小嘉,杨巍.黄芩汤加减结合美沙拉嗪对溃疡性结肠炎(活动期)肠黏膜组织炎症因子的影响[J].中华中医药学刊,2021,39(01):123-126.
827. 胡建成.中西医结合疗法治疗溃疡性结肠炎临床观察[J].中国社区医师,2021,37(19):92-93.
828. 葛飞,刘丽娜,严晶,康安,朱时林,田祖成,梅莉,代海峰,倪海军.金荞麦联合SASP治疗大肠湿热型UC的疗效及其对TLR4/NLRP3信号通路的影响[J].南京中医药大学学报,2021,37(01):16-20.
829. 薛会才,徐艳.加味芍药汤治疗溃疡性结肠炎的临床疗效[J].内蒙古中医药,2021,40(01):35-36.
830. 谭首海,于秀芝,刘海龙,王文航.益气解毒方联合美沙拉嗪肠溶片治疗溃疡性结肠炎临床研究[J].新中医,2021,53(04):91-94.
831. 许子臣,王绕绕,谷鹏,李玥.金胃泰胶囊联合柳氮磺吡啶治疗慢性溃疡性结肠炎的临床研究[J].现代药物与临床,2021,36(09):1876-1879.
832. 许宝才,陈伟,邱根祥. 雷氏隔姜脐灸疗法治疗溃疡性结肠炎脾虚湿蕴证的效果观察[J]. 浙江临床医学,2021,23(12):1749-1752.
833. 许莉嘉. 荆芥连翘汤加减治疗溃疡性结肠炎的疗效观察[J]. 中国现代药物应用,2021,15(8):205-207.
834. 谷丽瑶,罗敏.葛根芩连汤加减联合美沙拉嗪治疗湿热型溃疡性结肠炎临床观察[J].山西中医,2021,37(11):25-27.
835. 赵明,王德芳,苏晓兰.清热健脾方联合美沙拉嗪治疗溃疡性结肠炎（大肠湿热证）的临床研究[J].中医药导报,2021,27(04):96-99+111.
836. 郁峰,沈锋,黄路桥.参白汤联合美沙拉嗪对大肠湿热型溃疡性结肠炎的疗效及其对IL-27表达的影响[J].浙江医学,2021,43(06):593-596+692.
837. 郭富彬,包海兰,徐伟.祛邪通络汤联合美沙拉嗪治疗溃疡性结肠炎临床观察[J].中国中医药现代远程教育,2021,19(08):139-141.
838. 鄂辉,杨娇,杨泽江,严晓红,刘雅丽.连理汤联合美沙拉嗪对溃疡性结肠炎患者临床疗效的影响分析[J].中国中西医结合消化杂志,2021,29(11):778-782.
839. 钱雪芬,孟云霞,徐大洲.基于糖萼损伤研究金银花水煎液治疗溃疡性结肠炎的作用机制[J].现代中西医结合杂志,2021,30(10):1079-1082+1140.
840. 阙茂棋,洪德群,袁秀芳.柴胡桂枝干姜汤联合美沙拉嗪治疗寒热错杂型溃疡性结肠炎的效果观察[J].中外医学研究,2021,19(24):105-107.
841. 陈光亮.香砂六君子汤加味联合美沙拉秦治疗溃疡性结肠炎[J].实用中西医结合临床,2021,21(11):12-13+73.
842. 陈斓,张彧青,李慕然,苏啸天.健脾止泻宁颗粒联合美沙拉嗪治疗溃疡性结肠炎的临床研究[J].现代药物与临床,2021,36(05):947-951.
843. 陈燕,章小艳,黄益倩,童晓清,郑培奋,汪涛,束龙.葛根芩连汤加味联合低FODMAP饮食对溃疡性结肠炎患者的疗效影响[J].医学研究杂志,2021,50(02):69-73.
844. 韩克舜.痛泻要方加减在溃疡性结肠炎治疗中的应用效果[J].河南医学研究,2021,30(16):3006-3008.
845. 韩柯.清热利湿益肠汤联合针灸治疗溃疡性结肠炎的效果及对血清肿瘤坏死因子α白细胞介素8水平的影响[J].实用医技杂志,2021,28(02):215-216.
846. 马俊洲,毛细云.中药灌肠联合美沙拉嗪栓塞肛治疗溃疡性结肠炎疗效观察[J].江西中医药大学学报,2021,33(06):46-48.
847. 黄胜伟,辛学知,马学芹,杜金鑫,郭梦雯,庞雪.从肝论治溃疡性结肠炎38例的疗效观察[J].中医临床研究,2021,13(21):59-61.
848. 王垚,王芳,王雪.解毒固本汤联合西药治疗溃疡性结肠炎的疗效及对肠道菌群、免疫功能的影响[J].新中医,2022,54(03):80-84.
849. 莫达瑜,刘英超,王飞达,倪思忆,沈雁.乌灵胶囊联合氟哌噻吨美利曲辛片对活动期溃疡性结肠炎伴焦虑和抑郁患者的影响研究[J].中国全科医学,2022,25(03):285-292.
850. 郑军营,刘春强,张科艺.真武汤加味联合美沙拉嗪治疗溃疡性结肠炎的临床疗效研究[J].中国中医急症,2022,31(01):35-37.
851. 金艳,李向.红藤败酱白头翁汤联合美沙拉嗪缓释颗粒剂治疗大肠湿热型溃疡性结肠炎[J].中医学报,2018,33(11):2226-2229.
852. 周翔英,徐欣欣,吴永华,俞树瀚,史莹,陈永灿.健脾化浊汤联合美沙拉嗪治疗溃疡性结肠炎42例临床观察[J].浙江中医杂志,2021,56(01):40-41.
853. 余媛媛. 中西结合治疗溃疡性结肠炎的临床研究[D].湖北中医学院,2008.
854. 党晓眠,王强.中西医结合治疗慢性溃疡性结肠炎50例[J].现代中医药,2008(04):35-36.
855. 刘庆善.中西医结合辨证分型治疗慢性非特异性溃疡性结肠炎分析[J].现代中西医结合杂志,2008(02):192-193.
856. 刘文英,孟惠改,薛芳.中药灌肠合复方谷氨酰胺治疗慢性非特异性溃疡性结肠炎的临床研究[J].河北中医,2008,30(11):1182-1183.
857. 刘育才,吴建明.中西医结合治疗溃疡性结肠炎46例观察[J].实用中医药杂志,2008(04):232-233.
858. 吕音勇.中西医结合治疗慢性溃疡性结肠炎临床观察[J].中国现代药物应用,2008(10):64-65.
859. 吴越平.复方灌肠液联合健脾化湿汤治疗溃疡性结肠炎60例[J].临床医学,2008(11):115-116.
860. 吴春华,李彬,董子亮.溃结方联合柳氮磺胺吡啶治疗溃疡性结肠炎临床观察[J].中国中医药信息杂志,2008(04):69-70.
861. 周淑风,刘建国.中西医结合治疗重症溃疡性结肠炎54例[J].陕西中医,2008(01):52-53.
862. 孔小云.肠炎清片配合柳氮磺胺吡啶治疗慢性非特异性溃疡性结肠炎疗效观察[J].中国中医急症,2008(08):1065+1073.
863. 孙真理,龙发伟. 中西药交替灌肠治疗溃疡性结肠炎30例[J]. 国际外科学杂志,2008,35(3):164-166.
864. 孟惠改,刘文英,白晓莉,薛芳.肠炎Ⅰ配合Ⅱ灌肠配合复方谷氨酰胺治疗UC缓解期的临床报道[J].光明中医,2008(09):1291-1293.
865. 常东,黄志新,樊亚巍,陶永胜,李辉,朱圣鹰,张东坡.溃结灵颗粒对大肠湿热型溃疡性结肠炎患者Toll-like受体等指标的影响[J].中国中医药信息杂志,2008(01):11-13.
866. 张明.中西医结合治疗慢性溃疡性结肠炎疗效观察[J].青海医药杂志,2008(07):82-83.
867. 张金卓,张彦,郝英霞,齐红兵.丹参注射液对溃疡性结肠炎患者D-二聚体含量的影响及意义[J].现代中西医结合杂志,2008(32):4968-4969.
868. 徐吉星,郭志峰.中西医结合治疗溃疡性结肠炎48例[J].中国医药指南,2008,6(23):168.
869. 徐新华.中西医结合治疗溃疡性结肠炎疗效观察[J].实用中医药杂志,2008(08):511.
870. 徐永辉,邹昀,李太荣. 中西医结合治疗溃疡性结肠炎63例临床疗效观察[J]. 中国临床实用医学,2008,2(3):102-103.
871. 曹克勇,韦丽.柳氮磺吡啶联合白头翁汤灌肠治疗溃疡性结肠炎32例[J].中国中西医结合消化杂志,2008,16(06):407-408.
872. 李俊才.美沙拉嗪口服联合中药灌肠治疗儿童溃疡性结肠炎的疗效分析[J].中国误诊学杂志,2008(15):3557-3558.
873. 李晓兰,李兴谦,陈凯,马庆峰,杨清.中西药物保留灌肠合并针灸治疗溃疡性结肠炎的临床研究[J].结直肠肛门外科,2008(03):161-163.
874. 李海强,周翼,张冬琼,罗昭芳.艾箱灸神阙、上下巨虚穴治疗慢性溃疡性结肠炎[J].针灸临床杂志,2008(09):33-35.
875. 李红庆.思密达和锡类散中西医结合治疗溃疡性结肠炎25例临床观察[J].临床医药实践杂志,2008,17(S5):1018-1019.
876. 杨朝霞,李仙瑛.中西药结合灌肠治疗溃疡性结肠炎的疗效对比观察[J].实用中西医结合临床,2008,6(06):16-17.
877. 温建云.中西医结合治疗慢性溃疡性结肠炎27例疗效观察[J].中国现代药物应用,2008(13):61-62.
878. 王新,张跃.中西医结合治疗溃疡性结肠炎31例临床观察[J].云南中医中药杂志,2008(05):25.
879. 王烨,赵喜文. 中西医结合治疗慢性溃疡性结肠炎28例临床观察[J]. 中国肛肠病杂志,2008,28(6):51-52.
880. 贺静,王海燕. 中西医结合保留灌肠治疗溃疡性结肠炎的疗效观察[J]. 临床心身疾病杂志,2016,22(z2):66-67.
881. 田自力,郭淑静,朱淑芳,等. 穴位埋线联合柳氮磺胺吡啶治疗溃疡性结肠炎的疗效及对抗中性粒细胞浆抗体的影响[J]. 中国综合临床,2008,24(10):1029-1031.
882. 祝水国,吴陆香. 溃结方配合穴位注射治疗溃疡性结肠炎65例[J]. 中国肛肠病杂志,2008,28(4):37-39.
883. 肖景东,白光.中西医结合治疗溃疡性结肠炎临床观察[J].实用中医药杂志,2008(06):369.
884. 胡咏华,廖文峰.中西药联用保留灌肠治疗溃疡性结肠炎43例小结[J].中医药导报,2008(06):34+38.
885. 苏东平,王冬.中西医结合灌肠治疗溃疡性结肠炎临床观察[J].山西中医,2008(06):34.
886. 蔡懋戈,蔡丹青.三种灌肠方法治疗溃疡性结肠炎的疗效比较[J].现代中医药,2008(02):8-9.
887. 许贤姬,张可,王宇.生脉注射液辅助治疗溃疡性结肠炎50例[J].中国中西医结合消化杂志,2008(05):343-344.
888. 赖光强.中西医结合治疗慢性溃疡性结肠炎疗效观察[J].深圳中西医结合杂志,2008(02):107-108.
889. 赵厚良.复方丹参对溃疡性结肠炎治疗效果的影响[J].社区医学杂志,2008(13):38.
890. 邹昀,李太荣.中西医结合治疗溃疡性结肠炎临床疗效观察[J].实用药物与临床,2008(04):206-207.
891. 邹晓华,石美雅,查安生,章小平,张树卿.清热解毒法治疗溃疡性结肠炎的临床观察[J].光明中医,2008(04):469.
892. 郭锋.中西医结合治疗溃疡性结肠炎66例[J].光明中医,2008(11):1754-1755.
893. 金男日,张丹.中西医结合治疗溃疡性结肠炎疗效观察[J].牡丹江医学院学报,2008(02):47-48.
894. 陆宇平,王长洪,王立新,杨卓,麻树人,陈山泉,刘杨,高文艳,巩阳,林一帆,朱虹.中西医结合治疗溃疡性结肠炎的临床研究[J].中国中西医结合消化杂志,2008(02):91-93.
895. 陈凯,李晓兰,杨清,李兴谦.保留灌肠联合针灸治疗溃疡性结肠炎疗效观察[J].中国煤炭工业医学杂志,2008(06):819-820.
896. 陈爱武,宋刘来.穴位埋线法加SASP治疗慢性溃疡性结肠炎[J].中国康复,2008(05):348.
897. 陈红.口服给药联合中药保留灌肠治疗溃疡性结肠炎的临床观察[J].中国误诊学杂志,2008(05):1042-1043.
898. 黄玉明,杨建琼,刘君.中药汤剂加入西药联合保留灌肠治疗慢性溃疡性结肠炎临床研究[J].四川中医,2008(10):64-65.
899. 陈白莉,高翔,张芳宾,杨莉,杨荣萍,胡品津.双料喉风散灌肠治疗溃疡性直肠炎的随机对照临床研究[J].胃肠病学,2009,14(09):532-535.
900. 农子彪.口服柳氮磺胺吡啶加中药保留灌肠治疗溃疡性结肠炎30例疗效观察[J].内科,2009,4(06):879-880.
901. 刁玉明.中西医结合治疗溃疡性结肠炎的疗效观察[J].中国中西医结合消化杂志,2009,17(04):264-265.
902. 刘屹. 丹参注射液对溃疡性结肠炎血小板活化（瘀血）状态干预效果的实验研究与临床观察[D].长春中医药大学,2009.
903. 刘忠. 中西医结合治疗溃疡性结肠炎32例临床观察[J]. 江苏中医药,2009,41(9):41-41.
904. 刘爱云.锡类散混合液灌肠治疗溃疡性结肠炎的护理体会[J].中国现代医生,2009,47(21):141-142.
905. 刘龙发,缪林.中西医结合治疗溃疡性结肠炎55例[J].现代中西医结合杂志,2009,18(06):624.
906. 覃艳梅.中药灌肠治疗溃疡性结肠炎55例临床疗效观察[J].中国医药导报,2009,6(16):126+129.
907. 吴汝伟.中西医结合治疗49例慢性溃疡性结肠炎疗效观察[J].医学信息,2009,22(06):977-978.
908. 周小军,周怀力,徐晓华.锡类散、甲硝唑联合地塞米松治疗溃疡性结肠炎疗效观察[J].中国误诊学杂志,2009,9(11):2567-2568.
909. 周金林.自拟中药煎剂灌肠合柳氮磺胺吡啶治疗溃疡性结肠炎疗效观察[J].安徽中医学院学报,2009,28(01):22-23.
910. 唐永先.双黄鱼酱液保留灌肠治疗溃疡性结肠炎的观察与护理[J].当代护士(专科版),2009(12):10-12.
911. 孙勇. 中西医结合治疗溃疡性结肠炎27例[J]. 吉林中医药,2009,29(10):851-852.
912. 孙岩,盖永鸿.中西医结合治疗溃疡性结肠炎临床研究[J].中国实用医药,2009,4(08):140-141.
913. 孙忠杰.中西医结合治疗慢性非特异性溃疡性结肠炎疗效观察[J].河北中医,2009,31(01):84-85.
914. 宋传亮,霍秀杰. 中西医结合治疗溃疡性结肠炎37例[J]. 山东中医杂志,2009,28(1):50-51.
915. 崔彦红. 中西结合治疗溃疡性结肠炎临床观察[J]. 山西中医,2009, (S1):43-44.
916. 张志福,聂小正. 中西药灌肠治疗慢性溃疡性结肠炎136例[J]. 中外医疗,2009,28(28):28-28.
917. 张杰.中西医结合治疗溃疡性结肠炎40例[J].河南中医,2009,29(05):492.
918. 彭月芹,倪秀军,杨耀文,裴俊清,唐秀丽,许艳花.生化汤合桃花汤加味治疗轻中度溃疡性结肠炎45例[J].陕西中医,2009,30(09):1133-1134.
919. 徐岩,王启远,许贤姬,等. 丹参川芎嗪注射液联合治疗120例溃疡性结肠炎临床观察[J]. 中国民康医学,2009,21(13):1536,1538.
920. 徐庆杰. 中西医内外合治溃疡性结肠炎42例[J]. 河南大学学报（医学版）,2009,28(4):297-298.
921. 徐爱凤. 中西医结合治疗溃疡性结肠炎45例疗效观察[J]. 山西中医学院学报,2009,10(3):48-49.
922. 戴玲,刘青. 中药灌肠在慢性溃疡性结肠炎中的应用及护理[J]. 国际护理学杂志,2009,28(3):428-729.
923. 戴美兰. 中药热敷治疗溃疡性结肠炎的疗效观察及护理[J]. 中国民族民间医药,2009,18(13):172-173.
924. 房栩丞,王婴. 结肠宁汤灌肠治疗溃疡性结肠炎临床观察[J]. 中医药临床杂志,2009,21(6):553-554.
925. 曹化云. 中西医结合治疗溃疡性结肠炎35例经验[J]. 中国现代药物应用,2009,3(18):142-143.
926. 朱勇,谢会忠. 美沙拉嗪口服联合锡类散灌肠治疗溃疡性结肠炎的随机对照研究[J]. 新疆医科大学学报,2009,32(10):1459-1461.
927. 李二虎. 加味七味白术散联合柳氮磺胺吡啶治疗溃疡性结肠炎53例[J]. 河南中医,2009,29(10):1021-1021.
928. 李保良,刘友章,罗仁,李文娇.“益脾清肠汤”配合西药治疗UC 30例及对血清SOD和MDA的影响[J].江苏中医药,2009,41(01):23-24.
929. 李建平,马艳华,蒋文明.辨证分型联合西药灌肠治疗慢性溃疡性结肠炎42例[J].陕西中医,2009,30(09):1149-1151.
930. 李慧.中西医结合治疗溃疡性结肠炎疗效探讨[J].医药论坛杂志,2009,30(24):18-19.
931. 李永祥. 中西医结合治疗溃疡性结肠炎42例疗效观察[J]. 世界中西医结合杂志,2009,4(5):350-351.
932. 杨崇河,刘福明. 经内镜中西药物治疗溃疡性结肠炎临床研究[J]. 辽宁中医药大学学报,2009,11(8):95-96.
933. 杨红梅,张光奇.中西医结合治疗溃疡性结肠炎[J].贵阳中医学院学报,2009,31(02):46-47.
934. 林晓红,刘秋江.中西医结合治疗慢性非特异性溃疡性结肠炎128例[J].辽宁中医药大学学报,2009,11(08):170-171.
935. 林松,刘莹莹,郭秀珠.溃疡性结肠炎临床治疗体会[J].河北医学,2009,15(06):703-705.
936. 柏林,白光. 中西医结合治疗溃疡性结肠炎临床观察[J]. 实用中医内科杂志,2009,23(3):56.
937. 欧少福,张长欣.中西医结合治疗慢性复发型溃疡性结肠炎60例疗效观察[J].中医药导报,2009,15(06):33-34.
938. 熊建立. 中药保留灌肠结合西药治疗溃疡性结肠炎42例疗效观察[J]. 中医药导报,2009,15(11):29-31.
939. 王冬梅,张新广,陈凯,杨清.针灸配合中西药保留灌肠治疗溃疡性结肠炎[J].实用药物与临床,2009,12(03):224-225.
940. 王琼,杨晓钟,马刚. 丹参注射液对溃疡性结肠炎患者P-选择素的影响及疗效观察[J]. 中国医师进修杂志,2009,32(4):59-60.
941. 王继宁.中药灌肠联合口服西药治疗溃疡性结肠炎99例[J].陕西中医,2009,30(03):302-303.
942. 田自力,康小红,路玉香,徐世聪,孟杰,赵淑琴.穴位埋线联合柳氮磺胺吡啶治疗溃疡性结肠炎的疗效观察[J].医学研究与教育,2009,26(04):36-37.
943. 石湘波. 柳氮磺胺吡啶联用中药灌肠治疗溃疡性结肠炎61例临床观察[J]. 浙江临床医学,2009,11(9):944-945.
944. 程芳,李国栋.中西医结合治疗活动期溃疡性结肠炎临床研究[J].中国中医药信息杂志,2009,16(S1):41-42.
945. 罗云鹏,孙慧,赵鸣雁.中西医结合治疗72例溃疡性结肠炎的临床观察[J].黑龙江医药,2009,22(04):538-539.
946. 聂珍静,裴树丰.中药水煎剂联合麦滋林-S灌肠治疗溃疡性结肠炎56例[J].中国中西医结合消化杂志,2009,17(03):202-203.
947. 肖仲清,陈小英.参苓白术散配合中药保留灌肠治疗脾胃虚弱型溃疡性结肠炎73例疗效观察[J].河南中医,2009,29(09):870-871.
948. 苏建春,于云华,塔衣尔江,等. 中西医结合治疗溃疡性结肠炎40例疗效观察[J]. 中外健康文摘,2009,6(26):121-121.
949. 覃春荣.中西医结合治疗慢性溃汤性结肠炎39例临床观察[J].结直肠肛门外科,2009,15(05):319-320.
950. 覃艳梅.中药灌肠治疗溃疡性结肠炎55例临床疗效观察[J].中国医药导报,2009,6(16):126+129.
951. 赵春明. 中西医结合治疗溃疡性结肠炎的临床观察[J]. 临床和实验医学杂志,2009,8(5):117.
952. 罗云鹏,孙慧,赵鸣雁. 中西医结合治疗72例溃疡性结肠炎的临床观察[J]. 黑龙江医药,2009,22(4):538-539.
953. 邓健敏,韩宇斌,陈建林,陈锦锋.中西医治疗溃疡性结肠炎30例效果观察[J].中国实用医药,2009,4(17):118-119.
954. 郑惠梅,张洪霞.中西医结合治疗溃疡性结肠炎[J].中国民康医学,2009,21(12):1386-1386
955. 郑郸鄯. 中西医结合治疗溃疡性结肠炎[J]. 中国民族民间医药,2009,18(20):154-154.
956. 郝立智.中西医治疗32例溃疡性结肠炎的临床观察[J].中国现代药物应用,2009,3(03):58-59.
957. 钟莹,林娟.中西药结合灌肠治疗溃疡性结肠炎的疗效观察及护理[J].广东医学院学报,2009,27(02):224-225.
958. 陈家培.中西药内服结合中药灌肠治疗溃疡性结肠炎疗效观察[J].辽宁中医杂志,2009,36(01):98.
959. 章可谓.中西医结合治疗溃疡性结肠炎36例临床体会[J].中国中医急症,2009,18(12):2057-2058.
960. 高国仿,文荣群,李明.中西医结合治疗慢性溃疡性结肠炎临床观察[J].湖北中医杂志,2009,31(08):35-36.
961. 魏红兵.复方三黄汤保留灌肠治疗溃疡性结肠炎76例观察[J].中国医药导报,2009,6(12):86.
962. 黄捷平,周槐娜,喻永明.中药灌肠联合柳氮磺胺嘧啶口服治疗溃疡性结肠炎疗效观察[J].江西医药,2009,44(06):597-598.
963. 丁医峰.美沙拉嗪联合中药灌肠治疗溃疡性结肠炎临床疗效观察[J].中国药物与临床,2014,14(02):254-255.
964. 代文英,梁佳勐,许梦珂.中西医结合治疗溃疡性结肠炎80例疗效观察[J].内蒙古中医药,2014,33(25):37.
965. 任建国,刘娣,白文广.中西结合治疗出血性溃疡性结肠炎疗效观察[J].中国现代药物应用,2014,8(17):118-119.
966. 任毅. 针灸健脾补肾法治疗溃疡性结肠炎的临床研究[D].成都中医药大学,2014.
967. 何飞龙,金玉弟,袁金仁,张永力,柳峰.康复新液、云南白药联合柳氮磺吡啶保留灌肠治疗溃疡性结肠炎[J].长春中医药大学学报,2014,30(01):114-116.
968. 候志刚. 联合用药治疗溃疡性结肠炎临床疗效[J]. 医学信息,2014(26):191-191.
969. 侯朝英. 血竭地榆合剂灌肠联合美沙拉秦口服下调IL-17对溃疡性结肠炎黏膜愈合的影响[D].北京中医药大学,2014.
970. 侯杰.中西医结合治疗56例溃疡性结肠炎的临床疗效观察[J].现代诊断与治疗,2014,25(17):3915-3916.
971. 公建庄,杜晨旭.口服美沙拉嗪联合康复新、锡类散保留灌肠治疗轻中度远段溃疡性结肠炎的疗效观察[J].中国社区医师,2014,30(09):62-63.
972. 冯建荣,王斌,魏连刚,徐大超.中西医结合治疗溃疡性结肠炎疗效观察[J].山西中医,2014,30(05):25-26.
973. 刘宏晶.肠炎宁胶囊联合美沙拉嗪治疗溃疡性结肠炎的疗效研究[J].中医药学报,2014,42(04):142-145.
974. 刘小云.中西医结合治疗溃疡性结肠炎100例临床观察[J].内蒙古中医药,2014,33(16):71.
975. 刘彦晶,冷炎,金学洙,刘铁军.行气活血化瘀法对溃疡性结肠炎患者血清TNF-α和IL-6的影响[J].中国老年学杂志,2014,34(17):4753-4755.
976. 杜宏女.中西药保留灌肠治疗溃疡性结肠炎疗效观察及护理[J].中国中医急症,2009,18(09):1561-1562.
977. 刘海泉.灌肠配合西药治疗溃疡性结肠炎60例[J].中国中医药现代远程教育,2014,12(22):43-44.
978. 刘继威. 中西医结合治疗大肠湿热型溃疡性结肠炎疗效评价及相关免疫学指标的观察[D]. 天津中医药大学,2014.
979. 刘彦晶,冷炎,金学洙,刘铁军.行气活血化瘀法对溃疡性结肠炎患者血清TNF-α和IL-6的影响[J].中国老年学杂志,2014,34(17):4753-4755.
980. 厉琴,王莹,李静茹,汪银莹,罗红来,张莉,陶亮亮,仇学明,樊荣.美沙拉嗪肠溶片口服联合锡类散保留灌肠治疗溃疡性结肠炎的疗效观察[J].中国实用医药,2014,9(30):22-23.
981. 吴昊. 清肠化湿方治疗溃疡性结肠炎临床疗效观察及对NF-κB、caspase-3和肠黏膜屏障的影响[D]. 江苏:南京中医药大学,2014.
982. 周位远. 愈疡消溃方治疗溃疡性结肠炎活动期30例[J]. 中国中医药现代远程教育,2014,12(23):31-32.
983. 唐伟节.中药保留灌肠治疗溃疡性结肠炎64例疗效观察[J].四川中医,2014,32(09):94-96.
984. 姜康树,张飞龙.中西医结合治疗溃疡性结肠炎40例临床观察[J].国医论坛,2014,29(03):51-52.
985. 杜山鹏,侯媛,陈宝和.美沙拉嗪配伍中药保留灌肠治疗溃疡性结肠炎的临床观察[J].中医临床研究,2012,4(11):53-54.
986. 孙现周.综合疗法治疗溃疡性结肠炎的临床疗效观察[J].临床合理用药杂志,2014,7(13):49-50.
987. 安宇.中西医结合治疗溃疡性结肠炎效果观察[J].中国医刊,2014,49(10):69-70.
988. 宋丽琴,王国夫.中药灌肠治疗溃疡性结肠炎对炎性相关因子的影响[J].中华医院感染学杂志,2014,24(02):414-415+500.
989. 尤占永. 建中清肠汤联合糖皮质激素治疗溃疡性结肠炎疗效观察[J]. 医学信息,2014(19):625-626.
990. 巩艳春.宁肠方敷脐配合柳氮磺吡啶治疗溃疡性结肠炎的疗效观察[J].云南中医学院学报,2014,37(04):73-74.
991. 师桂英,王正亮,王蓓.半夏泻心汤联合美沙拉嗪灌肠对溃疡性结肠炎患者血清炎症因子的影响[J].中医药信息,2014,31(05):113-115.
992. 席玉红,党中勤,李严生,王红霞.中医综合疗法联合辨证施护治疗溃疡性结肠炎60例观察[J].中医药导报,2014,20(05):110-112.
993. 常东,刘子志,汪栋材,李键,冯春霞,李辉,何方.溃结灵联合柳氮磺胺吡碇片对大肠湿热型溃疡性结肠炎患者NO及IL-13的影响[J].中国中医急症,2014,23(03):432-433+482.
994. 廖莉.愈疡汤联合美沙拉嗪治疗溃疡性结肠炎疗效分析[J].内蒙古中医药,2014,33(18):46.
995. 张友发,纪春江,刘建新. 蒙脱石散与云南白药灌肠联合美沙拉嗪治疗溃疡性结肠炎的临床研究[J]. 医学信息,2014(21):117-117.
996. 张咏华.中药汤剂联合美沙拉嗪治疗溃疡性结肠炎临床效果分析[J].深圳中西医结合杂志,2014,24(07):38-39.
997. 张惠玲,杨玉杰,赵金媛.美沙拉嗪联合中药保留灌肠治疗溃疡性结肠炎疗效观察[J].护理研究,2014,28(29):3672-3673.
998. 张春林,张秀琴,李生鹏,刘娟燕,成灵芝.中药灌肠联合西药治疗溃疡性结肠炎随机平行对照研究[J].实用中医内科杂志,2014,28(10):129-131.
999. 张理顺,李心茹,鲁海燕.葛根芩连汤合美沙拉嗪对溃疡性结肠炎湿热内蓄证患者血清IL-6、TNF-α的影响[J].湖南中医杂志,2014,30(07):64-65.
1000. 张都全.健脾固涩汤治疗溃疡性结肠炎临床疗效观察[J].实用中西医结合临床,2014,14(05):82-83.
1001. 张静华,杨军.中西医结合治疗溃疡性结肠炎36例疗效观察[J].新中医,2014,46(04):69-70.
1002. 张鹏飞. 复方黄柏液辅助治疗溃疡性结肠炎的临床试验[D]. 南昌大学,2014.
1003. 徐建. 愈疡汤联合美沙拉嗪治疗溃疡性结肠炎41例[J]. 中国中医药现代远程教育,2014(17):52-53.
1004. 敬满芳,武洁.中西医结合治疗溃疡性结肠炎30例疗效观察[J].内蒙古中医药,2014,33(35):79.
1005. 文莉,刘金莲. 中西医结合治疗慢性溃疡性结肠炎22例临床疗效观察研究[J]. 中国保健营养（下旬刊）,2014,24(7):4326.
1006. 曹郑云. 中西医结合治疗溃疡性结肠炎45例疗效观察[J]. 中医临床研究,2014(25):76-77,79.
1007. 朱海燕,陈国旗. 清溃灌肠方联合美沙拉嗪治疗小儿溃疡性结肠炎临床观察[J]. 四川中医,2014,32(5):102-103.
1008. 朱莉,张丽,马蕾. 中西药结合治疗溃疡性结肠炎40例临床观察[J]. 西部医学,2014,26(10):1335-1336,1339.
1009. 李有成,严文有,陈俊英,蔡菊梅.加味阳和四神汤/浸膏剂治疗慢性溃疡性结肠炎疗效观察[J].西部中医药,2014,27(05):104-106.
1010. 李淑英,陈婉,杨佐琴,付雪.健脾疏肝煎治疗肝郁脾虚型活动期溃疡性结肠炎的临床研究[J].上海中医药杂志,2014,48(01):36-38.
1011. 李点玲.美沙拉嗪联合中药灌肠治疗溃疡性结肠炎的临床效果观察[J].当代医学,2014,20(11):145-146.
1012. 李爱丽,张俊仲,周冰.肠道水疗合云南白药和锡类散结肠灌注治疗溃疡性结肠炎20例总结[J].湖南中医杂志,2014,30(03):18-19+22.
1013. 李艳杰,郭雅明. 溃疡性结肠炎的临床治疗分析[J]. 世界最新医学信息文摘（电子版）,2014(17):19-20.
1014. 李鹏,杨少军,王培宏. 逍遥散联合美沙拉嗪肠溶片治疗溃疡性结肠炎随机平行对照研究[J]. 实用中医内科杂志,2014,28(5):85-87.
1015. 李鹏程,李士瑾. 中西医结合治疗溃疡性结肠炎40例[J]. 中医研究,2014,27(6):25-27.
1016. 杜雪峰,陆庆革. 白头翁汤加减联合美莎拉嗪肠溶片治疗慢性非特异性溃疡性结肠炎80例临床观察[J]. 河北中医,2014(9):1345-1346.
1017. 杨万玲,张宇文. 美沙拉嗪联合益脾汤治疗溃疡性结肠炎临床分析[J]. 内蒙古中医药,2014,33(24):31-32.
1018. 杨梦凡. 推拿结合西药治疗对湿热型溃疡性结肠炎缓解期的疗效观察[D]. 江苏:南京中医药大学,2014.
1019. 杨莹莹.白头翁汤加味治疗溃疡性结肠炎82例临床研究[J].中医临床研究,2014,6(06):69-70.
1020. 林坚.中西药联用治疗溃疡性结肠炎疗效及对炎性因子的影响[J].新中医,2014,46(04):66-68.
1021. 林晋濠. 肠愈宁对溃疡性结肠炎活动期患者血清C-反应蛋白影响的临床观察[D].黑龙江中医药大学,2014.
1022. 柏莉娟,李海洋.外用溃疡散联合地塞米松灌肠治疗溃疡性结肠炎疗效观察[J].中医药临床杂志,2014,26(10):1040-1041.
1023. 梁桂美,张丽玲.柳氮磺砒啶联合玉屏风颗粒治疗溃疡性结肠炎的临床研究[J].中国医药指南,2014,12(11):51-52.
1024. 檀雪松,谢勇,曲建忠.中西医结合治疗溃疡性结肠炎50例临床疗效观察[J].中医临床研究,2014,6(36):113-115.
1025. 欧阳春喜.自拟芪术健脾消疡汤治疗溃疡性结肠炎236例[J].中国中医药现代远程教育,2014,12(13):139-140.
1026. 江玉山. 中西医结合治疗中重度溃疡性结肠炎的疗效观察[J]. 医学信息,2014(14):433-433.
1027. 沈洪,顾培青,成家飞,等. 清肠化湿法联合美沙拉嗪治疗活动期溃疡性结肠炎临床疗效观察[C]. //第二十六届全国中西医结合消化系统疾病学术会议论文集. 2014:636-640.
1028. 焦建华,陆彩霞,孙怡燕. 穴位强化埋线疗法联合口服美沙拉嗪治疗溃疡性结肠炎疗效观察[J]. 山东医药,2014,54(11):42-43.
1029. 牛彤,王艳霞. 中西医结合治疗溃疡性结肠炎60例临床观察[J]. 北华大学学报（自然科学版）,2014(4):511-513.
1030. 王佳林. 乌梅丸治疗溃疡性结肠炎的疗效观察及对血清IL-4和IL-8水平的影响[J]. 海南医学院学报,2014,20(1):80-82.
1031. 王倩倩,俞林,甄朋超,王丰.葛根芩连汤灌肠联合柳氮磺吡啶肠溶片口服治疗慢性非特异性溃疡性结肠炎32例临床观察[J].河北中医,2014,36(07):1023-1024.
1032. 王巧玲.康复新液灌肠联合美沙拉嗪治疗溃疡性结肠炎临床观察[J].山西医药杂志,2014,43(05):539-540.
1033. 王朝阳,冯燕,冯乐,杨雅娟,李良霄,朱琳,陈玲玲.针灸结合中西药物综合干预慢性溃疡性结肠炎疗效观察[J].西部医学,2014,26(02):199-200+203.
1034. 王美玉. 肠炎康联合美沙拉嗪治疗溃疡性结肠炎[J]. 医学信息,2014(20):455-455.
1035. 申艳梅,夏进娥. 大桃花汤联合西药保留灌肠治疗溃疡性结肠炎疗效观察[J]. 实用中医药杂志,2014(6):522-522,523.
1036. 皮茂. 结肠宁保留灌肠治疗湿热内蕴型溃疡性结肠炎32例总结[J]. 中医药导报,2014,20(4):133-134.
1037. 盘宏. 中西药灌肠治疗溃疡性结肠炎的疗效观察及护理[J]. 内蒙古中医药,2014,33(24):151-152.
1038. 祁双林. 中西医结合治疗慢性溃疡性结肠炎20例疗效观察[J]. 湖南中医杂志,2014,30(8):62-63.
1039. 程胜平. 中西药物保留灌肠治疗溃疡性结肠炎的临床疗效及对热休克蛋白70的影响[J]. 中国中医药科技,2014,21(3):295-296.
1040. 窦英磊,马俊杰. 肺肠同治指导下中医药对柳氮磺胺吡啶灌肠治疗溃疡性结肠炎增效解毒研究[J]. 辽宁中医杂志,2014,41(7):1423-1425.
1041. 肖雪霞,陈晖. 自拟中药清肠愈疡汤结合西药治疗溃疡性结肠炎疗效观察[J]. 光明中医,2014(8):1710-1711.
1042. 胡加兵. 清肠解毒方联合手术治疗非特异性溃疡性结肠炎疗效观察[J]. 新中医,2014,46(12):67-68.
1043. 腾阳,董卫国,汤绍迁.败酱草合剂联合密波电针治疗溃疡性结肠炎40例[J].河南中医,2014,34(12):2362-2365.
1044. 苏攀. 加味柴芍六君颗粒治疗肝郁脾虚型溃疡性结肠炎的临床研究[D]. 广西中医药大学,2014.
1045. 范维玲. 中药颗粒保留灌肠治疗溃疡性结肠炎的疗效观察及护理体会[J]. 光明中医,2014(9):1975-1976.
1046. 葛飞,朱时林,肖明兵,马小平,季瑜,王亚军,田祖成,代海峰,于琴.基于脑肠轴研究电针联合药物治疗UC的作用机制——附31例临床资料[J].江苏中医药,2014,46(12):65-67.
1047. 董万青. 隔药饼灸治疗脾肾阳虚型溃疡性结肠炎的临床观察[D].北京中医药大学,2014.
1048. 蒋丽琴. 健脾清热化湿法治疗脾虚湿热型溃疡性结肠炎缓解期的疗效观察[D].南京中医药大学,2014.
1049. 蒋波涛,张朋,李荣华,周州,陶阳,晏玉婷.中西医结合治疗溃疡性结肠炎的临床疗效评价[J].中医临床研究,2014,6(03):7-10.
1050. 薛会才,何勤泉,何青.中西医结合治疗慢性溃疡性结肠炎68例临床观察[J].四川中医,2014,32(12):99-100.
1051. 薛雷,张大伟,姜广兵.美沙拉嗪联合中药汤剂治疗溃疡性结肠炎疗效观察[J].现代中西医结合杂志,2014,23(13):1420-1422.
1052. 袁恩.白头翁汤加味治疗溃疡性结肠炎的疗效及作用机制[J].现代中西医结合杂志,2014,23(09):955-957.
1053. 谢中华,汪铁军,郑元秀,黄芳芳,王珠美.舒血宁注射液联合常规疗法治疗活动期溃疡性结肠炎患者疗效观察[J].中国中西医结合杂志,2014,34(10):1164-1167.
1054. 谢函君,叶志伟.中西医结合治疗溃疡性结肠炎临床观察[J].湖北中医药大学学报,2014,16(06):72-73.
1055. 谭簪,曾松林,王迪.美沙拉嗪联合康复新液灌肠对溃疡性结肠炎的临床研究[J].中国医药指南,2014,12(20):262-263.
1056. 赵建杰,张爱军,陆霞,单海燕,常玉洁.耳穴贴压配合中药灌肠治疗湿热内蕴型慢性溃疡性结肠炎32例[J].上海中医药杂志,2014,48(12):42-43.
1057. 赵新芳,孙维华,甘霞,林红,周云.化毒愈肠方治疗溃疡性结肠炎40例[J].中国实验方剂学杂志,2014,20(22):216-219.
1058. 赵登清,魏华.中西医结合治疗溃疡性结肠炎60例观察[J].实用中医药杂志,2014,30(07):646.
1059. 边毅,边颖,周宏伟,任静茹,谢鹏.中药灌肠联合西药口服对溃疡性结肠炎的疗效观察[J].临床合理用药杂志,2014,7(10):78-79.
1060. 邓平安.痛泻要方加味联合柳氮磺胺吡啶治疗溃疡性结肠炎40例[J].河南中医,2014,34(10):1994-1995.
1061. 邓海燕.中西医结合治疗慢性溃疡性结肠炎60例观察[J].实用中医药杂志,2014,30(01):32.
1062. 郑发鹃,张苏闽.不同治疗方案对活动期溃疡性结肠炎患者生存质量的影响[J].现代中西医结合杂志,2014,23(32):3535-3537+3540.
1063. 郑培奋,李希诗,朱琴,何巧娜,杨维佳.连理汤加味对活动期溃疡性结肠炎IL-33表达的影响及临床疗效[J].中华中医药学刊,2014,32(10):2472-2474.
1064. 郑常耀. 中西医结合治疗溃疡性结肠炎的临床研究[J]. 医学美学美容（中旬刊）,2014(5):91-92.
1065. 郑笑涛. 溃结方灌肠联合美沙拉嗪口服治疗溃疡性结肠炎48例[J]. 中国中医药现代远程教育,2014(17):56-57.
1066. 金春兰,全珠华.肠炎康联合美沙拉嗪治疗溃疡性结肠炎[J].中国卫生标准管理,2014,5(16):37-38.
1067. 金晓东.中西医结合治疗和护理70例溃疡性结肠炎体会[J].湖南中医杂志,2014,30(11):120-121.
1068. 陆玥琳,沈洪,姚宏凤,杨旭.清肠化湿方联合美沙拉嗪颗粒对溃疡性结肠炎患者血浆及肠黏膜组织IL-17的影响[J].中国中西医结合杂志,2014,34(10):1160-1163.
1069. 陈军,鲁磊,刘志国.加味附子理中汤治疗脾肾阳虚型溃疡性结肠炎疗效观察[J].中国中西医结合消化杂志,2014,22(10):624-625+627.
1070. 陈利坚,叶斌.加味芍药汤联合美沙拉嗪治疗溃疡性结肠炎的疗效及对患者血清炎症因子的影响[J].中华中医药学刊,2014,32(05):1042-1043.
1071. 陈华伟,钟军华,袁勇.愈疡消溃方治疗溃疡性结肠炎活动期近期疗效观察[J].中国实验方剂学杂志,2014,20(10):192-195.
1072. 陈忠伟.芍芪椿皮汤治疗大肠湿热型溃疡性结肠炎的临床疗效观察[J].实用中西医结合临床,2014,14(11):16-17.
1073. 陈晓丽. 溃结宁灌肠治疗湿热蕴结型溃疡性结肠炎临床疗效观察[D].南京中医药大学,2014.
1074. 陈欣.中西药联合治疗溃疡性结肠炎20例[J].中国药业,2014,23(06):83-84.
1075. 陈泽云.六合汤加减治疗慢性复发型溃疡性结肠炎的临床价值[J].深圳中西医结合杂志,2014,24(03):108-109.
1076. 陈红.参苓白术散保留灌肠联合美沙拉嗪口服治疗慢性非特异性溃疡性结肠炎疗效观察[J].河北中医,2014,36(11):1643-1644.
1077. 陈红.参芪五味子片联合柳氮磺胺吡啶结肠溶胶囊治疗慢性非特异性溃疡性结肠炎疗效观察[J].河北中医,2014,36(03):419-420.
1078. 雷开秀,李志.中医综合治疗溃疡性结肠炎32例[J].中国中医药现代远程教育,2014,12(10):29-31.
1079. 章新华,叶毅,陈光辉,邓建忠,董瑶.中西医结合治疗慢性非特异性溃疡性结肠炎临床观察[J].实用中西医结合临床,2014,14(04):39-40.
1080. 马亦旻.美沙拉嗪、思密达联合锡类散灌肠治疗溃疡性结肠炎疗效观察[J].现代中西医结合杂志,2014,23(23):2558-2560.
1081. 马俊杰,窦英磊,张以来,张静静.肺肠同治指导下中医药联合SASP灌肠对AUC Th1/Th2调节作用研究[J].世界科学技术-中医药现代化,2014,16(04):769-773.
1082. 高虹.中西医结合治疗慢性溃疡性结肠炎47例[J].河南中医,2014,34(11):2251.
1083. 魏丽萍,宋红旗,叶道冰.治疗脾肾阳虚型溃疡性结肠炎30例[J].中国中医药现代远程教育,2014,12(15):129-130.
1084. 黎炳全.肠炎康联合美沙拉嗪治疗溃疡性结肠炎40例临床观察[J].中国民族民间医药,2014,23(20):71.
1085. 伍学兵.乌梅丸联合美沙拉嗪治疗溃疡性结肠炎疗效观察[J].亚太传统医药,2015,11(09):134-135.
1086. 伍裕.中西医结合治疗溃疡性结肠炎疗效评价[J].内蒙古中医药,2015,34(08):59.
1087. 何显,张清泉,王真,王文智,王丽华.中药灌肠对轻、中度溃疡性结肠炎患者的疗效及脂联素的调节作用[J].世界华人消化杂志,2015,23(17):2834-2838.
1088. 何显,王文智,苏心爱,张清泉,王丽华.中药灌肠对溃疡性结肠炎患者Th1/Th2免疫调节的影响[J].四川中医,2015,33(04):64-66.
1089. 余淑娇,柯诗文,李晴,吴锐,鄢毅,李国贤.活血清肠方对溃疡性结肠炎的临床研究[J].世界中西医结合杂志,2015,10(03):342-345.
1090. 傅晓丹,王旭东,张少华.中药滴注灌肠加参苓白术散治疗溃疡性结肠炎(脾虚湿盛型)的疗效观察[J].深圳中西医结合杂志,2015,25(06):72-73.
1091. 党惠娇,蔡少华,张在厚,陈伟伟,雷励.中药灌肠联合美沙拉嗪对溃疡性结肠炎患者血小板功能的影响[J].现代生物医学进展,2015,15(22):4314-4316.
1092. 冯德魁,黄少鹏,贾恒,陈美雄.中西医结合治疗湿热内蕴型溃疡性结肠炎的临床疗效观察[J].广州中医药大学学报,2015,32(02):243-246.
1093. 冯振仁.中西医结合治疗溃疡性结肠炎60例临床观察[J].中国医药科学,2015,5(14):32-34+53.
1094. 向四国,仰涢霞,陆忠红.难治性溃疡性结肠炎患者临床诊断与治疗研究[J].中华损伤与修复杂志(电子版),2013,8(06):29-31+38.
1095. 刘亮,李春阳. 芍药汤口服配合美沙拉秦治疗溃疡性结肠炎60例疗效观察[C]. //中华中医药学会肛肠分会2015年学术年会暨全国流调行业发布会论文集. 2015:549-551.
1096. 刘玉珠,徐芳,熊腊根. “通调气血,寒热并用”法结合西药治疗溃疡性结肠炎的疗效观察[J]. 中国中西医结合消化杂志,2015,23(7):493-495.
1097. 刘长生,张后振,木合塔尔·阿尤甫.加味白头翁汤保留灌肠治疗溃疡性结肠炎临床观察[J].新疆中医药,2015,33(01):17-18.
1098. 刘颖.加味芍药汤联合美沙拉嗪治疗溃疡性结肠炎的疗效及对患者镜下黏膜病变的影响[J].临床医学工程,2015,22(01):61-62.
1099. 刘军楼,沈洪,顾培青,郑凯,刘亚军,沈天华.清肠化湿灌肠方联合美沙拉嗪治疗远端溃疡性结肠炎的临床疗效[J].世界华人消化杂志,2015,23(35):5715-5721.
1100. 卞银燕.联用葛根芩连汤和美沙拉嗪治疗溃疡性结肠炎的效果分析[J].当代医药论丛,2015,13(17):182-183.
1101. 吕生辉,冯德魁.化毒愈疡方保留灌肠治疗轻、中度溃疡性结肠炎43例[J].中国实验方剂学杂志,2015,21(02):217-220.
1102. 吴和木,林鸿霞,赖斌斌. 中西医结合治疗溃疡性结肠炎306例[C]. //第二十七届全国中西医结合消化系统疾病学术会议论文集. 2015:422-422.
1103. 吴小玲,何义波,吴瑜梅. 柳氮磺吡啶片联合中药保留灌肠治疗溃疡性结肠炎的效果观察[J]. 中国当代医药,2015(26):158-160.
1104. 吴艳. 中西医结合治疗慢性溃疡性结肠炎临床研究[J]. 吉林医学,2015,36(4):674.
1105. 姬亚超. 云南白药胶囊治疗类似于溃疡性结肠炎的“久痢”的疗效观察[J]. 医学信息,2015(23):211-211.
1106. 孔庆波. 中西医结合治疗溃疡性结肠炎的疗效观察[J]. 中西医结合心血管病电子杂志,2015(8):11-12.
1107. 孙斌. 针灸对溃疡性结肠炎患者T细胞亚群影响观察[J]. 亚太传统医药,2015,11(24):104-105.
1108. 宁玉凤,杨翠兰,曹生海. 愈肠煎保留灌肠联合美沙拉嗪治疗溃疡性结肠炎60例[J]. 中国实验方剂学杂志,2015,21(13):199-203.
1109. 宗伟,孙燕,刘贵生.穴位埋线对溃疡性结肠炎的疗效评价及对NF-κB和5-LOX表达的影响[J].四川中医,2015,33(10):156-160.
1110. 崔童.肠安康胶囊联合美沙拉嗪治疗溃疡性结肠炎疗效观察[J].西部中医药,2015,28(01):78-80.
1111. 张孟林,谢守勇. 中西医结合治疗溃疡性结肠炎34例临床观察[J]. 中国肛肠病杂志,2015,35(9):32-33.
1112. 张文杰. 探讨中西医结合治疗慢性溃疡性结肠炎的临床疗效[J]. 医药前沿,2015(23):126-127.
1113. 张明辉. 中药保留灌肠联合美沙拉嗪治疗溃疡性结肠炎效果观察[J]. 中医临床研究,2015(31):117-118.
1114. 张爱军,赵建杰,陆霞,常玉洁.耳穴贴压法联合改良愈疡汤灌肠治疗溃疡性结肠炎44例[J].河南中医,2015,35(08):1987-1988.
1115. 张玉伟. 中药灌肠结合美沙拉嗪用于溃疡性结肠炎治疗临床分析[J]. 医学信息,2015(48):68-68.
1116. 张礼辉. 中西药结合治疗重度溃疡性结肠炎临床观察[J]. 新中医,2015,47(9):79-80.
1117. 张维,李红. 中药灌肠联合美沙拉嗪治疗80例溃疡性结肠炎临床观察[J]. 中国现代药物应用,2015(15):174-175,176.
1118. 张茹,李春耕. 中西医结合治疗慢性非特异性溃疡性结肠炎50例[J]. 河南中医,2015,35(6):1399-1400.
1119. 张金涛,王晓锋,陈浩漩. 中西医结合药物治疗慢性溃疡性结肠炎疗效观察[J]. 医学综述,2015(14):2683-2684,2689.
1120. 张静燕,邱允忠,刘庆晔.联用复方参柏合剂保留灌肠法与美沙拉嗪治疗溃疡性结肠炎的效果分析[J].当代医药论丛,2015,13(21):186-187.
1121. 徐大志,王艳,张维,桂壮.清肠化湿法治疗87例活动期溃疡性结肠炎的临床研究[J].中国中西医结合消化杂志,2015,23(11):820-823.
1122. 徐宝琪.参苓白术散联合美沙拉嗪治疗对溃疡性结肠炎IL-17、TNF-α及IL-23影响研究[J].现代诊断与治疗,2015,26(02):298-299.
1123. 徐爱华,陈红. 参苓白术散保留灌肠联合美沙拉嗪口服对溃疡性结肠炎患者结肠粘膜的影响[J]. 国际检验医学杂志,2015,36(0):56-58.
1124. 徐生志. 柳氮磺吡啶肠溶片联合丹参川芎嗪注射液治疗活动期溃疡性结肠炎临床观察[J]. 新中医,2015,47(6):56-58.
1125. 徐纪文. 参苓白术散联合美沙拉嗪治疗脾胃气虚型溃疡性结肠炎疗效观察[J]. 四川中医,2015,33(10):60-61.
1126. 徐莹.康复新液联合糖皮质激素保留灌肠治疗溃疡性结肠炎158例临床疗效观察[J].中医临床研究,2015,7(12):72-73.
1127. 李世晏,郝淑莉,冯长华.自拟红藤汤佐治慢性溃疡性结肠炎60例临床观察[J].中国中西医结合消化杂志,2015,23(08):584-585.
1128. 李晓玲.芍药汤加减配合西药灌肠治疗溃疡性结肠炎49例临床疗效[J].北方药学,2015,12(07):50-51.
1129. 李红玲.内服与外用药物联合治疗溃疡性结肠炎的临床效果[J].世界最新医学信息文摘,2015,15(07):4-5.
1130. 李魁.参苓白术散联合美沙拉嗪治疗脾胃气虚型溃疡性结肠炎疗效及对血清细胞因子的影响[J].现代中西医结合杂志,2015,24(22):2467-2469.
1131. 杜明,王秋萍,张锦明.中药免煎颗粒联合美莎拉嗪灌肠液治疗溃疡性结肠炎38例[J].河南中医,2015,35(02):390-391.DOI:
1132. 杨斌,王碧霞.美沙拉嗪栓联合穴位贴治疗溃疡性结肠炎中期效果探讨[J].西部中医药,2015,28(05):112-114.
1133. 杨新建,张飞龙.中西医结合治疗慢性溃疡性结肠炎60例[J].河南中医,2015,35(02):392-393.
1134. 杨桂香. 中西医结合治疗溃疡性结肠炎临床护理研究[J]. 医学信息,2015,28(52):229.
1135. 杨洁,王忠明,党治军,冷光现,李访斌,赵爱霞.针、药、穴联合治疗溃疡性结肠炎的疗效观察[J].卫生职业教育,2015,33(12):144-146.
1136. 杨珠莹,谢齐贵,夏亮,陈军贤,赵秋枫.中西医结合治疗溃疡性结肠炎的临床研究[J].中华中医药学刊,2015,33(11):2814-2816.
1137. 杨竞男,王垂杰.白头翁汤联合美沙拉嗪治疗溃疡性结肠炎随机平行对照研究[J].实用中医内科杂志,2015,29(12):84-85.
1138. 林泽青,章振宏,林文杰.中西医结合治疗慢性非特异性溃疡性结肠炎的临床研究[J].中医临床研究,2015,7(24):97-99.
1139. 林瑞芳,黎友隆,蔡悦,吕保阶,李佩侃.溃疡性结肠炎配合中药散剂保留灌肠疗效观察[J].内蒙古中医药,2015,34(11):106-107.
1140. 梁秀琴,刘瑞芳.中药灌肠治疗溃疡性结肠炎的疗效观察及护理[J].基层医学论坛,2015,19(03):428-429.
1141. 梁艳妮,周首邦,梁秋明.中西药合用治疗溃疡性结肠炎疗效观察[J].实用中医药杂志,2015,31(05):421-422.
1142. 杨东辉,宋盘格,王彦丽,张晓华.清热祛湿法联合西药治疗溃疡性结肠炎疗效观察[J].四川中医,2015,33(06):95-97.
1143. 洪子夫. 连芍灌肠剂治疗轻、中度UC临床疗效观察及实验研究[D].中国中医科学院,2015.
1144. 曹永胜,陈银环.中西药保留灌肠治疗溃疡性结肠炎疗效观察[J].辽宁中医杂志,2006(08):1001-1002.
1145. 王卫峰,周峰,冯玉良,姚惠,杨维佳,郑培奋.连理汤加味对活动期溃疡性结肠炎TNF-α和IL-10表达的影响[J].中华中医药学刊,2015,33(02):381-383.
1146. 王晓宏.中西医结合治疗慢性溃疡性结肠炎46例观察[J].实用中医药杂志,2015,31(07):643.
1147. 姜康树,张飞龙.中西医结合治疗溃疡性结肠炎40例临床观察[J].国医论坛,2014,29(03):51-52.
1148. 王海英.中药灌肠结合柳氮磺胺吡啶对溃疡性结肠炎的效果观察[J].中国继续医学教育,2015,7(18):218-220.
1149. 王海麟.中西医结合治疗难治性溃疡性结肠炎临床观察[J].内蒙古中医药,2015,34(08):80-81.
1150. 祝三秀,邵晓燕. 美沙拉嗪联合云南白药灌肠对溃疡性结肠炎患者的临床观察及护理[J]. 医学信息,2015(45):175-176.
1151. 秦兴亚.联用参苓白术颗粒和美沙拉嗪治疗溃疡性结肠炎的效果观察[J].当代医药论丛,2015,13(13):169.
1152. 秦菲. 中西医结合治疗溃疡性结肠炎及调控细胞因子研究[D].南京中医药大学,2015.
1153. 程燕红,郭玉宏. 地塞米松+云南白药保留灌肠对溃疡性结肠炎治疗效果的观察[J]. 医药前沿,2015(10):186-187.
1154. 胡华.云南白药与康复新液灌肠联合美沙拉嗪治疗溃疡性结肠炎的临床观察[J].中国实用医药,2015,10(31):195-196.
1155. 胡美英,姜凌,熊萍香,杨德平.中医护理干预溃疡性结肠炎40例[J].中国中医药现代远程教育,2015,13(15):108-110.
1156. 胡雪庐,毛国章.中西药联用治疗激素依赖性溃疡性结肠炎43例[J].中国中医药科技,2015,22(01):105-106.
1157. 范宇锋,顾艳宏.自拟解毒凉血汤灌肠联合美沙拉嗪栓治疗直肠型溃疡性结肠炎患者临床疗效分析[J].中国现代医生,2015,53(09):111-115.
1158. 葛畅,邝靖. 中药灌肠联合微生态制剂治疗溃疡性结肠炎临床研究[J]. 中国基层医药,2015,22(1):93-95.
1159. 葛飞,季瑜,马小平,王亚军,田祖成,代海峰,于琴.针药结合治疗溃疡性结肠炎的临床效果观察[J].中国当代医药,2015,22(06):164-165+168.
1160. 董佳,王爱玲.美沙拉嗪片联合白头翁汤灌肠治疗溃疡性结肠炎40例[J].中医外治杂志,2015,24(03):41.
1161. 解刘松,韦怡.中西医结合治疗湿热内蕴型溃疡性结肠炎的疗效观察[J].中国药物经济学,2015,10(S2):50-51.
1162. 赵建勋,李霞,吴大英.自拟中药内服联合保留灌肠对溃疡性结肠炎IL-10、IL-18的影响[J].陕西中医,2015,36(06):709-710.
1163. 赵忠玮. 四神丸、曲美布汀及双歧三联活菌胶囊联合治疗肾阳虚型溃疡性结肠炎疗效观察[J]. 中国基层医药,2015(19):2954-2955.
1164. 赵杏芳,王志明. 柳氮磺吡啶联合葛根苓连汤保留灌肠治疗溃疡性结肠炎的疗效观察[J]. 国际护理学杂志,2015(15):2159-2160.
1165. 辛群,孙擎,葛现才,张勤,齐元福.参苓白术散与美沙拉嗪对溃疡性结肠炎患者血清IL-17、IL-23及TNF-α水平的影响[J].现代生物医学进展,2015,15(09):1663-1665+1670.
1166. 邱慧卿,徐建光.苦参素注射液联合中药灌肠治疗溃疡性结肠炎临床观察[J].新中医,2015,47(11):69-71.
1167. 郭雨浩,柏树纲.中西医结合治疗脾肾阳虚型溃疡性结肠炎疗效观察[J].山西中医,2015,31(08):21-22.
1168. 金银顺.超微七味白术散治疗溃疡性结肠炎临床观察[J].湖北民族学院学报(医学版),2015,32(02):74+77.
1169. 阮俊姣. 中西医结合灌肠治疗直乙型溃疡性结肠炎的临床效果分析[J]. 医学信息,2015(24):81-82.
1170. 陈凯军,李彩丽.针药结合治疗活动期湿热型溃疡性结肠炎疗效观察[J].中国针灸,2015,35(05):435-438.
1171. 陈建辉.中西医结合治疗溃疡性结肠炎的临床疗效观察[J].现代医药卫生,2015,31(03):415-416.
1172. 陈桂仙.中西药合用治疗溃疡性结肠炎疗效观察[J].实用中医药杂志,2015,31(10):924.
1173. 陈淑君.穴位埋线治疗溃疡性结肠炎的疗效及对抗中性粒细胞胞浆抗体的影响[J].中医临床研究,2015,7(03):78-80.
1174. 陈裕章.中西医结合治疗慢性非特异性结肠炎的临床疗效[J].中国中医药现代远程教育,2015,13(21):70-71.
1175. 章慧宏,吴元祥.从脾虚肝郁论治慢性溃疡性结肠炎52例临床体会[J].中医临床研究,2015,7(25):99-100.
1176. 章真,赵宝林.益气清肠活血方治疗慢性非特异性溃疡性结肠炎40例临床观察[J].河北中医,2015,37(08):1167-1168+1172.
1177. 马占欣.美沙拉嗪与中药保留灌肠联用治疗溃疡性结肠炎临床探讨[J].结直肠肛门外科,2015,21(S1):20-21.
1178. 高英杰,王英南,赵佛军,刘宝林,胡林山,张学诚,贾国璞,刘经州,姚芳,刘卫民.中西药结合治疗溃疡性结肠炎的临床观察[J].河北医学,2015,21(07):1187-1189.
1179. 高英杰,刘卫民,吴松柏,赵佛军,姚芳,王英南.中药保留灌肠治疗溃疡性结肠炎30例肠镜疗效分析[J].承德医学院学报,2015,32(06):493-495.
1180. 高英杰,王英南,赵佛军,姚芳,刘卫民.中药保留灌肠加口服美沙拉嗪肠溶片对溃疡性结肠炎患者血小板状态的影响[J].中国老年学杂志,2015,35(19):5547-5548.
1181. 魏少兴. 清肠化浊汤联合美沙拉嗪治疗慢性溃疡性结肠炎的临床研究[D].河北医科大学,2015.
1182. 鲍新坤,林爱珍,孙光军,等. 加味补阳还五汤联合美沙拉嗪治疗溃疡性结肠炎的疗效观察[J]. 中西医结合研究,2015(3):142-143.
1183. 黄宽忠. 溃结灵方治疗脾虚湿热型轻中度活动期溃疡性结肠炎的临床研究[D]. 江苏:南京中医药大学,2015.
1184. 黄绿澜. 中药灌肠联合西药治疗溃疡性结肠炎临床疗效观察[J]. 世界临床医学,2015,9(4):47.
1185. 尹会芬. 中西医结合治疗活动期溃疡性结肠炎的临床疗效观察[D]. 湖北:湖北中医药大学,2016.
1186. 付怀成. 中西医结合治疗溃疡性结肠炎41例临床观察[J]. 江苏中医药,2016,48(3):41-42.
1187. 付智钢. 中西医结合治疗溃疡性结肠炎的临床观察[J]. 光明中医,2016,31(18):2718-2719.
1188. 伍先华. 中药保留灌肠联合半夏泻心汤加味治疗溃疡性结肠炎40例临床观察[J]. 中国民族民间医药,2016,25(13):78-79.
1189. 佘德军,滑永志,樊欣钰. 达康方保留灌肠中西医结合治疗溃疡性结肠炎疗效观察[J]. 世界最新医学信息文摘（连续型电子期刊）,2016,16(87):175-175,176.
1190. 保继琼. 葛根芩连汤合异功散加味治疗溃疡性结肠炎大肠湿热证的临床研究[D]. 甘肃中医药大学,2016.
1191. 凌线荣. 中药热敷联合西药内服治疗溃疡性结肠炎疗效观察[J]. 广西中医药,2016,39(3):57-58.
1192. 刘亮,李春阳,韦全剑. 芍药汤配合美沙拉嗪治疗溃疡性结肠炎疗效观察[J]. 中国肛肠病杂志,2016,36(6):37-38.
1193. 刘刚,周莉,熊国卫,等. 灸药合治溃疡性结肠炎25例临床观察[J]. 江苏中医药,2016,48(5):66-67.
1194. 刘启明.美沙拉嗪联合加味甘草泻心汤治疗溃疡性结肠炎的疗效观察[J].深圳中西医结合杂志,2016,26(01):50-51.
1195. 刘彦晶,冷炎,金学洙,刘铁军.化瘀宁肠愈疡方灌肠对溃疡性结肠炎患者血清相关因子含量的影响[J].中医临床研究,2016,8(08):54-56.
1196. 刘彩军.溃疡性结肠炎葛根芩连汤合白头翁汤治疗临床疗效观察[J].药品评价,2016,13(B12):249-249.
1197. 刘晓娟.盐熨配合中药直肠滴入治疗溃疡性结肠炎[J].光明中医,2016,31(4):527-528.
1198. 刘朝霞,黄岩.穴位埋线联合美沙拉嗪治疗溃疡性结肠炎临床观察[J].现代中医药,2016,36(03):18-21.
1199. 刘汉雄.美沙拉嗪联合中药保留灌肠治疗溃疡性结肠炎临床研究[J].亚太传统医药,2016,12(06):142-143.
1200. 刘淑玮,程胜平.自拟肠康散灌肠对溃疡性结肠炎患者炎性因子和氧化应激水平的影响[J].中医药导报,2016,22(03):75-77.
1201. 刘纪炎. 中药保留灌肠配合美沙拉嗪治疗溃疡性结肠炎40例临床观察[J]. 中国肛肠病杂志,2016,36(4):43-44.
1202. 刘菲.康复新液、云南白药保留灌肠与温和灸联合美沙拉嗪治疗溃疡性结肠炎的疗效观察和护理[J].内蒙古中医药,2016,35(01):108-109.
1203. 卢宏福. SASP、九香虫颗粒口服并隔姜艾灸治疗脾肾阳虚型溃疡性结肠炎疗效观察[J]. 中国肛肠病杂志,2016,36(6):35-36.
1204. 双文武.中西医结合治疗溃疡性结肠炎疗效观察[J].内蒙古中医药,2016,35(10):53-54.
1205. 吴世铖,陈璇,郑烈,唐志鹏.美沙拉嗪联合中药灌肠治疗脾虚湿热型溃疡性结肠炎48例临床观察[J].新中医,2016,48(03):49-51.
1206. 吕红伟.中西医结合治疗60例难治性溃疡性结肠炎的临床观察[J].中西医结合研究,2016,8(06):301-302.
1207. 周正华,岳妍,王威,王红霞,康洪昌,魏景景,胡蓆宝,冀建斌.青赤散保留灌肠联合口服美沙拉嗪治疗溃疡性结肠炎临床观察[J].天津中医药,2016,33(12):719-722.
1208. 姜小燕,龚晓莉,揭建勇.穴位埋线联合美沙拉嗪治疗溃疡性结肠炎的临床观察[J].当代医学,2016,22(23):129-130.
1209. 宋凯丽. 加味升阳益胃汤治疗溃疡性结肠炎缓解期脾肾阳虚证的临床观察[D].山西省中医药研究院,2016.
1210. 屈沂.半夏泻心汤口服配合灌肠对溃疡性结肠炎患者血清细胞因子、生活质量的影响[J].陕西中医,2016,37(04):440-441.
1211. 屈海涛.消癌平注射液在溃疡性结肠炎(UC)治疗中的临床观察[J].中国卫生标准管理,2016,7(11):150-152.
1212. 张宝元,李文宇,王晋阳,刑一,徐三鹏.姜石颗粒联合美沙拉嗪肠溶片治疗脾胃虚弱型溃疡性结肠炎30例[J].中医研究,2016,29(04):7-9.
1213. 张文艳. 康复新液联合锡类散治疗溃疡性结肠炎的临床效果评价[J]. 医药前沿,2016,6(14):208-209.
1214. 张艳君,常玉洁.荆芥连翘汤加减治疗溃疡性结肠炎32例疗效观察[J].中医药导报,2016,22(21):64-66.
1215. 曾晓梅.参苓白术散加减配合中药灌肠治疗溃疡性结肠炎46例[J].北方药学,2016,13(02):97.
1216. 朱丽丽,郭海,赵晓峰,刘志.中西医结合治疗寒热错杂型溃疡性结肠炎30例[J].河南中医,2016,36(11):1995-1997.
1217. 朱颖玲,唐晓华,吴红铃.中西医结合疗法治疗溃疡性结肠炎的疗效及对凝血功能、炎性因子水平的影响分析[J].中国医药科学,2016,6(23):82-86.
1218. 李刚. 溃疡性结肠炎结合中药治疗的临床观察[C]. //第三届全国中西医结合治疗肝病临床经验学术研讨会论文集. 2016:264-265.
1219. 李发军,李鹂.柳氮磺胺吡啶联合中药灌肠治疗溃疡性结肠炎疗效观察[J].中外医学研究,2016,14(01):141-143.
1220. 李学军,陈花,吴婧,金月萍.脾胃培源灌肠方治疗溃疡性结肠炎35例[J].光明中医,2016,31(11):1594-1595.
1221. 李榕萍.中西医结合治疗溃疡性结肠炎的效果观察[J].中国当代医药,2016,23(17):148-151.
1222. 杨梅,吴东.黄芩汤颗粒剂联合美沙拉秦对溃疡性结肠炎的临床疗效研究[J].中国中西医结合消化杂志,2016,24(03):221-223.
1223. 毛从俊. 中药灌肠治疗溃疡性结肠炎的疗效分析[J]. 国际医药卫生导报,2016,22(1):111-113.
1224. 汪方园. 健脾清肠颗粒对溃疡性结肠炎（脾虚湿热证）西医常规疗法的增效作用研究[D]. 河南中医药大学,2016.
1225. 游龙,秦晔.美沙拉嗪联合中药汤剂治疗溃疡性结肠炎的效果分析[J].中医临床研究,2016,8(08):86-87.
1226. 游龙,秦晔.中西医结合治疗溃疡性结肠炎的疗效评价[J].中国医药指南,2016,14(10):195-196.
1227. 王光昀,李波.中药保留灌肠联合西药治疗溃疡性结肠炎大肠湿热型30例[J].中医研究,2016,29(07):8-10.
1228. 王强.湿润烧伤膏灌肠治疗溃疡性结肠炎34例临床观察[J].中国民间疗法,2016,24(12):32-33.
1229. 王成文,薛霖,王玉梅.参苓白术散口服联合柳氮磺胺吡啶灌肠治疗溃疡性结肠炎的临床观察[J].青海医药杂志,2016,46(08):64-66.
1230. 王振疆. 探讨中药结肠宁对溃疡性结肠炎患者肠黏膜屏障和肠道微生态的影响[D].暨南大学,2016.
1231. 王晶.美沙拉嗪口服加中药灌肠治疗48例溃疡性结肠炎临床观察[J].中医临床研究,2016,8(04):105-106.
1232. 王金明.用乌梅丸联合西药治疗溃疡性结肠炎的疗效研究[J].当代医药论丛,2016,14(02):111-112.
1233. 胡倩. 口服美沙拉嗪加中药灌肠方白头翁汤加减治疗活动期溃疡性结肠炎（湿热内蕴证）的临床疗效观察[D].西南医科大学,2016.
1234. 胡响当,陈艳,罗敏,李梅,杨宗亮.复方芩柏颗粒剂保留灌肠治疗湿热下注型溃疡性结肠炎临床研究[J].亚太传统医药,2016,12(06):148-149.
1235. 胡天穹,邵中一,王曜.清肠化湿方联合美沙拉嗪治疗活动期轻中度溃疡性结肠炎的临床疗效[J].世界华人消化杂志,2016,24(36):4824-4829.
1236. 苏强,牛立军,段荣欣,刘启旺,刘江波.白葛芩连汤治疗直肠型溃疡性结肠炎107例[J].河南中医,2016,36(03):534-535.
1237. 许家珂,王云峰,赵坚敏.康复新液口服治疗轻中度炎症性肠病的临床效果观察[J].中国当代医药,2016,23(34):126-129.
1238. 贺秀萍,邵方萍. 中西药混合液保留灌肠治疗溃疡性肠炎52例临床观察[J]. 养生保健指南,2016(50):70.
1239. 贺静,王海燕. 中西医结合保留灌肠治疗溃疡性结肠炎的疗效观察[J]. 临床心身疾病杂志,2016,22(z2):66-67.
1240. 贾晓归.补脾清肠汤治疗溃疡性结肠炎35例临床观察[J].中医临床研究,2016,8(27):87-88.
1241. 赵敏,王振军,许艳春.龙血竭散联合美沙拉嗪缓释片灌肠治疗溃疡性结肠炎[J].长春中医药大学学报,2016,32(01):115-117.
1242. 赵新芳,甘霞,林红,周云.化毒愈肠方内服和灌肠治疗溃疡性结肠炎的疗效及对血清IL-22和IL-23的影响[J].中国实验方剂学杂志,2016,22(04):178-181.
1243. 赵社海,王春光,龙枚飞.美沙拉嗪联合中药灌肠治疗溃疡性结肠炎的效果分析[J].大家健康(学术版),2016,10(09):127-128.
1244. 赵喜.中医结合柳氮磺胺吡啶治疗溃疡性结肠炎的临床研究[J].大家健康(学术版),2016,10(02):122.
1245. 邓台燕,全大祥,吴彬.复方黄柏液联合美沙拉嗪对溃疡性结肠炎及肠道菌群和血清炎症因子的影响[J].中国现代医学杂志,2016,26(09):124-127.
1246. 邓文珺,马颖才,马丽莉.美沙拉嗪联合丹参注射液治疗溃疡性结肠炎的疗效及对患者炎性因子与凝血指标的影响[J].世界华人消化杂志,2016,24(03):462-466.
1247. 郭保君,陆鹏,张镭潇,冒冬冬,余思奕,申治富,任毅,钟贞,胡卡明,胡幼平.针灸健脾补肾法治疗溃疡性结肠炎疗效观察[J].四川中医,2016,34(05):182-185.
1248. 郭雨浩. 自拟健脾助运汤治疗脾虚湿蕴型溃疡性结肠炎疗效观察[D].辽宁中医药大学,2016.
1249. 钟燕华,皮厚远.中医辨证联合莫西沙星、柳氮磺吡啶治疗溃疡性结肠炎效果观察[J].现代消化及介入诊疗,2016,21(03):387-389+393.
1250. 陆硕蕾. 中药保留灌肠治疗活动期溃疡性结肠炎的疗效观察[D].南京中医药大学,2016.
1251. 陈慕豪,王立恒.中药内服加灌肠治疗大肠湿热型溃疡性结肠炎临床研究[J].新中医,2016,48(03):47-49.
1252. 陈战斌,杨晓蓓,王环.解毒化瘀中药联合西药治疗溃疡性结肠炎疗效及对炎性反应及高凝状态的影响[J].现代中西医结合杂志,2016,25(17):1851-1854.
1253. 陈淑妮,姚民武.白头翁汤加减联合美沙拉嗪治疗湿热型溃疡性结肠炎疗效观察[J].临床医学研究与实践,2016,1(11):94-95.
1254. 雷晓梅,许洪明,温瑶明,等. 芍药汤加减加美沙拉嗪灌肠联合美沙拉嗪口服治疗溃疡性结肠炎临床观察[J]. 中国肛肠病杂志,2016,36(9):35-37.
1255. 韩丰,冀子中. 观察美沙拉嗪肠溶片联合加味芍药汤对溃疡性结肠炎患者肠黏膜修复作用[J]. 新中医,2016,48(4):56-58.
1256. 章小平,查安生,丁小丽,邹晓华.健脾解毒化瘀方灌肠对溃疡性结肠炎患者T细胞亚群的研究[J].中华中医药学刊,2016,34(02):396-398.
1257. 马坤,何晨,何涛. 中西医结合治疗慢性溃疡性结肠炎临床研究[J]. 实用中医药杂志,2016,32(6):560-560,561.
1258. 马洁,席作武. 四神片联合美沙拉嗪治疗脾肾阳虚型溃疡性结肠炎的临床疗效[J]. 中国肛肠病杂志,2016,36(2):48-49.
1259. 高红涛. 中西医结合治疗溃疡性结肠炎的疗效[J]. 临床研究,2016,24(10):52-53.
1260. 黄丽强,杨梦凡,田君,李立文,徐敏,顾飞,张国华.穴位贴敷联合西药治疗溃疡性结肠炎疗效观察及机制研究[J].亚太传统医药,2016,12(22):87-89.
1261. 黄卫清,廖为民.半夏泻心汤加减联合柳氮磺胺吡啶治疗溃疡性结肠炎临床研究[J].亚太传统医药,2016,12(04):130-131.
1262. 黄群,冷玉杰.中药血竭保留灌肠治疗溃疡性结肠炎[J].吉林中医药,2016,36(10):1001-1004.
1263. 黎家楼,余白桦,梁景星.温胃饮加味联合蒙脱石散、美沙拉嗪治疗溃疡性结肠炎疗效观察[J].现代中西医结合杂志,2016,25(19):2108-2110.
1264. 于姣,何瑾瑜,张蕾.中药灌肠对溃疡性结肠炎患者的临床疗效及中性粒细胞与淋巴细胞比值的影响[J].中国卫生检验杂志,2017,27(20):2965-2967.
1265. 于姣,何瑾瑜,张蕾.中药灌肠对溃疡性结肠炎患者的临床疗效及其对肠道菌群的影响[J].中国微生态学杂志,2017,29(11):1323-1325+1329.
1266. 伊巴代提·阿西木. 中西医结合治疗慢性非特异性溃疡性结肠炎46例观察[J]. 医药前沿,2017,7(12):108-109.
1267. 刘丹. 加味柴胡疏肝散颗粒对溃疡性结肠炎气滞湿阻证的临床观察[D]. 广西中医药大学,2017.
1268. 刘刚,周莉,熊国卫,宋泽汎,王增增,王玖.中西医结合治疗溃疡性结肠炎32例[J].中医临床研究,2017,9(22):76-78.
1269. 刘晶. 中药保留灌肠联合美沙拉嗪治疗溃疡性结肠炎的临床观察[D]. 温州医科大学,2017.
1270. 刘志威,王学群,李甜甜.槐花散对溃疡性结肠炎急性期糖皮质激素用量影响及疗效[J].牡丹江医学院学报,2017,38(04):69-71+66.
1271. 向桢,安运锋,曹立军,贺学强,李霞.中西医结合治疗溃疡性结肠炎疗效观察[J].实用中医药杂志,2017,33(01):55-56.
1272. 吕立群.补脾胃泻阴火升阳汤配合热敏灸法治疗溃疡性结肠炎临床观察[J].四川中医,2017,35(06):95-97.
1273. 吴和木,林鸿霞,辛金坤,等. 中西医结合治疗溃疡性结肠75例临床观察[C]. //2017年中医肛肠国际交流大会论文集. 2017:242-242.
1274. 周志军,王威. 薏苡附子败酱散对脾肾阳虚型溃疡性结肠炎患者的临床疗效观察:附80例报告[J]. 中国中西医结合急救杂志,2017,24(4):419-422.
1275. 周莉,刘刚,熊国卫,宋泽汎,王增增,王玖.芍药汤加减联合艾灸对溃疡性结肠炎患者结肠黏膜修复的影响[J].中国处方药,2017,15(05):93-94.
1276. 唐景荣. 乌梅丸加味治疗溃疡性结肠炎（寒热错杂证）的临床研究[D].长春中医药大学,2017.
1277. 孙春全,李金玲,董甜甜,庞亚铮,杨继国.督脐灸疗法配合美沙拉嗪治疗溃疡性结肠炎脾肾阳虚证临床观察[J].中国中西医结合消化杂志,2017,25(07):525-528.
1278. 孙显军.芍药汤加减联合西药治疗溃疡性结肠炎疗效观察[J].现代中西医结合杂志,2017,26(04):436-438.
1279. 孙漫原. 调补脾胃中药联合常规西药对脾胃虚弱型溃疡性结肠炎患者中医证候改善及减轻黏膜损伤程度影响[J]. 中国保健营养,2017,27(18):4.
1280. 孙芳,钱海华,颜帅. 大黄附子汤联合双歧三联活菌胶囊治疗慢性复发型结肠炎的临床研究[J]. 时珍国医国药,2017,28(8):1918-1920.
1281. 干丹,韩昌鹏,冯卓,金炜,杨豪杰,李盈,崔灿,梁榕钰,王云云,王振宜.祛瘀生新方联合美沙拉秦治疗轻中度溃疡性结肠炎的临床研究[J].上海中医药杂志,2017,51(08):54-57.
1282. 张丽娟.白头翁加甘草阿胶汤加减联合美沙拉嗪治疗溃疡性结肠炎的临床观察[J].中国初级卫生保健,2017,31(07):80-81.
1283. 张伟,于俊兰.美沙拉嗪肠溶片联合中药保留灌肠治疗远端活动期轻中度溃疡性结肠炎的临床观察[J].包头医学,2017,41(04):5-6.
1284. 张声生,杨雪,赵鲁卿,沈洪,赵文霞,唐志鹏,谢胜,谢静.清热除湿中药灌肠方治疗溃疡性结肠炎近期疗效的观察[J].中国中西医结合消化杂志,2017,25(06):401-405.
1285. 张嵩,王文光.疏肝健脾化瘀方联合柳氮磺吡啶治疗慢性溃疡性结肠炎46例[J].中国中医药科技,2017,24(03):364-365.
1286. 张萍. 复方黄柏液保留灌肠对溃疡性结肠炎患者的疗效观察[J]. 大家健康（下旬版）,2017,11(6):27-28.
1287. 强玉婷. 化浊解毒愈肠方联合美沙拉嗪治疗溃疡性结肠炎的临床观察[D]. 河北:河北医科大学,2017.
1288. 戈瑞,范娴娴,景建中,等. 复方黄柏液涂剂保留灌肠治疗溃疡性结肠炎临床观察[J]. 中国保健营养,2017,27(27):253.
1289. 支亚军,孟凡冰.复方苦参肠溶胶囊与谷氨酰胺肠溶胶囊联合美沙拉嗪治疗溃疡性结肠炎的疗效比较[J].世界中医药,2017,12(11):2655-2658.
1290. 智建文,李华山,杨祎,何颖华,贾菲,王晓锋.美沙拉嗪肠溶片联合中药灌肠方治疗湿热内蕴型溃疡性结肠炎疗效评价[J].蚌埠医学院学报,2017,42(09):1193-1195.
1291. 曹姣姣. 涤肠愈疡汤灌肠联合西药治疗溃疡性结肠炎（大肠湿热证）的临床观察[D].湖北中医药大学,2017.
1292. 曾云.葛根芩连汤加白芨灌肠治疗慢性溃疡性结肠炎急性发作的疗效分析[J].中外医疗,2017,36(23):180-181+184.
1293. 李丰全. 中西医结合的治疗56例溃疡性结肠炎患者的临床分析[J]. 家庭医药,2017(3):67-68.
1294. 李昶. 美沙拉嗪对溃疡性结肠炎患者的临床疗效及对ESR、PLT、D-二聚体的影响研究[J]. 中国医药指南,2017,15(19):103-104.
1295. 李晓玲.益气活血解毒法抗溃疡性结肠炎复发的分析[J].内蒙古中医药,2017,36(10):74.
1296. 李英,纪群.溃疡性结肠炎患者采用美沙拉嗪结合灌肠的疗效评价[J].临床医药文献电子杂志,2017,4(46):9056.
1297. 杜国新.柳氮磺胺吡啶联合中药灌肠对溃疡性结肠炎患者肠道微生态和屏障功能的影响[J].内科,2017,12(05):689-691.
1298. 杨合功.中西医结合治疗溃疡性结肠炎的疗效对比分析[J].实用中西医结合临床,2017,17(09):59-60.
1299. 杨周雨,贲定严,易展,何永恒.隔药饼灸治疗脾虚湿蕴证轻中度溃疡性结肠炎临床研究[J].中国中医药信息杂志,2017,24(04):32-35.
1300. 杨曼曼,罗雯鹏,李克亚,王真权.芩柏颗粒保留灌肠治疗湿热蕴结型溃疡性结肠炎37例临床观察[J].湖南中医杂志,2017,33(12):10-12.
1301. 杨磊,李莹,袁星星,李丹丹,王炳予,刘长发,张雅丽.青白灌肠液对活动期溃疡性结肠炎患者临床疗效及炎性因子、黏附因子的影响[J].中国中医急症,2017,26(07):1145-1148.
1302. 杨颖.中西医结合治疗溃疡性结肠炎临床观察[J].实用中医药杂志,2017,33(10):1174-1176.
1303. 柴娟娟.中西医结合治疗溃疡性结肠炎临床疗效观察[J].临床医学研究与实践,2017,2(23):104-105.
1304. 桑怡,刘庆生,李蕾,等. 中西医结合治疗湿热内蕴型溃疡性结肠炎临床效果观察[J]. 浙江临床医学,2017,19(1):41-42.
1305. 武赞仁,李丰林. 中药保留灌肠结合西药口服治疗脾肾阳虚型溃疡性结肠炎的临床研究[J]. 中国肛肠病杂志,2017,37(8):51-54.
1306. 沈莉,薛睿. 中西医结合保留灌肠治疗溃疡性结肠炎的疗效观察[J]. 家庭医药,2017(7):134-135.
1307. 浦丽超. 针灸结合中西医药物综合干预慢性溃疡性结肠炎的疗效观察[J]. 中国社区医师,2017,33(3):90-91.
1308. 王威,周至军. 芍药汤加减联合美沙拉嗪治疗溃疡性结肠炎的临床疗效观察[J]. 中国中西医结合急救杂志,2017,24(3):239-242.
1309. 王炳予,袁星星,刘长发,张雅丽.解毒化瘀汤治疗活动期溃疡性结肠炎的临床疗效及其对血清细胞因子和凝血指标的影响[J].上海中医药大学学报,2017,31(04):32-37.
1310. 王猛,蒋进广,管靖,罗倩.白头翁汤保留灌肠联合针刺治疗溃疡性结肠炎临床研究[J].中国中西医结合外科杂志,2017,23(06):645-648.
1311. 王皓霖,石立鹏,赵凤林,邬红霞,杨德钱,冉茂芳.加味芍药汤治疗溃疡性结肠炎湿热内蕴证32例疗效观察[J].湖南中医杂志,2017,33(11):11-14.
1312. 王筱华.云南白药辅助标准方案对老年溃疡性结肠炎患者疗效的影响[J].结直肠肛门外科,2017,23(04):514-517.
1313. 盛儒丹,石立鹏,张金龙,郑芳,杨德钱,冉茂芳,高淑容.加味芍药汤合美沙拉嗪治疗溃疡性结肠炎(湿热蕴结证)临床观察[J].中国中医急症,2017,26(09):1619-1622.
1314. 罗廷威,吴小莹.艾灸章门穴治疗溃疡性结肠炎疗效观察[J].上海针灸杂志,2017,36(10):1177-1180.
1315. 翦闽涛,宾东华,李逵,王爱华.参苓白术散加减方保留灌肠治疗溃疡性结肠炎30例疗效观察[J].湖南中医杂志,2017,33(02):48-50.
1316. 聂清海,郑春根.中药连理汤加味治疗活动期溃疡性结肠炎疗效观察及对患者TNF-α、IL-10表达的影响研究[J].数理医药学杂志,2017,30(11):1647-1649.
1317. 胡灿,方东,张继学,谭英斌.清热活血方灌肠治疗溃疡性结肠炎临床研究[J].亚太传统医药,2017,13(22):142-144.
1318. 袁代解,耿曙光,韦日娜,钟元帅,孙平良.安肠汤联合美沙拉嗪治疗溃疡性结肠炎的临床观察[J].光明中医,2017,32(02):263-265.
1319. 许晨,宇永军,张轶,林欣,李玉玮,张锡朋,江涛.葛根芩连五炭汤对溃疡性结肠炎患者炎症因子和氧化应激的影响[J].中医学报,2017,32(06):1067-1071.
1320. 谢妲.涩肠青黛散联合美沙拉嗪对溃疡性结肠炎患者炎症因子及氧化应激的影响[J].中医学报,2017,32(06):1063-1066.
1321. 费香勇.利湿和血汤辅助美沙拉嗪治疗溃疡性结肠炎疗效及对生活质量、炎症免疫细胞因子水平的影响[J].中国中医急症,2017,26(06):1097-1099.
1322. 赵莉,石志敏,李雪青.补脾胃泻阴火升阳汤联合美沙拉嗪治疗溃疡性结肠炎患者临床疗效观察[J].中国实验方剂学杂志,2017,23(14):188-192.
1323. 邱伟,方晓华,杨振斌,夏文娟,陈莉丽.中医外治法联合美沙拉嗪治疗溃疡性结肠炎32例临床研究[J].江苏中医药,2017,49(05):29-31.
1324. 邹百仓,秦斌,张莉,丁辉,王燕,张瑜,董蕾.肠炎灵I号灌肠治疗活动期溃疡性结肠炎临床研究[J].陕西中医,2017,38(11):1511-1512.
1325. 郗崇利.中西医结合治疗溃疡性结肠炎并发糖尿病疗效观察[J].糖尿病新世界,2017,20(06):97-99.
1326. 郭富成,张玲玲.消疡方灌肠联合美沙拉嗪治疗溃疡性结肠炎湿热内蕴证临床研究[J].中医学报,2017,32(09):1637-1640.
1327. 郭留霞.温阳法深度缓解炎症性肠病的临床研究[J].世界科学技术-中医药现代化,2017,19(12):2034-2039.
1328. 郭颂铭,宋献文.自拟中药方剂穴位贴敷结合美沙拉嗪栓治疗溃疡性结肠炎急性发作的疗效观察[J].中国中医急症,2017,26(05):883-886.
1329. 陈铭诗,杜立阳,张杰.复方青黛颗粒联合益生菌治疗溃疡性结肠炎的疗效观察[J].中国中西医结合消化杂志,2017,25(10):751-754.
1330. 陈浩,张波,徐速,卢芩,钱海华,曾莉.甘草泻心汤联合美沙拉嗪对溃疡性结肠炎的疗效及对血清炎症指标的影响[J].中药材,2017,40(02):475-478.
1331. 陈淑君,张双喜.葛根芩连汤联合柳氮磺胺吡啶治疗溃疡性结肠炎临床研究[J].陕西中医,2017,38(08):1110-1111.
1332. 韦丽兰,韦雄,南小利.白头翁汤联合西药治疗溃疡性结肠炎临床疗效观察[J].吉林医学,2017,38(04):711-712.
1333. 高戈,李师.益气活血汤联合美沙拉嗪治疗溃疡性结肠炎的临床疗效观察[J].中国现代药物应用,2017,11(03):125-127.
1334. 魏文先,翟玉云,邹颖.乌梅丸加减对溃疡性结肠炎患者炎性因子的影响[J].中医学报,2017,32(11):2208-2211.
1335. 龙晓斌,刘宏萍,贺海清,贺剑平,胡法清,余秋霞.穴位埋线联合美沙拉嗪对溃疡性结肠炎患者红细胞沉降率、D-二聚体和C反应蛋白的影响[J].医疗装备,2017,30(17):3-4.
1336. Xiao C, Bo C, Li ML, Li Z. Effects of the Treatment of Dong Medicine Five-Flavour Anti-Diarrhoea Soup Combined with Melalazine on Serum Complement C3, C4, Interleukin-23 and Interleukin-17 in Patients with Ulcerative Colitis. INDIAN JOURNAL OF PHARMACEUTICAL SCIENCES. 2022;84:324-328. doi:10.36468/pharmaceutical-sciences.spl.523
1337. 丁照亮, 杜少庆, 胡海华, 范宜堂, 都美杰. 正阳汤联合美沙拉嗪治疗溃疡性结肠炎52例疗效观察[J]. 中国肛肠病杂志. 2022;42(6):30-33.
1338. 付荣华, 郑勇, 郑春菊. 怡情止泻汤联合美沙拉嗪治疗溃疡性结肠炎临床效果观察 [J]. 中华中医药学刊. 2022;40(12):244-247.
1339. 代汝伟, 高志远, 刘秀丽, 王蕾, 徐智广. 加味白头翁汤治疗热毒炽盛型溃疡性结肠炎的疗效[J].西北药学杂志. 2022;37(4)2022334330.
1340. 代汝伟, 高志远, 王欣, 徐智广. 加味白头翁汤治疗热毒炽盛型急性溃疡性结肠炎的临床症状观察[J]. 中医临床研究. 2022;14(1)2022231678.
1341. 何巧飞, 何飞龙, 张永力, 徐秋霞. 基于内痈理论治疗活动期湿热毒证溃疡性结肠炎的疗效观察及其对炎性因子的影响[J].中国中医药科技. 2022;29(1)2022123535.
1342. 何飞龙, 何巧飞, 徐秋霞, 武彧竹. 清疡汤灌肠方联合美沙拉嗪肠溶片治疗溃疡性结肠炎(大肠湿热证)临床研究[J]. 特别健康. 2022;(23):263-264.
1343. 余利华, 杨东杰, 赵辉. 基于圆运动理论运用加味白头翁汤治疗溃疡性结肠炎大肠湿热型临床研究[J]. 广西中医药. 2022;45(01):8-11.
1344. 侯朝军. 柳氮磺胺吡啶联合康复新液对溃疡性结肠炎患者肠道黏膜NF-κB、TLR-4表达及免疫功能的影响[J].反射疗法与康复医学. 2022;3(10)2022452047.
1345. 刘俊杰, 高剑, 黎淑玲. 云南白药保留灌肠联合美沙拉嗪肠溶片+双歧杆菌三联活菌胶囊治疗溃疡性结肠炎的临床效果观察[J]. 结直肠肛门外科. 2022;28(02):138-142.
1346. 刘向龙, 杨玲, 安志恒, et al. 清热利湿法对湿热内蕴证溃疡性结肠炎疾病活动度及肠黏膜屏障的临床疗效研究[J]. 中国中西医结合消化杂志. 2022;30(08):585-590.
1347. 刘安. 中药保留灌肠联合美沙拉嗪口服治疗大肠湿热型溃疡性结肠炎的临床研究[D]. 硕士. 湖南中医药大学; 2022.
1348. 刘新红, 赖沁, 王志良, 杜薇, 叶茂. 痛泻要方联合美沙拉嗪肠溶片治疗肝郁脾虚型溃疡性结肠炎的临床观察[J]. 实用中西医结合临床. 2022;22(14):1-5+21.
1349. 刘晓彦. 康复新液联合美沙拉嗪治疗溃疡性结肠炎的效果探讨[J]. 中华养生保健·学术版. 2022;40(12)2022310371.
1350. 刘暘. 美沙拉嗪联合康复新液治疗溃疡性结肠炎的临床效果观察[J]. 基层医学论坛. 2022;26(2)2022160120.
1351. 刘英梅, 郭世宏, 倪福录. 白头翁汤配合西药治疗溃疡性结肠炎41例疗效观察[J].中国肛肠病杂志. 2023;43(1)2023233305.
1352. 华云玮, 林俊儒, 李放, 戴彦成. 健脾清肠汤对溃疡性结肠炎患者临床症状、结肠病变及免疫功能调节作用的研究[J]. 世界中西医结合杂志. 2022;17(10):2057-2061,2066.
1353. 卢朱霞, 毛细云, 周巧, 张贤琼. 四神丸合参苓白术散加减方联合美沙拉嗪栓治疗脾肾阳虚型溃疡性结肠炎临床疗效[J].中医药临床杂志. 2022;34(6)2022339708.
1354. 周新, 袁海鑫. 溃结灌肠汤联合美沙拉嗪肠溶片治疗溃疡性结肠炎的疗效及对改良Mayo评分、结肠镜检查评分的影响[J]. 中医研究. 2022;35(11):48-52.
1355. 周禹辰, 王佛有. 升阳健脾汤联合止痛散治疗溃疡性结肠炎的随机平行对照研究[J]. 中国中医药现代远程教育. 2022;20(13):74-76.
1356. 姚志刚, 杨维维. 锡类散灌肠联合美沙拉嗪治疗湿热型溃疡性结肠炎的临床疗效及对疾病活动指数的影响[J]. 中国处方药. 2022;20(9)2022444599.
1357. 孔庆羽, 胡慧. 仙芪固肠方联合美沙拉嗪治疗溃疡性结肠炎疗效观察[J]. 现代中西医结合杂志. 2022;31(19):2713-2716.
1358. 孙中, 王艳梅, 王媛, 艾江. 美沙拉嗪口服联合锡类散、L-谷氨酰胺、利多卡因保留灌肠治疗远端型溃疡性结肠炎的临床疗效[J].中国现代医生. 2022;60(3)2022184128.
1359. 孙志惠, 李忠卓. 加味香连丸联合美沙拉嗪治疗溃疡性结肠炎临床观察[J]. 山西中医. 2023;39(05):19-21.
1360. 孙成. 芪苍苦柏汤保留灌肠治疗溃疡性结肠炎（脾虚湿阻证）的临床研究[M]. 硕士. 山东中医药大学; 2022.
1361. 宋佳. 柴胡芍药汤加减联合美沙拉嗪肠溶片治疗溃疡性结肠炎的临床观察[J]. 内蒙古中医药. 2022;41(06):56-58.
1362. 宋贯霞, 丁城. 香连片联合柳氮磺吡啶肠溶片治疗溃疡性结肠炎大肠湿热证疗效观察[J]. 实用中医药杂志. 2023;39(1)2023181866.
1363. 崔大鹏, 杨建波. 安肠愈疡汤联合美沙拉嗪肠溶片治疗脾虚湿阻型缓解期溃疡性结肠炎患者的效果[J].中国民康医学. 2022;34(21)2023107080.
1364. 廖志远, 孙平良, 兰智妮, et al. 安肠汤联合常规治疗对活动期脾肾阳虚型溃疡性结肠炎患者的临床疗效[J]. 中成药. 2022;44(7)2022403482.
1365. 张丽慧, 段培芳, 张震, 曲丽丽. 疏肝解郁胶囊联合中药灌肠治疗溃疡性结肠炎患者的疗效观察[J].世界中西医结合杂志. 2022;17(10)2023154432.
1366. 张亚庆. 自拟中药灌肠方联合美沙拉嗪治疗溃疡性结肠炎的临床效果观察[J]. 中国现代药物应用. 2022;16(05):179-181.
1367. 张冬冬, 张艳君, 朱叶珊. 温针疗法联合西药治疗溃疡性结肠炎的疗效观察[J]. 上海针灸杂志. 2022;41(05):443-449.
1368. 张慧俭, 刘世举. 消溃汤加减治疗溃疡性结肠炎[J]. 中医学报. 2023;38(06):1315-1321.
1369. 张春霞, 黄志高. 隔附子饼灸联合美沙拉嗪治疗湿热型溃疡性结肠炎的临床观察[J]. 中国民间疗法. 2023;31(03):67-70.
1370. 张晓宇, 陈爱霞, 刘晓仪. 加味芍药汤联合美沙拉秦治疗湿热下注型溃疡性结肠炎临床研究[J].天津中医药. 2023;40(2)2023192333.
1371. 张洋. 口服“助阳通腑汤”治疗脾肾阳虚型溃疡性结肠炎的临床研究[M]. 硕士. 辽宁中医药大学; 2022.
1372. 张燕, 庞迪, 冯燕平. 复方黄柏液保留灌肠治疗溃疡性结肠炎的临床效果观察[J]. 实用中医内科杂志. 2022;36(06):51-53.
1373. 张雅丽, 袁星星, 王炳予, 卢科强. 大肠湿热证溃疡性结肠炎与外周血髓样树突状细胞表型的相关性及连草泻痫胶囊的临床疗效分析[J].中国中西医结合消化杂志. 2022;30(8)2022464384.
1374. 彭永剑, 王云滨, 郑君渭. 清热燥湿止泻汤联合美沙拉嗪肠溶片治疗湿热内蕴型溃疡性结肠炎临床研究[J]. 新中医. 2022;54(11)2022365785.
1375. 彭爱清, 王强, 朱倩倩. 柳氮磺吡啶与益生菌联合康复新液治疗活动期溃疡性结肠炎的临床疗效[J]. 临床合理用药杂志. 2023;16(12)2023244729.
1376. 徐春霞, 李荣军, 杨小丽, 王健, 杨燕. 从肺论治以荆芥连翘方结合针刺鬼眼穴治疗溃疡性结肠炎的临床观察[J]. 湖南中医药大学学报. 2023;43(02):338-342.
1377. 徐朝晖, 周乐, 蒋胜华. 健脾活血解毒汤辅助治疗缓解期溃疡性结肠炎效果观察[J]. 中国乡村医药. 2022;29(05):16-18.
1378. 戴亦娴, 张晓鸣, 蒋婷. 甘草泻心汤联合美沙拉嗪治疗溃疡性结肠炎的临床研究[J]. 中国肛肠病杂志. 2022;42(5)2022390237.
1379. 房修罗, 赵太云, 陆兴俊. 康复新液联合美沙拉嗪对溃疡性结肠炎活动期患者HMGB1、MCP-1、SOCS-3和Beclin1表达的影响[J]. 中华临床医师杂志-电子版. 2022;16(3)2022500580.
1380. 房修罗, 赵太云, 陆兴俊, 张洪领, 王胤. 复方苦参肠炎康片联合柳氮磺吡啶治疗溃疡性结肠炎的临床研究[J]. 现代药物与临床. 2022;37(07):1538-1542.
1381. 朱强, 季淦. 康复新液灌肠配合美沙拉嗪对溃疡性结肠炎患者炎症因子水平的影响[J]. 现代医学与健康研究电子杂志. 2022;6(24):95-98.
1382. 朱燕, 刘全喜, 王韶华, 刘大铭, 陈亮. 芪银三两三联合美沙拉嗪肠溶片干预轻中度溃疡性结肠炎临床疗效及安全性评价[J]. 中华中医药杂志. 2022;37(1)2022213244.
1383. 李一鸣, 李珩. 加味附子理中汤联合艾灸应用于脾肾阳虚型溃疡性结肠炎治疗的有效性分析[J]. 内蒙古中医药. 2022;41(11):62-63.
1384. 李东辉. 丹参酮ⅡA注射液联合美沙拉嗪治疗溃疡性结肠炎35例疗效观察. 中国肛肠病杂志. 2022;42(7)2022444291.
1385. 李克亚, 陆文洪, 王真权, 王军文. 乌梅丸联合美沙拉嗪对寒热错杂型溃疡性结肠炎患者的临床疗效[J]. 中成药. 2022;44(11):3493-3497.
1386. 李坤. 中药保留灌肠及综合护理治疗溃疡性结肠炎的效果[J]. 中国城乡企业卫生. 2022;37(1)2022225173.
1387. 李多, 刘祎梦, 王晓媛, 郝秀轻. 复方黄柏液联合美沙拉嗪对溃疡性结肠炎患者炎症因子和丙二醛的影响[J]. 河北北方学院学报（自然科学版）. 2022;38(8):24-26,30. doi:10.3969/j.issn.1673-1492.2022.08.005
1388. 李天伦, 黄敏, 李芳. 健脾益气升阳方联合美沙拉嗪肠溶片治疗复发型溃疡性结肠炎临床研究[J]. 四川中医. 2023;41(04):87-91.
1389. 李姗姗. 芍药汤合葛根芩连汤治疗溃疡性结肠炎的效果观察[J]. 中国社区医师. 2023;39(2)2023178637.
1390. 李娜, 郝海蓉, 任顺平, 刘媛, 刘竺华. 疏肝健脾汤联合授权教育治疗肝郁脾虚型溃疡性结肠炎的临床观察[J]. 中国民间疗法. 2022;30(11)2022332251.
1391. 李敏, 屈银宗. 白头翁汤灌肠联合美沙拉嗪治疗溃疡性结肠炎44例临床观察[J]. 湖南中医杂志. 2022;38(07):8-11+15.
1392. 李艳羽, 邹瑜. 地榆白及汤保留灌肠治疗大肠湿热型溃疡性结肠炎临床疗效观察[J]. 四川中医. 2022;40(9)2023154230.
1393. 李芳, 陈旭. 疏肝健脾颗粒联合中药灌肠与柳氮黄吡啶治疗溃疡性结肠炎（肝郁脾虚型）的临床疗效对比[J]. 中国医药指南. 2022;20(26):111-113.
1394. 李雪琦. 健脾消溃方联合美沙拉嗪肠溶片治疗脾虚湿蕴型活动期溃疡性结肠炎的临床观察[J]. 硕士. 湖南中医药大学; 2022.
1395. 杜亚平, 魏嫦, 黄粟, et al. 乌梅丸治疗溃疡性结肠炎的临床疗效及对免疫功能的影响[J].系统医学. 2022;7(22)2023173197.
1396. 杨周雨, 李秋阳. 柴胡疏肝丸联合美沙拉嗪治疗肝郁脾虚型溃疡性结肠炎的疗效[J]. 临床合理用药. 2023;16(15):81-84.
1397. 杨建军. 黄芩汤加减联合针刺辅助西医治疗活动期溃疡性结肠炎湿热蕴结证患者的效果[J]. 中国民康医学. 2022;34(15):88-90+94.
1398. 杨晓庆, 张俊, 黄振, 李道宽. 虎地肠溶胶囊联合美沙拉嗪肠溶片治疗溃疡性结肠炎的临床研究[J]. 齐齐哈尔医学院学报. 2022;43(16):1555-1558.
1399. 杨晓茹, 张艳霞, 王烨, 康亚军, 焦浩. 芍药四君健脾方加减口服联合灌肠治疗激素依赖型溃疡性结肠炎疗效及对炎性因子和免疫功能的影响[J]. 现代中西医结合杂志. 2023;32(06):828-831.
1400. 杨杰. 自拟化瘀清肠汤治疗溃疡性结肠炎的临床效果及对血清DAO、D-LA、TNF-α、IL-6、IL-8表达的影响[J]. 中华养生保健·学术版. 2022;40(6)2022207501.
1401. 杨瑞征, 武利萍, 谭莉霞, et al. 清肠化湿止泻汤佐治复发型溃疡性结肠炎急性发作期疗效观察[J]. 国医论坛. 2023;38(1)2023216924.
1402. 杨艳玲, 向黎莉, 陈鹏飞, 朱贤林. 加味葛根芩连汤对重度湿热型溃疡性结肠炎患者免疫功能及肠黏膜屏障功能的影响[J].辽宁中医杂志. 2023;50(3)2023239132.
1403. 林艺平. 解毒化浊促愈汤治疗溃疡性结肠炎的疗效及对白细胞介素-6、肿瘤坏死因子水平的影响分析[J]. 医学理论与实践. 2022;35(3):412-414.
1404. 柴小琴, 冯文哲, 雷彪, 牛魁, 石鹏. 三仁汤加减治疗急性期溃疡性结肠炎(湿热证)患者的临床研究[J]. 中国中医急症. 2022;31(3)2022214428.
1405. 梁峻尉, 白文筠, 王晓燕, 闫华, 迟莉丽. 基于"虚毒"理论观察中西医结合疗法对脾虚湿蕴型溃疡性结肠炎活动期的临床疗效[J]. 时珍国医国药. 2022;33(4):902-906.
1406. 段旭, 曾松林, 胡锦洋. 芪连结肠宁联合美沙拉嗪治疗活动期轻中度溃疡性结肠炎临床观察[J]. 山西中医. 2022;38(6):16-19.
1407. 殷绪胜, 梁榆明, 覃健, et al. 白头翁汤加减联合柳氮磺吡啶结肠溶胶囊治疗溃疡性结肠炎临床疗效观察[J]. 四川中医. 2022;40(9)2023154229.
1408. 潘姣. 参苓白术散联合美沙拉秦治疗溃疡性结肠炎的临床观察[D]. 硕士. 湖北中医药大学; 2022.
1409. 王倩倩, 张俊美. 中药灌肠联合美沙拉嗪治疗溃疡性结肠炎急性发作的临床观察[J]. 中国中医急症. 2022;31(08):1273-1275.
1410. 王倬, 陈强, 孔斌, 康现武, 赵士彭. 葛根芩连裁减方结合益生菌对溃疡性结肠炎血清IL-6、IL-12、IL-10和TGF-β水平的影响[J]. 中华中医药学刊. 2022;40(3)2022370296.
1411. 王富田. 益气调中散联合美沙拉嗪肠溶片治疗溃疡性结肠炎（脾虚湿蕴型）患者的临床疗效观察[J]. 硕士. 内蒙古医科大学; 2022.
1412. 王巴达日呼, 乌恩岳苏. 巴特日七味丸联合美沙拉嗪对溃疡性结肠炎患者的临床疗效[J].中成药. 2022;44(7)2022403479.
1413. 王德芳, 赵明, 李晓艳. 针刺结合黄芩汤化裁方治疗溃疡性结肠炎活动期(湿热蕴结证)的疗效观察[J]. 中医药导报. 2022;28(02):71-75.
1414. 王文静, 李俊雄, 梁洁意, 程灿昌, 童晶晶, 傅美丽. 自血穴位注射疗法联合西药治疗慢性复发型溃疡性结肠炎的临床价值研究[J]. 中外医药研究. 2022;1(11):75-77.
1415. 王文龙, 张薇. 自拟清凉止血灌肠方联合常规西药治疗溃疡性结肠炎大肠湿热证临床研究[J]. 中国中医药信息杂志. 2022;29(7):118-122.
1416. 王昱. 针药联合美沙拉嗪治疗寒热错杂型轻、中度溃疡性结肠炎的临床疗效观察[D]. 硕士. 山东中医药大学; 2022.
1417. 王晓瑜, 缪志伟, 张耀辉, 张春雨, 徐艳. 清肠汤联合美沙拉嗪治疗热盛血瘀型活动期溃疡性结肠炎患者的临床效果及其作用机制[J]. 中国医药导报. 2022;19(19):115-119.
1418. 王海涛. 中西药合用治疗溃疡性结肠炎疗效观察[J]. 实用中医药杂志. 2022;38(4):596-597.
1419. 王烨. 中药灌肠联合穴位贴敷治疗溃疡性结肠炎的疗效评价[J]. 中文科技期刊数据库（全文版）医药卫生. 2022;(10)
1420. 王烨, 张艳霞, 杨晓茹, 康亚军, 焦浩. 芍药四君健脾方联合中药灌肠治疗脾虚湿热型激素依赖溃疡性结肠炎疗效及对炎性因子和激素撤退的影响[J]. 现代中西医结合杂志. 2022;31(06):753-757+774.
1421. 王玥, 郭龙龙, 李欢, 黄雅慧, 姚佳. 健脾活血解毒汤联合美沙拉嗪治疗轻中度溃疡性结肠炎的疗效及其对MMP-9与ANCA的影响[J]. 临床和实验医学杂志. 2022;21(22)2023111428.
1422. 王磊, 罗瑞娟, 李娜, 姜莎莎, 许博佳, 柳越冬. 优化溃结方对溃疡性结肠炎临床疗效及血清炎症因子CRP、TNF-α的影响[J]. 中华中医药学刊. 2022;40(04):89-92.
1423. 王福忠. 参苓白术散加减辅治溃疡性结肠炎临床观察[J]. 实用中医药杂志. 2022;38(9)2022459697.
1424. 王贺, 李忠卓. 耳穴压豆联合通灌汤灌肠治疗溃疡性结肠炎临床观察[J]. 山西中医. 2022;38(11):44-45.
1425. 甄曙光, 张兆征. 仙桔汤合四逆散治疗溃疡性结肠炎的临床观察[J]. 深圳中西医结合杂志. 2022;32(13):36-39.
1426. 程钰权, 李明. 溃结Ⅰ号方中药保留灌肠联合西药治疗溃疡性结肠炎30例临床观察. 中国民族民间医药. 2023;32(09):115-118.
1427. 纪丽, 高宗跃. 针刺合中药保留灌肠联合西药治疗溃疡性结肠炎的疗效及对炎症因子水平、肠黏膜屏障指标的影响[J]. 中医研究. 2022;35(11):29-33.
1428. 罗志强. 补脾益肠汤治疗溃疡性结肠炎临床观察[J]. 光明中医. 2022;37(08):1358-1361.
1429. 翦闽涛, 杨赛, 高亚. 自拟芍药汤加减联合西药治疗溃疡性结肠炎的疗效及对患者免疫功能、血清IL-22、NF-kB水平的影响[J]. 四川中医. 2022;40(03):95-97.
1430. 耿晶, 曹瑜. 肠胃康颗粒联合美沙拉嗪肠溶片治疗大肠湿热型溃疡性结肠炎的效果[J]. 现代消化及介入诊疗. 2022;27(8):992-995.
1431. 苏海燕, 陈怀霞, 陈晓彤, 宋鹏. 四神丸合理中汤联合美沙拉嗪治疗溃疡性结肠炎作用机制研究[J]. 西部中医药. 2023;36(1)2023144923.
1432. 董曼, 尹平, 李炜, 杨红梅, 董粱. 美沙拉嗪联合内镜下中药治疗对改善轻中度溃疡性结肠炎患者炎症状态的研究[J]. 河北医药. 2022;44(21):3271-3274.
1433. 蒋庭德. 新加白头翁方治疗溃疡性结肠炎的疗效评价[J]. 智慧健康. 2022;8(27):169-172+181.
1434. 蔡怡, 贺明洁, 罗培培. 美沙拉嗪结合锡类散灌肠治疗溃疡性结肠炎的效果及对患者免疫功能和细胞因子的影响[J].中国当代医药. 2022;29(2)2022179146.
1435. 袁肖肖. 自拟温阳溃结方联合督灸治疗脾肾阳虚型溃疡性结肠炎的疗效观察[D]. 硕士. 山东中医药大学; 2022.
1436. 裴士会, 张健美, 吴云. 雷氏隔药隔姜灸脐疗法对缓解期溃疡性结肠炎患者的疗效研究[J]. 新疆医科大学学报. 2023;46(2):249-253.
1437. 谷圣青, 邵亮, 王子美, 李远, 赵良洁. 研究对溃疡性结肠炎患者实施自拟中药保留灌肠汤剂治疗的临床效果[J]. 世界复合医学. 2022;8(02):150-154.
1438. 谷圣青, 邵亮, 王子美, 李远, 赵良洁. 评价清热除湿中药灌肠方对溃疡性结肠炎(UC)患者快速诱导缓解和改善症状的有效性[J]. 中外医疗. 2022;41(36):38-41,50.
1439. 贾志春, 徐伟. 调中散联合美沙拉嗪治疗溃疡性结肠炎临床观察[J]. 光明中医. 2022;37(06):1052-1056.
1440. 赵仑. 清热利湿活血方口服配合中药灌肠对溃疡性结肠炎患者疗效及ICAM-1、VCAM-1影响[J]. 辽宁中医药大学学报. 2022;24(5)2022396659.
1441. 邱甜甜, 司秋霞, 彭树攀. 开玄汤治疗轻度溃疡性结肠炎患者的临床观察[J]. 中国中西医结合消化杂志. 2022;30(07):508-513.
1442. 邵伟. 溃结方Ⅰ号联合美沙拉嗪治疗大肠湿热型UC的临床观察[D]. 硕士. 黑龙江中医药大学; 2022.
1443. 邵梅, 陈曦, 张敏芬, 宣佶. 基于TLR/MyD88信号通路探讨枫蓼肠胃康颗粒联合益生菌治疗溃疡性结肠炎效果及作用机制[J]. 现代消化及介入诊疗. 2022;27(10)2023169573.
1444. 邹林焘, 陈鹏. 薏苡附子败酱散治疗溃疡性结肠炎临床疗效与机制研究[J]. 山东中医杂志. 2022;41(10)2022460477.
1445. 郑安锐, 齐云, 王品发, et al. 薏苡附子败酱散加味联合美沙拉嗪治疗溃疡性结肠炎寒湿瘀结型证的临床研究[J]. 时珍国医国药. 2022;33(11):2702-2704.
1446. 郑新平. 温肾健脾汤联合美沙拉嗪治疗溃疡性结肠炎临床研究[J]. 新中医. 2022;54(08):93-96.
1447. 郭孟凡, 李瑞艳, 尹小伟. 白头翁加味联合温针灸治疗溃疡性结肠炎的价值[J]. 哈尔滨医药. 2023;43(1):119-121.
1448. 陆文洪, 罗雯鹏, 肖戈. 芩柏加减方治疗溃疡性结肠炎效果及安全性观察[J]. 世界中医药. 2022;17(8):1110-1113.
1449. 陈志远. 加味葛根芩连汤治疗大肠湿热型溃疡性结肠炎疗效及对肠道菌群的影响观察[D]. 硕士. 安徽中医药大学; 2022.
1450. 雷洪峰, 程金荣, 龚光辉, 魏祖龙, 吴云翔. 地榆汤加减联合美沙拉嗪对湿热困阻型溃疡性结肠炎患者的临床疗效[J]. 中成药. 2022;44(11)2023102560.
1451. 项三妹, 李明. 参苓白术散加减治疗脾虚湿蕴型溃疡性结肠炎的疗效及对血清IL-10、PLT、Fib水平的影响[J]. 医学信息. 2022;35(8):162-164.
1452. 高燕, 王石红, 霍如晨, 梁宇晗, 何慧彬. 葛根红藤解毒汤联合美沙拉嗪肠溶片治疗溃疡性结肠炎浊毒内蕴证的疗效及对炎症因子的影响[J]. 河北中医. 2022;44(06):908-912.
1453. 鹿晓君, 高莉. 乌梅丸联合穴位埋线治疗寒热错杂证溃疡性结肠炎疗效观察[J]. 中国临床研究. 2023;36(01):117-121.
1454. 黄六胜, 陈婷, 余绪超, 肖慧荣, 邓思洋. 清肠凉血方直肠滴入联合穴位埋线对活动期溃疡性结肠炎的临床疗效观察[J]. 实用中西医结合临床. 2022;22(12)2022478769.
1455. 黄婉萍. 加味白头翁汤保留灌肠联合美沙拉嗪口服治疗大肠湿热型溃疡性结肠炎的临床疗效观察[D]. 硕士. 福建中医药大学; 2022.
1456. 黄志高, 张春霞, 杨丽丽. 隔附子饼灸联合美沙拉嗪治疗溃疡性结肠炎的疗效研究[J]. 内蒙古中医药. 2023;42(1):16-18.
1457. 黄惠红, 王群花, 方芳, 冯强. 固肠丸治疗脾肾阳虚型溃疡性结肠炎临床观察[J]. 四川中医. 2023;41(01):111-114.
1458. 黄飞鸿, 张全辉, 邓永文, 肖亮. 参苓白术散加减对溃疡性结肠炎的疗效及对IL-6水平的影响[J]. 江西中医药. 2023;54(4):39-41.
